# Development of the CHILD‐SHOE Reporting Checklist: A Scoping Review and Modified Delphi Study to Support Reporting in Children's Footwear Research

**DOI:** 10.1002/jfa2.70065

**Published:** 2025-07-09

**Authors:** Cylie M. Williams, Melanie Farlie, Jessica Kolic, Stewart C. Morrison, Kade Paterson, Matthew Hill, Jason Bonacci, Marise C. Breet, Simone Cranage, Shane V. Caswell, J. J. Hannigan, Alexis Herbaut, Karsten Hollander, Peter O. Ibikunle, Rachel A. Kennedy, Pui Wah Kong, Jayishni N. Maharaj, Natalie Mazzella, Shannon E. Munteanu, Liria A. Okai‐Nóbrega, Gabriel Gijon‐Nogueron, Jan Plesek, Hassan Saeedi, Jasper W. K. Tong, Abebayehu Tora, Ranel Venter, Yasin Yurt, Helen Banwell

**Affiliations:** ^1^ School of Primary and Allied Health Care Monash University Frankston Australia; ^2^ Allied Health and Human Performance University of South Australia Adelaide Australia; ^3^ Monash Centre for Scholarship in Health Education Monash University Clayton Australia; ^4^ School of Life Course and Population Sciences King's College London UK; ^5^ Centre for Health, Exercise and Sports Medicine The University of Melbourne Melbourne Australia; ^6^ Centre for Biomechanics and Rehabilitation Technologies Staffordshire University Staffordshire UK; ^7^ Centre for Sport Research Institute for Physical Activity and Nutrition Deakin University Geelong Waurn Ponds Campus Geelong Australia; ^8^ Division of Sport Science Department of Exercise, Sport, and Lifestyle Medicine Faculty of Medicine and Health Sciences Stellenbosch University Stellenbosch South Africa; ^9^ Monash Health Clayton Australia; ^10^ School of Kinesiology College of Education and Human Development George Mason University Fairfax Virginia USA; ^11^ Program in Physical Therapy College of Health Oregon State University ‐ Cascades Bend Oregon USA; ^12^ Decathlon SportsLab Lille France; ^13^ MSH Medical School Hamburg Institute of Interdisciplinary Exercise Science and Sports Medicine Hamburg Germany; ^14^ Nnewi Campus Nnamdi Azikiwe University Awka Nigeria; ^15^ Department of Neurology The Royal Children's Hospital Parkville Australia; ^16^ Neurosciences Clinical Sciences Murdoch Children's Research Institute Parkville Australia; ^17^ National Institute of Education Nanyang Technological University Singapore Singapore; ^18^ Australian Centre for Precision Health and Technology Griffith University Nathan Australia; ^19^ School of Health Sciences and Social Work Griffith University Nathan Australia; ^20^ Discipline of Podiatry School of Allied Health, Human Services and Sport La Trobe University Victoria Australia; ^21^ Aname Baby Design Belo Horizonte Brazil; ^22^ Department of Nursing and Podiatry University of Malaga Malaga Spain; ^23^ Department of Human Movement Studies University of Ostrava Moravská Ostrava a Přívoz Czech Republic; ^24^ Rehabilitation Research Center Department of Orthotics and Prosthetics School of Rehabilitation Sciences Iran University of Medical Sciences Tehran Iran; ^25^ Allied Health Office KK Women’s and Children’s Hospital Singapore Singapore; ^26^ Wolaita Sodo University Soddo Ethiopia; ^27^ Faculty of Health Sciences Physiotherapy and Rehabilitation Department Eastern Mediterranean University Famagusta Türkiye

**Keywords:** consensus, foot, gait, outcome measures, paediatric

## Abstract

**Background:**

Inconsistent reporting of interventions and outcomes is a key barrier to research translation. Children's footwear research is often inconsistently reported as there are no standards or recommendations on what to report or consensus on which outcomes are important. The primary aim of this research was to develop expert consensus in children's footwear features and descriptions for research reporting. The secondary aim focused on consensus building of outcome measures relating to footwear in research in children. The outcome of this study was to develop a reporting checklist and guidance for researchers who are conducting children's footwear studies.

**Methods:**

This was a three‐round modified Delphi survey informed by a scoping review. We searched four databases to enable data extraction from 109 records related to children's footwear research. These data established the basis for Round 1. Authors were identified through the scoping review and invited to participate. In Round 1, participants rated the appropriateness of domains relating to reporting footwear descriptions and features and outcomes. Outcome measures were organised against a childhood adaptation of the International Classification of Functioning, Disability and Health (ICF)—F‐words in childhood disability domains. Consensus and agreement levels were set at 70%. Where 50%–69% of participants agreed, the item was returned for rating in Rounds 2 and 3.

**Results:**

There were 33 participants who responded to Round 1 and 29 (88%) in both subsequent rounds. Participants agreed on 20 statements that researchers should use to describe children's footwear and their features. All ICF domains met consensus for outcome collection. There were 17 outcome measures that participants agreed should be used in the future when a researcher's aim aligns with specific domains. Where no specific outcome measures reached consensus or agreement within a domain, a statement was developed to guide researcher choice in the subsequently developed checklist.

**Conclusion:**

Participants reached consensus on the essential footwear characteristics and descriptions that should be consistently reported in children's footwear research. This enabled us to produce a list of preferred outcome measures. Using this checklist can support future research through collection and reporting of comparable data.

**Abbreviations**
cm: centimetres
EMG: electromyography
ICF: International Classification of Functioning, Disability and Health
n: number
sec: seconds

## 1 Background

Parents and caregivers often seek advice about children's footwear fit and impact on foot and lower limb development [1]. Both parents and health professionals hold beliefs that footwear can positively or negatively impact the growing foot and lower limb morphology and function [2]. These perceptions are frequently shaped by observations and marketing but also from fashion trends and personal beliefs rather than being founded on evidence [3, 4]. Two recent systematic reviews exploring the impact of footwear and specific features on children's walking and running reported this challenge [5, 6]. Their results were limited by inconsistent descriptions of footwear and diverse outcome measures. These inconsistencies confound the ability of researchers to determine optimal methods to conduct and synthesise children's footwear research.

The children's footwear industry has a projected market size valued at approximately $55 billion (USD) [7]. A major market driver is repeated shoe purchasing due to childhood growth. However, there are additional drivers that hinge on the marketing of the benefits one type of footwear may have over another. This is despite limited evidence indicating a child's physiological or developmental wellbeing being impacted by specific footwear features, or the influence of shoes on the growing lower limb, other than the importance of good fit [8, 9, 10]. Appropriate footwear fit improves walking speed and can reduce some step variability regardless of a child's ability [11]. Additionally, there is an interest in utilising footwear as a therapeutic intervention for children with lower limb health or gait concerns [12].

There is more known about the impact of footwear on adult's lower limb health and biomechanics compared to paediatric populations [13]. Footwear research in adults links poorly fitting footwear with bone, joint and soft tissue related injuries [13]. Also, studies with adults have demonstrated the potential for footwear to change forces through the lower limb [14], reduce tissue damage [15] and potentially reduce falls [16]. Continued investment in the adult sporting footwear industry demonstrates footwear is also considered a method of performance enhancement [17]. This variation in knowledge about footwear effects across the different life stages provides opportunities for future research.

Inconsistency in reporting the characteristics of children's footwear and measurement of footwear research outcomes are major barriers to research synthesis and translation. Adult footwear research has several tools enabling consistency in reporting [18], such as the definition for minimalist footwear [19] or the footwear assessment tool [20]. Children's footwear is not a scaled down version of adult footwear; therefore, these tools are not translatable nor are there recommended outcome measures. Researchers need to work together to adopt ways to report their research so that data can be pooled to strengthen future recommendations. The primary aim of this research was to develop consensus in children's footwear descriptions and features for research reporting. The secondary aim focused on consensus building of outcome measures relating to footwear research in children. The outcome of this consensus study was to develop a reporting checklist and guidance for researchers who are conducting children's footwear studies.

## 2 Methods

### 2.1 Design

Our study approach involved a three‐round modified Delphi survey method informed by a scoping review. The Delphi methodology involved inviting experts to participate in sequential questionnaires (Rounds), with the aim of eventual consensus of opinions [21]. Although expert opinion is considered a lower form of evidence, it is considered appropriate as the first step in research in the absence of existing guidance [22]. Delphi was modified from traditional methods by online data collection only and through the development of Round 1 as outlined in the procedure. Monash University Human Research Ethics Committee (27514) approved this research, and it was prospectively registered with Enhancing the QUAlity and Transparency Of health Research (EQUATOR) [23]. We used the Conducting and REporting DElphi Studies (CREDES) checklist to guide reporting our research [24].

### 2.2 Participants

Participants were recruited using purposive sampling. Eligible participants were those who had published peer‐reviewed research relating to children's footwear, identified through the scoping review. We determined eligibility in this way to ensure we invited ‘experts’, according to Delphi methodology, due to their research experience and potential contribution to children's footwear research.

### 2.3 Procedure

We initially conducted a scoping review, directed by the preferred reporting guidelines for scoping reviews [PRISMA‐ScR]. This review was not prospectively registered; however, it enabled the identification of potential experts to guide consensus and was conducted to develop the questions for Round 1 [25]. Searches were conducted using Medline Ovid, CINAHL, EBM, Emcare and AMED (Allied and Complementary Medicine) from inception and up to 24th of March 2022. The searches were rerun up to 20th of May 2024 to ensure current data extraction. We did not apply any study design limits to data searches but applied limits to human participants and English language. The search strategy was broad and used the MeSH terms: (Child OR Infant OR Adolescent OR p*ediatric) AND (Shoes OR Shod OR footwear; Supporting Information S1: Appendix 1—Search strategy). Inclusion criteria included peer‐reviewed literature of any quantitative study design, description of at least one footwear type or feature or at least one outcome relating to the footwear worn and with a mean age of participants < 18 years. Where studies used footwear modified specifically for the study design, only nonmodified footwear data were extracted. Qualitative articles were excluded.

**FIGURE 1:**
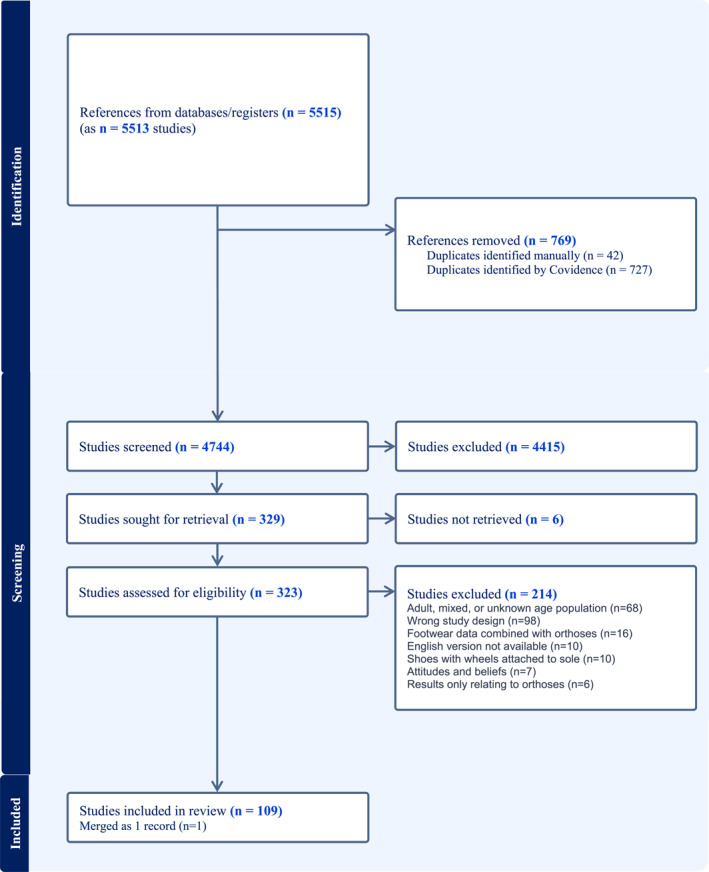
PRISMA flowchart.

Data describing footwear characteristics (e.g., features and descriptions) identified in the 109 records (Figure 1) were thematically synthesised, where possible, according to published footwear‐related characteristic frameworks [12, 26]. Outcomes were synthesised using the International Classification of Functioning, Disability and Health (ICF) childhood adaptation. This adaption has been described using F‐words—function, family, fitness, fun, friends and future [27]. Data extraction was undertaken and checked by two authors (J.K. and C.W.) and verified by a research assistant. Five authors (M.F., K.P., S.M., M.H. and H.B.) confirmed domain alignment before data were presented to the participants [12, 26] (Supporting Information S2: Appendix 2—Data extraction).

Potential participant contact details were extracted from their publications prior to being emailed an invitation, which included study details and an option to participate. If an email was invalid, ResearchGate (www.researchgate.net) was used as a secondary contact source. Participants contacted via ResearchGate received messages through the platform's messaging service. Those invited were informed of the publication that led to their invitation and were encouraged to forward the invitation to eligible co‐authors of only that publication. Participants provided written informed consent signifying ongoing commitment to responding to all Delphi rounds. Participants were asked to not engage in intra‐panel communication and to keep their responses confidential at each round. Participants were offered acknowledgement or authorship in the final paper pending their willingness and ability to meet the authorship criteria (see Contribution Statement).

Questions in each round were presented to participants through a purpose‐built survey using the online survey platform Qualtrics software (Qualtrics, Provo, UT, USA). The survey was piloted with two members of the research team who were not child footwear researchers and language adaptations made based on their feedback. The data were linked during the rounds through participant‐provided email only. Rounds were pre‐planned as being open for 2–4 weeks, and participants were emailed weekly reminders to maximise retention throughout the study. All participants were offered their individualised responses at the beginning of each subsequent round. Full results were provided to participants completing all three rounds.

In Round 1, participants provided demographic information including gender, country of residence, highest qualification, number of peer‐reviewed publications and the number of those related to children's footwear. This allowed an understanding of participants' breadth of experience.

Following this, participants were then presented with questions relating to the primary aim. They were asked to respond to each element of interest or domain identified in the scoping review. Survey logic was used to present items under each element or interest or domain based on the participants response. For example, if a participant considered *Footwear Type* should SOMETIMES or ALWAYS be reported, they were then presented with all the footwear types extracted to rate agreement. Whereas if they responded with NEVER, they moved to the next question.

We followed the same process for the secondary aim. Participants were presented with domains aligning with the relevant F‐word. If participants agreed it should SOMETIMES or ALWAYS be reported, they were then presented with extracted outcomes aligning to that domain to respond in agreement with its inclusion. Open text boxes were included at the end of each section to allow participants to justify their rating or suggest alternative wording. The full Round 1 survey and logic is in Supporting Information S3: Appendix 3 and a summary of domains and elements in Table 1.

**TABLE 1:** Domains and elements.

Domain	Elements
Footwear descriptions	Brand name
	Includes image of the footwear
	Commercial availability
	Footwear retail cost
Footwear features	Footwear type
	Components of the footwear
	Composition of the footwear
Function	Spatiotemporal measures
	Kinematics and kinetics
	Plantar pressure
	Foot features
	Balance and gross motor function
	Presence of infection
Fitness	Physical activity measures
	Electromyography (EMG)
	Endurance
Friendship	Quality of life measures
Fun	Comfort
	Body image perception relating to feet appearance
Family	Social factors
Future	School attendance
	Longitudinal impact of footwear on foot shape
	Academic performance
	Skin and nail trauma linked to footwear usage

Data responses were initially analysed by two researchers (J.K. and C.W.). Round 1 consensus was achieved when 70% or more participants agreed on an item. Where 50%–69% of participants indicated affirmative responses, the information was re‐presented in Round 2 (Supporting Information S4: Appendix 4). Where less than 50% of participants indicated affirmation on any item, these were excluded from future rounds. These consensus thresholds align with established modified Delphi methodology [28, 29].

Round 2 followed the same thresholds for agreement and guided the development of Round 3 (Supporting Information S5: Appendix 5). Any new items suggested in Round 1 were incorporated in Round 2 for review. Participants were asked to reconsider these items and indicate their level of agreement. In Round 3, any item not meeting the 70% threshold was excluded.

Participants who did not complete the entire round were contacted and offered a further 2‐week extension. Those who did not complete a round were excluded from subsequent rounds. An a priori decision was to cease the Delphi if there was a drop in participant response below 70%.

### 2.4 Analysis

We described the synthesis of data in Round 1 within the procedure. We calculated the counts and percentages for each round within Microsoft Excel 2018 (Microsoft Corp, Redmond Washington). We also used Excel to track response rates across rounds.

## 3 Results

**FIGURE 2:**
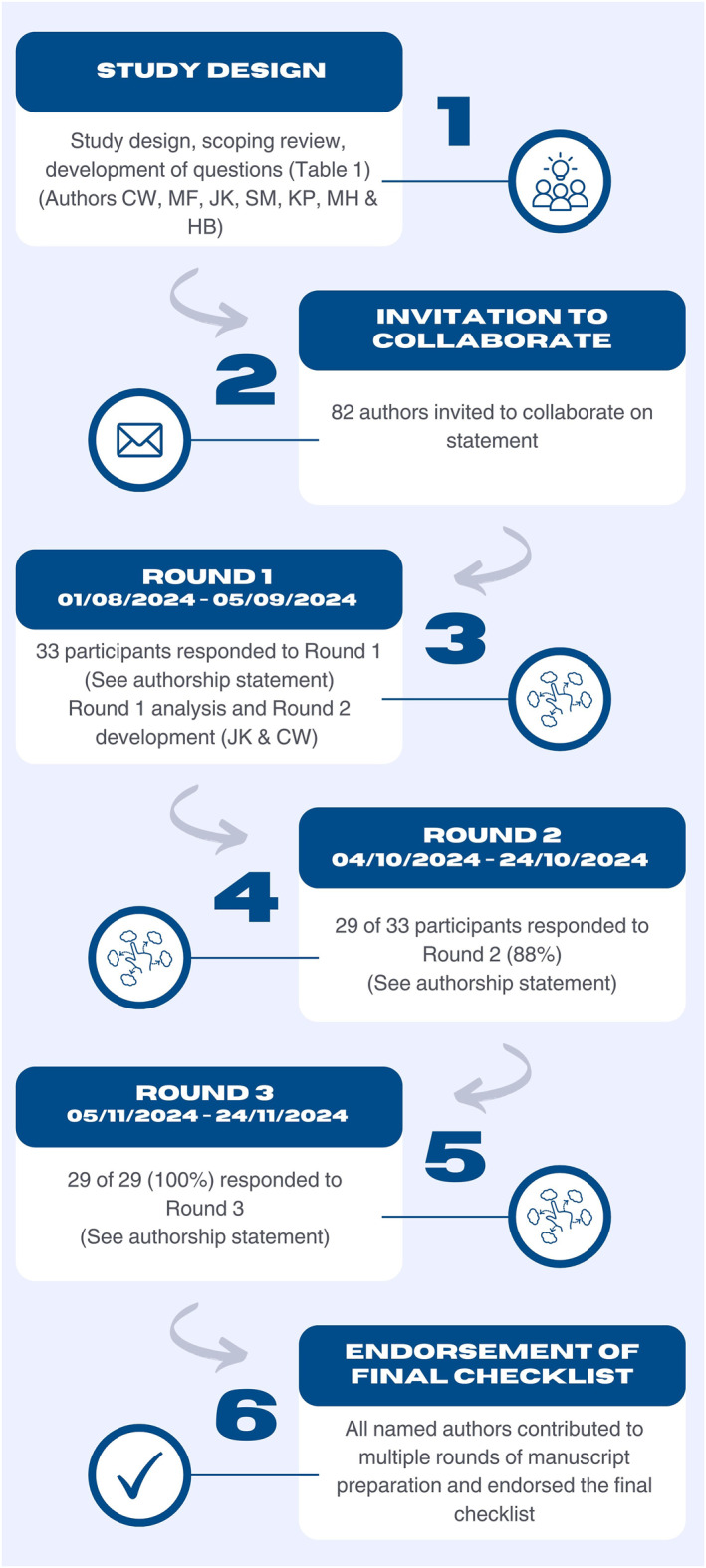
Delphi study flow including author and participant involvement.

Of the 82 authors invited, 33 consented to participate in Round 1 (Table 2). There was an 88% (29 of 33 participants) retention rate for Round 2 and 100% (29 of 29 participants) retention rate in Round 3 (Figure 2. Delphi study flow including author and participant involvement). The three rounds were completed between 1st August 2024 and 24th of November 2024.

**TABLE 2:** Summary demographics of participants completing Round 1 (*n* = 33).

	*n* (%), median (range)
Participant gender (Man)	20 (61%)
World region	
Africa	6 (19%)
Asia	4 (12%)
Europe	10 (30%)
North America	2 (6%)
Oceania	10 (30%)
South America	1 (3%)
Highest research qualification/degree	
PhD	28 (85%)
Masters	5 (15%)
Profession/Clinical training	
Podiatrist	12 (37%)
Physiotherapist/Physical therapist	6 (18%)
Sport scientist	6 (18%)
Biomechanist	4 (X%)
Sociologist/Public health researcher	2 (6%)
Orthotist/Prosthetist	1 (3%)
Sports medicine physician	1 (3%)
Biokineticist/Exercise physiologist	1 (3%)
Publications	
Total career publications	30 (2–190)
Children's footwear specific publications	2 (1–5)

**FIGURE 3:**
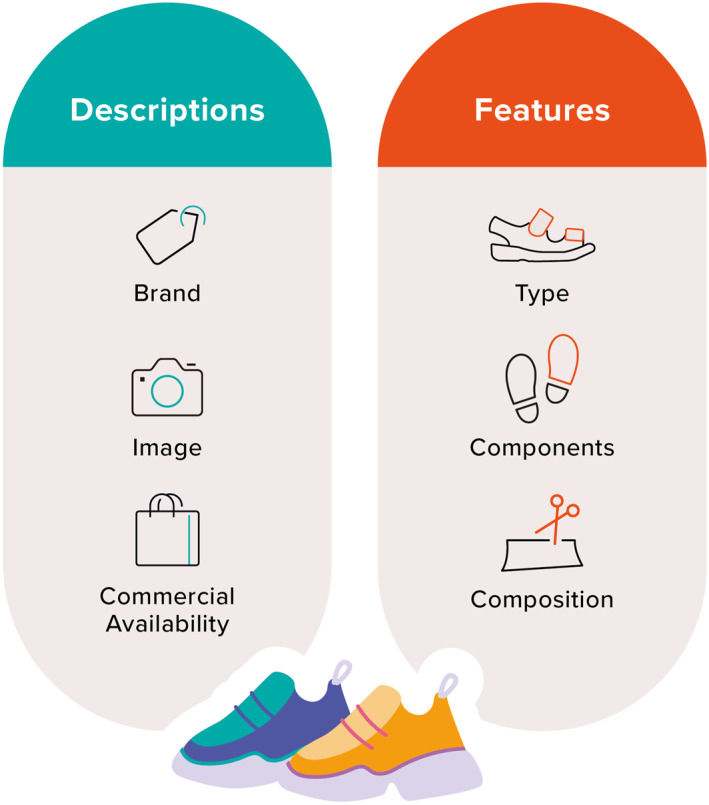
Footwear descriptions and features.

A graphical summary of the footwear description and feature domains that met consensus are in Figure 3. There were three footwear description elements and three footwear feature elements meeting consensus (Table 3). Within footwear descriptions, participants identified brand name, image of the footwear and its commercial availability as essential elements in reporting research about children's footwear. The three footwear feature elements and their element descriptions meeting consensus or agreement included footwear type (*n* = 9 element descriptors), components of the footwear (*n* = 8 element descriptors) and composition of the footwear (*n* = 2 element descriptors) (Table 3).

**TABLE 3:** Statements supporting minimum reporting requirements of footwear features and descriptions domains and elements within each.

Domain	Elements	Round 1 (%) of 33	Round 2 (%) of 29	Round 3 (%) of 29
Footwear descriptions	Brand name	76%		
	Includes image of the footwear	100%		
	Commercial availability	73%		
	Footwear retail cost	58%	31%	
Footwear features	Footwear type	100%		
	Sandal, flip flops or slides	94%		
	Slipper/Indoor shoe	81%		
	Biomimetic, functional or minimalist	81%		
	General or casual shoe	56%	45%	
	Mary Jane, ballet flat or Td‐bar shoe	56%	79%	
	Boots	78%		
	Pre‐walker or soft soled shoe^a^	63%	69%	41%
	School shoe (oxford style)	84%		
	Therapeutic footwear (medical/orthopaedic)	75%		
	Sport specific (with sport listed) shoe	91%		
	Sneakers, runners, trainers or sport/athletics shoes	97%		
	Outdoor shoe	34%		
Footwear features	Components of the footwear	100%		
	Heel counter presence and/or it is stiffness	87%		
	Upper of shoe covers full or part of foot	94%		
	Outsole with/without separate heel	61%	66%	48%
	Sole flexibility	90%		
	Insole materials in shoe	65%	59%	51%
	Topline of shoe in relation to the ankle (e.g., high, mid or low cut)^a^	55%	62%	86%
	Mass (e.g., grams) of shoes	71%		
	Sole shape (including last) of shoe	42%		
	Fixtures (e.g., velcro and laces) of shoe	81%		
	Toe box (shape and/or height) of upper	52%	79%	
	Pitch, drop and/or stack of outsole	77%		
	Slip resistance of outsole	29%		
	Minimalist index of footwear	39%		
	Presence, amount and location of sole rocker	42%		
	Footwear colour	6%		
Footwear features	Composition of the footwear	100%		
	Glue and/or adhesives	6%		
	Upper material	71%		
	Sole material	74%		

*Note:* Colour code: Green: where 70% or more participants agreed similarly and accepted, Orange: where 50%–69% of participants agreed similarly and processed to next round and Red: where < 50% of people and item or element was removed from final CHILD‐SHOE Reporting Checklist.

^a^ Indicates alternative wording presented to panel members.

**FIGURE 4:**
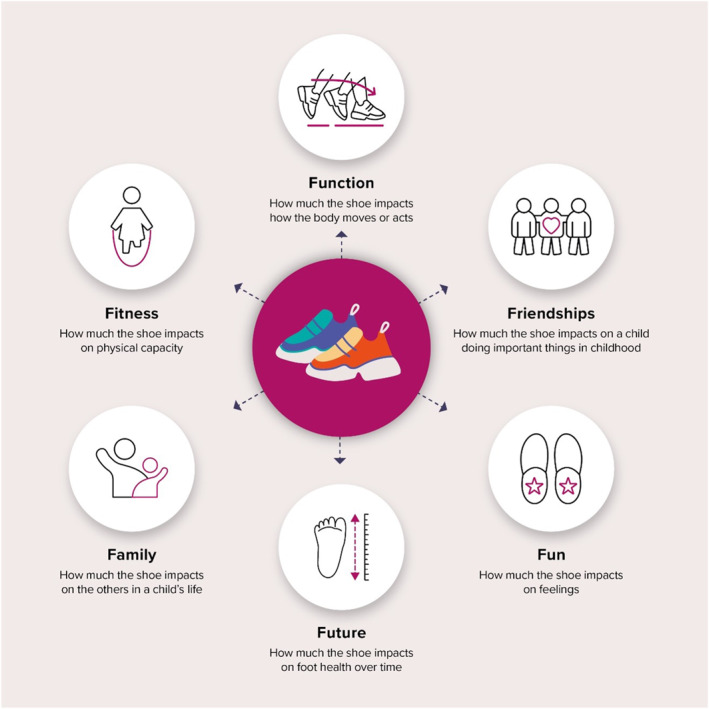
Outcome measure F‐word domains.

The six ICF F‐words (Table 4) met consensus in Round 1, and Figure 4 provides a graphical summary of these overarching domains and their description. The six outcome measure groups in the *FUNCTION* domain met consensus or agreement. These included spatiotemporal measures (*n* = 4 outcome measures), kinematics and kinetics (*n* = 5 outcome measures), plantar pressure (researcher preference statement), foot features (*n* = 1 outcome measure), balance and gross motor skill (researcher preference statement) and presence of infection.

**TABLE 4:** Statements supporting minimum reporting requirements of footwear research outcomes relating to aim of research and descriptions.

Domain	Outcome measures	Round 1 (%) of 29	Round 2 (%) of 29	Round 3 (%) of 29
Function	Spatiotemporal measures	100%		
	Velocity (meters/second)	87%		
	Cadence (steps/second)	81%		
	Stride length (cm)	74%		
	Step length (cm)	74%		
	Base of support (cm)	29%		
	Stance time (sec)	42%		
	Step time (sec)	26%		
	Toe in/toe out angles (degrees)	52%	59%	38%
	Step width (cm)	42%		
	Double/Single support	35%		
	Contact time (sec)	42%		
	Stance phase %	45%		
	Swing phase %	39%		
	Stride time (sec)	35%		
	Number of steps	29%		
Function	Kinematic and kinetic measures	100%		
	Hip angles	68%	76%	
	Knee angles	77%		
	Ankle angles	77%		
	Foot angles	77%		
	Ground reaction force	61%	72%	
	Vertical loading	23%		
	Joint power	19%		
	Impulse	10%		
	Peak pressure (kPa)	13%		
	Centre of mass displacement	16%		
	Ground reaction time	10%		
Function	Plantar pressure	97%		
	Angle of gait	23%		
	% pressure at fore/hind foot	60%	52%	35%
	mean pressure	27%		
Function	Foot features	100%		
	Foot size	77%		
	Skin/Nail trauma	27%		
	Muscle size	4%		
	Weissflog index	4%		
	Ankle range of motion	23%		
	Resting calcaneal stance ankle	27%		
	5th toe degree	12%		
	Instep height	12%		
	Arch Index	35%		
	Foot posture index	35%		
	Hallux valgus angle	35%		
	Clarks angle	8%		
Function	Balance and gross motor function	97%		
	COP displacement	53%	62%	41%
	Functional reach test	23%		
	Flamingo stance	20%		
	Number of falls	47%		
	Single leg stance	23%		
	Modified balance error scoring system	27%		
	Bruininks–Oseretsky test of motor proficiency	23%		
	Balance error score	13%		
	Four square step test	17%		
	Standing long jump	27%		
	Test of gross motor development	30%		
	Hoffer ambulation score	7%		
	Tip toe walking	23%		
	Dynamic hop	20%		
	Edinburgh gait scale	10%		
	Y test	7%		
	Timed up and go	27		
	Lower limb muscle strength	27%		
Function	Presence of infection	77%		
Fitness	Physical activity measures	97%		
	Steps per day	53%	79%	
	Physical activity diary	50%	55%	41%
	Functional mobility scale	40%		
	Ball contact	10%		
	Moderate to vigorous physical activity	43%		
	VO‐2	13%		
	Energy expenditure	27%		
	Borg rating of perceived exertion	23%		
Fitness	EMG	74%		
Fitness	Endurance	87%		
	6 min walk test	48%		
	10 min walk test	26%		
	Statement of choice^a^		83%	
Friendship	Quality of life measures	97%		
	Oxford ankle foot questionnare	43%		
	Knee injury and osteoarthritis outcome score	17%		
	Anterior knee pain scale	20%		
	Youth quality of life	47%		
	Paediatric quality of life inventory	47^		
	Statement of choice^a^		83%	
Fun	Comfort	97%		
	Perception of shoe fit	50%	62%	31%
	Shoe fit measured with a fit device	73%		
	Number of injuries	17%		
	Pain (binary/visual analogue scale)	63%	69%	62%
	Footwear assessment score	23%		
	Footwear comfort	60%	97%	
	Shoe wear distortion	23%		
Fun	Body image perception relating to feet look	81%		
Family	Social factors	97%		
	Wear time	97%		
	Patterns of ownership	33%		
	Recommendations from health professionals	40%		
	Parent level of education		59%	27%
	Requires support with putting on and taking off footwear		52%	37%
Future	School attendance	81%		
Future	Longitudinal impact of footwear on foot shape	100%		
Future	Academic performance		41%	
Future	Skin and nail trauma linked to footwear usage		69%	62%

*Note:* Colour code: Green: where 70% or more participants agreed similarly and accepted, Orange: where 50%–69% of participants agreed similarly and processed to next round and Red: where < 50% of people and item or element was removed from final CHILD‐SHOE Reporting Checklist.

^a^ Indicates alternative wording presented to panel members in Rounds 2 and 3.

The three outcome measure groups in the FITNESS domain meeting consensus or agreement included physical activity outcome measurement (*n* = 1 outcome measure), electromyography (EMG; researcher preference statement) or endurance (researcher preference statement). Participants recommended researcher preference based on age and stage of the child and research aim.

Participants agreed that the FRIENDSHIP domain should collect information about the child's quality of life and that the tool to collect this information be based on the age and developmental stage of the child and research aim.

Comfort and body image perception relating to look of feet were two outcome measures meeting consensus in the FUN domain. Participants agreed researchers should report outcome measures of shoe fit measured with a device and footwear comfort under the comfort element. Whereas they agreed that researchers should choose the appropriate tool or method to report body image perception relevant to the child's age and stage or research aim.

Finally, wear time met consensus under social factors in the FAMILY domain. School attendance and longitudinal impact of footwear on foot shape were outcome measures meeting consensus under the FUTURE domain.

The final checklist was circulated to all authors to ensure appropriate wording for use when reporting research relating to describing children's footwear and choice and reporting of outcomes (Figure 5 Final Checklist, Supporting Information S6: Appendix 6 Editable version).

FIGURE 5CHILD‐shoe checklist.

**Figure:**
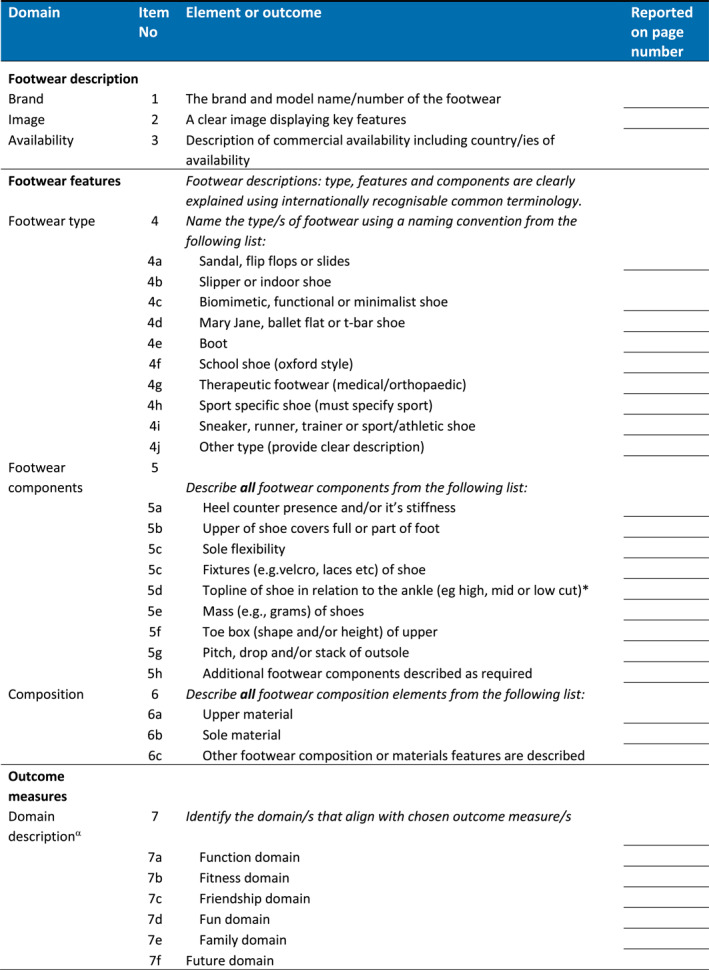

**Figure:**
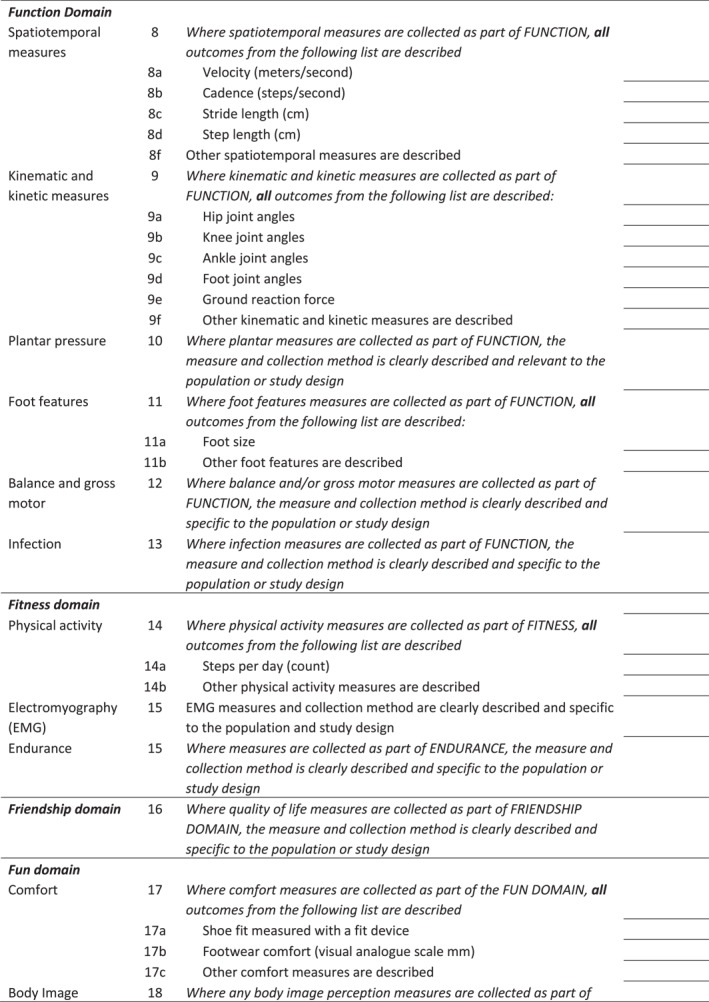

**Figure:**
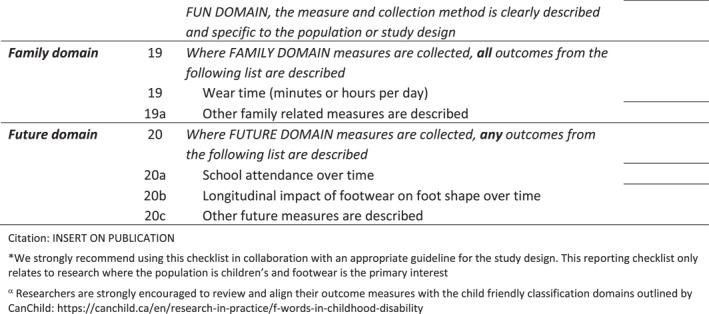

## 4 Discussion

This study, underpinned by an extensive scoping review and developed with experienced researchers in the field, presents the first guidance to support consistency in the measurement and reporting of children's footwear research. The CHILD‐SHOE Reporting Checklist incorporates 20 footwear descriptions and outcome measures considered essential by experienced children's footwear researchers. This checklist can guide the choice of outcome measures during research design, in addition to standardising reporting requirements for publishing research related to children's footwear. Key to this is that outcome measures align with the ICF, supporting researchers to measure things that matter to families and look more broader beyond the functional impact of footwear, increasing not only the consistency but also scope and relevance of published evidence going forward.

Importantly, this adds to a growing body of recommendations and guidelines supporting consistency in the measurement on reporting of research related to children's footwear. These include an international taxonomy that defines nomenclature [26] and consensus recommendations for classifications and indications of use in children's therapeutic footwear [12]. However, it also highlights potential gaps in research consistency, such as where consensus could not be reached on the tools required to measure several key outcome measures, including quality of life or balance and gross motor skill. This is unsurprising given the diversity of instruments to measure these domains (including some specific to age or disease specific tools), but it does inhibit the ability to build large data sets, particularly as children's outcomes change over their development. It also highlights the opportunity to align research outcomes with those recommended by organisations, such as OMERACT (https://omeract.org/) or ICHOM (https://www.ichom.org/), that are focused on outcome measurement sets that align with diseases [30]. Furthermore, some of the outcome measures that reached consensus require use of specific measurement tools over others, such as those that identify joint angles over centre of mass or peak pressures. Although this may be a barrier for some research, it may also assist researchers to seek and plan appropriate equipment and protocols. This may eventuate in the development of much‐needed normative data using this measurement tools.

It is expected that further refinement will be required to improve usability and outcomes contained within the CHILD‐SHOE Reporting Checklist. Specifically, this may include defining specific elements where nomenclature remains inconsistent. This has already been noted as a concern for minimalist, functional and biomimetic footwear for children [31, 32, 33, 34]. Similarly, although agreement was reached on reporting foot size, there was no discussion whether it should be reported in metric, imperial measures or as raw length and/or/width. As technology advances, there will also be a requirement to revisit agreement on preferred measures across most domains (e.g., three‐dimensional scanning may direct foot volume as a measure of interest or wearable sensors may alter kinematic or kinetic outcomes) [35]. Similarly, as the body of evidence and adherence in utilising this recommended checklist develops, further refinements may narrow down the most essential descriptions and outcome measures, reducing the burden on researchers.

However, for now, it is recommended that users of the checklist report against all essential footwear descriptions and feature domains, consider the data collected against the ‘menu’ of outcome measures, and report the impact within the F‐words domains as appropriate to their research question. This will be an important step in future research synthesis, allowing interpretation of research to be meaningful to the population and impactful for consumers, including parents, and caregivers, footwear manufacturers, retailers and those who determine polices around footwear (e.g., such as shoes worn in schools or guidelines for young athletes) [36]. Currently, most of these ‘policy papers’ rely on evidence synthesis, which has been shown to be negatively impacted by the diversity in how footwear is described and assessed during research.

As with all research, there are key limitations to this study. Firstly, the search strategy that directed our scoping review did not include forward and backward searching or the synthesis of data, meaning our theming may be open to bias. We attempted to minimise this bias through transparency to the broader research team at each round, ensuring missing items could be suggested. Secondly, we acknowledge the checklist developed is based on expert opinion, and that no consumers were involved in this research. However, many of the research team are also parents who purchase footwear for their children meaning we are both researchers and consumers. We acknowledge that good research is co‐designed with those most impacted by the outcomes, and for this study, we considered those most impacted to be researchers. The checklist does provide opportunities for researchers to co‐design their research with children and families, meaning the outcomes from this subsequent research potentially include the consumer voice and report on factors important to them. The use of an international panel of experts is a strength in terms of global relevance, albeit we acknowledge that 60% of the panel were from Europe or Oceania. Cultural footwear practices may have biased checklist choices made by the expert researchers involved. Remaining anonymous and keeping statements confidential are suggested to minimise collusions of results and minimise bias in gathering agreement. We guarantee that as an author group no collusion occurred; however, it is unknown what impact collaborative discussion may have had on the final checklist [22].

## 5 Conclusion

This research used a modified Delphi consensus methodology to identify key footwear descriptions and features experts agreed should be reported in future children's footwear research. This enabled us to produce a list of preferred outcome measures. Using this checklist can support future research through collection and reporting of comparable data.

## Author Contributions

**Cylie M. Williams:** conceptualization, supervision, methodology, investigation, project administration, formal analysis, writing – original draft preparation, writing – reviewing and editing. **Melanie Farlie:** supervision, methodology, investigation, validation, writing – reviewing and editing. **Jessica Kolic:** methodology, investigation, validation, project administration, formal analysis, writing – reviewing and editing. **Stewart C. Morrison:** conceptualization, methodology, investigation, validation, writing – reviewing and editing. **Kade Paterson:** conceptualization, methodology, investigation, validation, writing – reviewing and editing. **Matthew Hill:** methodology, investigation, validation, writing – reviewing and editing. **Jason Bonacci:** investigation, validation, writing – reviewing and editing. **Marise C. Breet:** investigation, validation, writing – reviewing and editing. **Simone Cranage:** investigation, validation, writing – reviewing and editing. **Shane V. Caswell:** investigation, validation, writing – reviewing and editing. **J.J. Hannigan:** investigation, validation, writing – reviewing and editing. **Alexis Herbaut:** investigation, validation, writing – reviewing and editing. **Karsten Hollander:** investigation, validation, writing – reviewing and editing. **Peter O. Ibikunle:** investigation, validation, writing – reviewing and editing. **Rachel A. Kennedy:** investigation, validation, writing – reviewing and editing. **Pui Wah Kong:** investigation, validation, writing – reviewing and editing. **Jayishni N. Maharaj:** investigation, validation, writing – reviewing and editing. **Natalie Mazzella:** investigation, validation, writing – reviewing and editing. **Shannon E. Munteanu:** investigation, validation, writing – reviewing and editing. **Liria A. Okai‐Nóbrega:** investigation, validation, writing – reviewing and editing. **Gabriel Gijon‐Nogueron:** investigation, validation, writing – reviewing and editing. **Jan Plesek:** investigation, validation, writing – reviewing and editing. **Hassan Saeedi:** investigation, validation, writing – reviewing and editing. **Jasper W.K. Tong:** investigation, validation, writing – reviewing and editing. **Abebayehu Tora:** investigation, validation, writing – reviewing and editing. **Ranel Venter:** investigation, validation, writing – reviewing and editing. **Yasin Yurt:** investigation, validation, writing – reviewing and editing. **Helen Banwell:** conceptualization, methodology, investigation, writing – original draft preparation, writing – reviewing and editing.

## Ethics Statement

Approval was given by the Human Research Ethics Committees of Monash University (43612).

## Consent

The authors have nothing to report.

## Conflicts of Interest

C.M.W. and S.C.M. is an Associate Editor and S.E.M. is a past Deputy Editor of the Journal of Foot and Ankle Research. It is journal policy that current editors are removed from the peer review and editorial decision‐making process for the papers that they have co‐authored. All other authors declare that they have no conflicts of interest.

## Permission to Reproduce Material From Other Sources

The authors have nothing to report.

## Supporting information

Supporting Information S1

Supporting Information S2

Supporting Information S3

Supporting Information S4

Supporting Information S5

Supporting Information S6

## Data Availability

All available data are provided within the manuscript.
