# Supplementary material for: Development of the CHILD‐SHOE Reporting Checklist: A Scoping Review and Modified Delphi Study to Support Reporting in Children's Footwear Research
Source: J Foot Ankle Res. 2025 Jul 9;18(3):e70065. doi: 10.1002/jfa2.70065 (PMC12241440; doi:10.1002/jfa2.70065)
Supplement: Supplementary file 1 — Supporting Information S1 [file JFA2-18-e70065-s005.docx]

Appendix 1.

**OVID Medline, EBM, Emcare and AMED (Allied and Complementary Medicine) search:**

1. Child. mp

2. Infant.mp

3. Adolescent.mp

4. p*ediatric.mp

5. 1 or 2 or 3 or 4

6. Shoe.mp

7. Shod.mp

8. Footwear.mp

9. 6 or 7 or 8

10. 5 and 9

**CINAHL**

1. Child OR Infant OR adolescents OR p#ediatric
2. Shoe OR Shod OR Footwear
3. S1 AND S2
