# Supplementary material for: Development of the CHILD‐SHOE Reporting Checklist: A Scoping Review and Modified Delphi Study to Support Reporting in Children's Footwear Research
Source: J Foot Ankle Res. 2025 Jul 9;18(3):e70065. doi: 10.1002/jfa2.70065 (PMC12241440; doi:10.1002/jfa2.70065)
Supplement: Supplementary file 2 — Supporting Information S2 [file JFA2-18-e70065-s003.docx]

| **Primary author** | **Geographic region** | **Participants (n)** | **Footwear data extracted** | **Outcome data extracted** |
| --- | --- | --- | --- | --- |
| Abolarin [1] | Africa | 50 | **Footwear features:**  Upper of shoe covers full or part of foot | **Foot features**  Arch Index (modified version) |
| Aboutorabi [2] | Asia | 30 | **Footwear description:**  Includes image of the footwear  **Footwear features:**  Therapeutic footwear (medical/orthopaedic)  General or casual shoe  Topline of shoe in relation to the ankle  Toe box of upper  Heel counter presence  Upper materials | **Balance and Gross motor function**  Centre of Pressure (CoP) displacement (mm)  **Spatiotemporal measures**  Step length (cm)  Step width (cm)  Velocity (m/sec)  **Foot features**  Foot posture index |
| Ashton [3] | Europe | 250 | **Footwear type:**  Sneakers, runners, trainers or sport/athletics shoes  Mary Jane, ballet flat or t-bar shoe  **Footwear Features:**  Fixtures (e.g.velcro, laces etc) of shoe  Upper material | **Skin/nail trauma linked to footwear use** |
| Au [4] | Africa | 12 | **Footwear type:**  Biomimetic, functional or minimalist  Runner  **Feature:**  Upper material  Mass (e.g., grams) of shoes  Pitch, drop and/or stack of outsole | **Kinematics and kinetics**  Knee joint angles  Ankle joint angles  Foot joint angles  Ground reaction force |
| Baker [5] | North America | 3015 | **Footwear type:**  Sandal, flip flops or slides  Boots  General or casual shoe  Slipper/indoor shoe  Sneakers, runners, trainers or sport/athletics shoes | **Skin/nail trauma** |
| Beyaert [6] | Europe | 12 | **Footwear description:**  Includes image of the footwear  **Footwear type:**  Boots  **Footwear Features:**  Pitch, drop and/or stack of outsole | **Kinematics and kinetics**  Knee joint angles  Ankle joint angles  Foot joint angles  Ground reaction force  Joint power  **Spatiotemporal measures**  Velocity (m/sec)  Swing phase %  Stride length (cm)  **EMG** |
| Bird [7] | Africa | 1056 | **Footwear type:**  Sneakers, runners, trainers or sport/athletics shoes  **Footwear Features:**  Upper material  Sole material | **Social factors**  Wear time  Footwear comfort  **Presence of infection** |
| Bleck [8] | North America | 1000 | **Footwear description:**  Includes image of the footwear  **Footwear type:**  General or casual shoe  Sneakers, runners, trainers or sport/athletics shoes  **Footwear features:**  Outsole with/without separate heel  Sole shape (including last) of shoe  Upper material | **Longitudinal impact of footwear on foot shape** |
| Breet [9] | Africa | 698 | **Footwear description:**  Includes image of the footwear  **Footwear type:**  School shoe (oxford style) | **Foot features**  Foot size |
| Buckland [10] | North America | 26 | **Footwear type:**  Sneakers, runners, trainers or sport/athletics shoes  **Footwear features:**  Fixtures (e.g.velcro, laces etc) of shoe | **Spatiotemporal measures**  Step length (cm)  Stance time (sec)  Step Width (cm)  Velocity (m/sec) |
| Burger [11] | Europe | 74 | **Footwear type:**  General or casual shoe | **Quality of life measure**  Oxford Ankle Foot Questionnaire  Peds QL  **Body image perception relating to feet look (VAS)**  **Foot features**  Hallux valgus angle  **Plantar pressure**  Mean pressure |
| Busscher [12] | Europe | 346 | **Footwear type:**  General or casual shoe | **Foot features**  Foot size  **Spatiotemporal measures**  Velocity (m/sec) |
| Byrne [13] | Europe | 50 | **Footwear type:**  General or casual shoe  **Footwear features:**  Toe box (shape and/or height) of upper  Pitch, drop and/or stack of outsole Length  Fixtures (e.g.velcro, laces etc) of shoe  Upper material  Sole materials | **Foot features**  Foot size |
| Chard [14] | Oceania | 13 | **Footwear type:**  Sandal, flip flops or slides  **Footwear features:**  Fixtures (e.g.velcro, laces etc) of shoe  Sole flexibility  Sole materials | **Kinematics and Kinetics**  Ankle angles  Foot angles  **Spatiotemporal measures**  Velocity (m/sec) |
| Chen [15] | Asia | 21 | **Footwear description:**  Includes image of the footwear  **Footwear type:**  Sneaker  **Footwear features:**  Mass (e.g., grams) of shoes  Fixtures (e.g.velcro, laces etc) of shoe  Sole shape | **Spatiotemporal measures**  Stride length (cm)  Velocity (m/sec)  Cadence (steps/sec)  Swing phase %  Stance phase %  **Kinetics and Kinematics**  Ground reaction force  **EMG** |
| Chen [16] | Asia | 10 | **Footwear description:**  Includes image of the footwear  **Footwear type:**  Sneakers, runners, trainers or sport/athletics shoes  **Footwear features:**  Sole flexibility | **Kinetics and Kinematics**  Impulse  Joint power  **Balance and Gross Motor Function**  Lower Limb Muscle Strength |
| Cranage [17] | Oceania | 47 | **Footwear description:**  Includes image of the footwear  **Footwear type:**  Sneaker  **Footwear features:**  Heel counter presence  Pitch, drop and/or stack of outsole  Fixture  Mass (e.g., grams) of shoes | **Spatiotemporal measures**  Velocity (cm/sec)  Cadence (steps/min)  Step time (sec)/length(cm)  Swing/stance phase (%)  Double support time (sec)  Toe in/out (degree)  Steps (count) |
| De Giorgio [18] | Europe | 34 | **Footwear description:**  Includes image of the footwear  **Footwear type:**  Soccer shoes  **Footwear features:**  Colour | **Physical Activity Measures**  Ball contact |
| Eek [19] | Europe | 10 | **Footwear description:**  Includes image of the footwear  **Footwear type:**  Sneakers, runners, trainers or sport/athletics shoes  **Footwear features:**  Upper of the shoe covers full or part of foot  Heel counter presence  Pitch, drop and/or stack of outsole  Fixtures (e.g.velcro, laces etc) of shoe | **Spatiotemporal measures**  Velocity (m/s)  Stride length (m)  Cadence (steps/min)  Stance Phase (%) |
| Hafez [20] | Africa | 8 | **Footwear features:**  Pitch, drop and/or stack of outsole  Tox box of upper | **Kinetics and Kinematics**  Knee joint angles  Ankle joint angles  Foot joint angles |
| Erdman [21] | North America | 3894 | **Footwear type:**  Sport specific (with sport listed) shoe | **Balance and Gross Motor Function**  Modified balance error scoring system (mBESS) |
| Erdman [22] | North America | 2667 | **Footwear type:**  Sport specific (with sport listed) shoe | **Balance and Gross Motor Function**  Modified Balance Error Scoring System (mBESS) |
| Erol Celik [23] | Asia | 136 | **Footwear type:**  Sport specific (with sport listed) shoe  **Footwear features:**  Toe box height  Fixtures (e.g.velcro, laces etc) of shoe | **Balance and Gross Motor Function**  Flamingo Balance Test  Functional Reach Test (FRT)  **Plantar Pressure**  % pressure at fore/hind foot  Mean pressure |
| Fong [24] | Asia | 12 | **Footwear description:**  Includes image of the footwear  **Footwear type:**  Sport specific (with sport listed) shoe  **Footwear features:**  Upper material | **Spatiotemporal**  Velocity m/s  **Kinematics and kinetics**  Vertical loading |
| Fon Yan [25] | Oceania | 16 | **Footwear description:**  Includes image of the footwear  **Footwear type:**  Sport specific  **Footwear features:**  Fixtures (e.g.velcro, laces etc) of shoe  Upper materials  Sole materials | **Kinematics**  Ankle angles  Toe in/out angles |
| Forrest [26] | North America | 10 | **Footwear description:**  Includes image of the footwear  **Footwear descriptions**  Brand name    **Footwear features:**  Sole materials  Insole materials | **Kinematics and kinetics**  Ground reaction force  Vertical Loading |
| Ganesh [27] | Asia | 100 | **Footwear features:**  Upper of shoe covers full or part of foot | **Foot features**  Arch index |
| Ganjehie [28] | Asia | 17 | **Footwear description:**  Includes image of the footwear  **Footwear type:**  General or casual | **Plantar pressure**  Centre of Pressure (CoP) displacement (mm)  Angle of gait (deg) |
| Ganjehie [29] | Asia | 17 | **Footwear description:**  Includes image of the footwear  **Footwear features:**  Fixtures (e.g.velcro, laces etc) of shoe  Heel counter  Upper of shoe covers full or part of foot  Pitch, drop and/or stack of outsole | **Plantar pressure**  Angle of gait (deg)  Centre of Pressure (CoP) displacement (mm) |
| Gdovin [30] | North America | 11 | **Footwear description:**  Includes image of the footwear  **Footwear descriptions**  Brand name  **Footwear features**  Mass (e.g., grams) of shoes | **Spatiotemporal**  Velocity (m/s)  Stride length (cm)  **Kinematics and Kinetics**  Hip angles  Knee angles  Ankle angles  **Plantar Pressure**  Angle of gait |
| Giacomini [31] | South America | 415 | **Footwear features:**  Pitch, drop and/or stack of outsole | **Plantar Pressure**  Angle of gait |
| Gimunova [32] | Europe | 30 | **Feature type:**  Biomimetic, functional or minimalist  **Footwear features:**  Sole flexible  Mass (e.g., grams) of shoes  Toe box | **Spatiotemporal**  Contact time (sec)  **Kinematics and kinetics**  Peak pressure (kPa)  Ground reaction force  **Plantar Pressure**  Angle of gait |
| Gonzalez Elena [33] | Europe | 505 | **Footwear description:**  Includes image of the footwear  **Footwear type:**  Outdoor Footwear  **Footwear features:**  Topline of shoe in relation to the ankle | **Foot features**  Foot size  Instep height |
| Gould [34] | North America | 79 | **Footwear features:**  Upper materials | **Spatiotemporal measures**  Cadence (steps/second)  Stance time (sec)  **Comfort**  Shoe fit measured with a device |
| Hassan [35] | Oceania | 33 | **Footwear description:**  Includes image of the footwear  **Footwear type:**  Brand  **Footwear description:**  General or casual shoe  **Footwear features:**  Fixtures (e.g.velcro, laces etc) of shoe | **Spatiotemporal measures**  Velocity (m/s)  Cadence (steps/sec)  Stride length (cm)  Step length (cm)  Base of support (cm)  Toe-in/toe out angles  Step width |
| Heidner [36] | South America | 69 | **Footwear description:**  Brand  **Footwear features:**  Upper materials  Pitch, drop and/or stack of outsole  Upper of shoe covers full or part of foot | **Kinetics and Kinematics**  Ground reaction force  Vertical loading |
| Heidner [37] | South America | 85 | **Footwear description:**  Brand  **Footwear features:**  Pitch, drop and/or stack of outsole  Upper of shoe covers full or part of foot | **Spatiotemporal measures**  Contact time  Stance time  **Kinematics and Kinetics**  Ground reaction force  Vertical loading |
| Herbaut [38] | Europe | 13 | **Footwear description:**  Includes image of the footwear  **Footwear type**  Sport specific (with sport listed) shoe  **Footwear description:**  Includes image of the footwear  **Footwear features**  Insole materials  Outer materials  Fixtures (e.g.velcro, laces etc) of shoe  Heel counter  Upper of shoe covers full or part of foot  Pitch, drop and/or stack of outsole | **Spatiotemporal measures**  Step length (cm)  Velocity (m/s)  **Kinematics and Kinetics**  Knee joint angles  Ankle joint angles  Peak impact force |
| Herbaut [39] | Europe | 30 | **Footwear description:**  Includes image of the footwear  **Footwear type**  Sport shoes (with sport listed) shoe    **Footwear features**  Insole materials  Outer materials  Fixtures (e.g.velcro, laces etc) of shoe  Heel counter | **Kinematics and Kinetics**  Knee joint angles  Ankle joint angles  Ground reaction force |
| Herbaut [40] | Europe | 14 | **Footwear description:**  Includes image of the footwear  **Footwear type**  Sport shoes (with sport listed) shoe    **Footwear features**  Insole materials  Outer materials  Fixtures (e.g.velcro, laces etc) of shoe  Heel counter  Pitch, drop and/or stack of outsole | **Spatiotemporal**  Stance time (sec)  Velocity (m/s)  **Kinematics and Kinetics**  Knee joint angles  Ankle joint angles |
| Herbaut [41] | Europe | 16 | **Footwear description:**  Includes image of the footwear  **Footwear type**  Sport specific (with sport listed) shoe  **Footwear features**  Upper materials | **Pressure distribution**  Mean pressures (kPa) |
| Hillis [42] | Europe | 150 | **Footwear type**  School shoe  **Footwear features**  Topline of shoe in relation to the ankle  Heel counter | **Foot features**  Foot size  Skin/nail trauma  **Comfort**  Perception of shoe fit  **Body perception relating to feet look** |
| Hillstrom [43] | North America | 25 | **Footwear description:**  Includes image of the footwear  **Footwear description**  Brand  **Footwear Type**  Minimalistic  **Footwear features**  Outsole without heel  Sole flexibility | **Foot Features**  Ankle range of motion  **Kinematics and Kinetics**  Peak pressure (kPa) |
| Hodgson [44] | Europe | 239 | **Footwear Type**  Sneakers, runners, trainers or sport/athletics shoes | **Social factors**  Patterns of ownership |
| Hollander [45] | Europe | 36 | **Footwear description**  Brand  Footwear description  Outsole materials  Mass (e.g., grams) of shoes  Pitch | **Spatiotemporal**  Step length (cm)  Step width (cm)  Cadence (steps per minute)  **Kinematics and Kinetics**  Ankle joint angles  Knee joint angles |
| Hollander [46] | Europe | 678 | **Footwear description:**  Includes image of the footwear  **Footwear type:**  Sport shoe (with sport listed) shoe | **Kinematics and Kinetics**  Vertical loading |
| Hollman [47] | Europe |  | **Footwear features**  Toe box (shape and/or height) of upper  Sole shape  Fixtures (e.g.velcro, laces etc) of shoe  Outer material  Upper material  Topline of shoe in relation to the ankle (eg high, mid or low cut) |  |
| Ibikunle [48] | Africa | 352 | **Footwear features**  Upper of shoe covers full or part of foot  Upper material  **Footwear type:**  Sandal, flip flops or slides  Slipper/indoor shoe | **Foot features**  Arch index |
| Ijzerman [49] | Europe | 12 |  | **Physical Activity Measures**  Energy expenditure |
| Imalele [50] | Africa | 504 |  | **Social factors**  Wear time  School attendance |
| James [51] | Oceania | 124 | **Brand name**  **Footwear type:**  School shoes  Runners  Casual or general  **Footwear features**  Heel counter presence and/or it’s stiffness  Outer materials | **Foot features**  Foot posture index  Ankle range of motion  **Physical activity measures**  Physical activity diary  **Quality of life measures**  Oxford Ankle Foot Questionnaire  **Comfort**  Pain (VAS) |
| Kaniwa [52] | North America | 5 | **Footwear types**  Sandal  **Footwear features**  Upper composition  Insole material | **Foot features**  Skin/nail trauma |
| Kennedy [53] | Oceania | 60 | **Footwear description:**  Includes image of the footwear  **Footwear type**  Sneakers, runners, trainers or sport/athletics shoes  School shoe (oxford style)  **Footwear features**  Fixtures (e.g.velcro, laces etc) of shoe | **Comfort**  Footwear fit  **Spatiotemporal measures**  Velocity  Cadence  Step length (cm)  Step time (sec)  Base of support width  Single/Double support time % |
| Kennedy [54] | Oceania | 143 | **Footwear type**  Athletic shoes | **Spatiotemporal measures**  Velocity  Cadence  Step length (cm)  Stride length (cm)  Single/Double support time %  Step width (cm)  Step count |
| Kim [55] | Oceania | 10 | **Footwear description:**  Includes image of the footwear  **Footwear description**  Brand  **Footwear features**  Pitch, drop and/or stack of outsole  Sole material  Upper material Glue and/or adhesives  Minimalist index | **Spatiotemporal measures**  Cadence (steps/sec)  Stance time (sec)  Step length (cm)  Velocity (m/s)  Stance time  Swing time |
| Kinsella [56] | Europe | 1 | **Footwear description**  Brand  **Footwear features**  Biomimetic, functional or minimalist  Mass (e.g., grams) of shoes | **Spatiotemporal measures**  Velocity (m/s)  Cadence  Step length  Stride length  Swing  Jump height (cm)  **Kinetics and kinematics**  Joint power  Ground Reaction Force (N) |
| Kinz [57] | Asia | 620 | **Footwear features**  Indoor shoes  Insole materials | **Foot features**  Foot size  Hallux valgus angle  **Comfort**  Shoe fit measured with a fit device |
| Klein [58] | Oceania | 858 | **Footwear features**  Indoor shoes  Heel counter presence  Upper of shoe covers full or part of foot  Pitch, drop and/or stack of outsole  Outsole materials  Fixtures (e.g.velcro, laces etc) of shoe | **Foot features**  Hallux valgus angle |
| Kung [59] | Oceania | 13 | **Footwear description**  Brand  **Footwear features**  Upper materials  Insole materials  Mass (e.g., grams) of shoes | **Spatiotemporal measures**  Velocity (m/s)  **Kinematics and kinetics**  Joint power  Impulse (NM/s)  Hip angles  Knee angles  Ankle angles  Foot angles  Ground Reaction Force |
| Latorre Roman [60] | Europe | 713 | **Footwear features**  Running Shoes | **Spatiotemporal measures**  Velocity (m/s)  Swing phase  Stance phase |
| Lopez [61] | Europe | 12 | **Footwear description**  Brand  **Composition or materials of the footwear**  Upper material  Sole material  Glue and/or adhesives | **Spatiotemporal measures**  Stance phase  Swing phase  Double/single support  Velocity (m/s)  **Kinematic and kinetics**  Hip angles  Knee angles  Ankle angles |
| Lythgo [62] | Oceania | 980 | **Footwear features**  Athletic shoes/runners | **Spatiotemporal measures**  Velocity (m/s)  Cadence (steps/sec)  Step length  Stride length  Single/double support  Stance time |
| Maharaj [63] | Oceania | 8 | **Footwear features**  Flip Flops | **Kinematics and Kinetics**  Hip angles  Knee angles  Ankle angles  Joint power (W/kg)  Impulse |
| Martin - Casado [64] | South America | 1662 | **Footwear features**  School shoes  Upper of shoe covers full or part of foot | **Foot Features**  Foot size  Heel to metatarsal  Instep height  Hallux valgus angle |
| Matthias [65] | Oceania | 14 | **Footwear description**  Brand name  **Footwear features**  School Shoe  Upper materials  Fixtures (e.g.velcro, laces etc) of shoe  Upper of shoe covers full or part of foot  Enclosed Heel counter presence and/or it’s stiffness Pitch, drop and/or stack of outsole | **Spatiotemporal measures**  Velocity (m/s)  **Kinematics and kinetics**  Ankle angles  Foot angles  **Comfort**  Footwear comfort |
| Mazzella [66] | Oceania | 24 | **Footwear description**  Brand  **Footwear features**  Mass (e.g., grams) of shoes  Heel counter presence and/or it’s stiffness Sole flexibility | **Comfort**  Pain (Binary/Visual Analogue Scale) |
| Mazzella [67] | Oceania | 52 | **Footwear description**  Brand  **Footwear features**  Athletic shoe  Pitch, drop and/or stack of outsole  Mass (e.g., grams) of shoes | **Kinematics and Kinetics**  Hip angles  Knee angles  Ankle angles |
| Mazzella [68] | Oceania | 28 | **Footwear description**  Brand  **Footwear features**  Biomimetic, functional or minimalist  Pitch, drop and/or stack of outsole  Sole flexibility  Mass (e.g., grams) of shoes | **Kinematics and kinetics**  Vertical loading  Ground reaction force |
| Medina-Alcantara [69] | Europe | 132 | **Footwear features**  Boot  Topline of shoe in relation to the ankle  Outsole with/without separate heel | **Foot features**  Arch index |
| Mullen [70] | North America | 12 | **Footwear features**  Trainer  Mass (e.g., grams) of shoes | **Spatiotemporal measures**  Stride length  Stance phase %  Swing phase %  Velocity (m/s)  Contact time |
| Munuera [71] | Europe | 48 | **Footwear features**  Biomimetic, functional or minimalist  Topline of shoe in relation to the ankle  Fixtures (e.g.velcro, laces etc) of shoe  Outsole materials | **Plantar Pressure**  Angle of Gait |
| Mwai [72] | Africa | 538 |  | **Comfort**  Wear time  **Presence of infection** |
| Mwangi [73] | Africa | 508 | **Footwear features**  Heel counter presence and/or it’s stiffness | **Presence of infection**  **Social factors**  Wear time |
| Oeffinger [74] | North America | 14 | **Footwear features**  Athletic shoe  Topline of shoe in relation to the ankle  Insole materials  Sole materials | **Kinematic and kinetics**  Hip angles  Knee angles  Ankle angles  Foot angles |
| Okai-Nobrega [75] | South America | 4 | **Footwear features**  Biomimetic  Insole materials | **Kinematics**  Hip angles  Knee angles  Ankle angles |
| Okai-Nobrega [76] | South America | 19 | **Footwear features**  Insole materials  Fixtures (e.g.velcro, laces etc) of shoe | **Spatiotemporal**  Velocity (m/s)  Step length  Stride length  Step Width |
| O'Kane [77] | North America | 351 | **Footwear features**  Cleat soccer shoes  cleats on soles of footwear | **Comfort**  Number of injuries |
| Onder [78] | Asia | 1 | **Footwear features**  Glue and/or Adhesive  Upper materials |  |
| Pizac [79] | North America | 23 | **Footwear description**  Brand  **Footwear features**  Sport specific (with sport listed) shoe  Sole materials  Mass (e.g., grams) of shoes  Topline of shoe in relation to the ankle | **Foot features**  Ankle range of movement |
| Plesek [80] | Europe | 48 | **Footwear features**  Running shoes  Insole materials  Pitch, drop and/or stack of outsole | **Kinematics and kinetics**  Ankle angles  **Plantar Pressure**  % pressure forefoot/hindfoot |
| Plesek [81] | Europe | 48 | **Footwear features**  Minimalist  Pitch, drop and/or stack of outsole | **Kinematics and kinetics**  Ankle angles  Knee angles  Hip angles  **Spatiotemporal measures**  Velocity (m/s)  **Plantar Pressure**  % pressure forefoot/hindfoot |
| Plesek [82] | Europe | 48 | **Footwear description**  Brand  **Footwear features**  Running shoes  Minimalist index of footwear  Mass (e.g., grams) of shoes  Pitch, drop and/or stack of outsole  Outsole with separate heel | **Spatiotemporal measures**  Stace phase %  **Kinematics and kinetics**  Ground reaction force  Vertical loading |
| Puszczalowska-Lizis [83] | Europe | 100 | **Footwear features**  Indoor shoes  Upper materials  Fixtures (e.g.velcro, laces etc) of shoe  Upper of shoe covers full or part of foot  Sole materials | **Foot features**  Foot size  Clarke’s angle  Ankle range of motion  Hallux Valgus angle |
| Puszczalowska-Lizis [84] | Europe | 100 | **Footwear features**  Indoor shoe  Upper materials  Fixtures (e.g.velcro, laces etc) of shoe  Upper of shoe covers full or part of foot  Heel counter presence and/or it’s stiffness  Sole materials | **Foot features**  Foot size Clarke’s angle  Wejsflog index  Ankle range of motion  Hallux valgus angle |
| Puszczalowska-Lizis [85] | Europe | 100 | **Footwear features**  Sneakers, runners, trainers or sport/athletics shoes  Fixtures (e.g.velcro, laces etc) of shoe  Sole materials  Upper materials | **Foot features**  Foot size  Clarke’s angle  Weisflogg index  Ankle range of motion  Hallux valgus angle  5^th^ toe degree |
| Puszczalowska-Lizis [86] | Europe | 100 | **Footwear description**  Footwear retail cost  **Footwear features**  Upper materials  Heel counter presence and/or it’s stiffness  Upper of shoe covers full or part of foot  Sole materials  Fixtures (e.g.velcro, laces etc) of shoe | **Foot features** |
| Quinlan [87] | Oceania | 70 | **Footwear features**  School shoes  Sole materials  Upper materials  Fixtures (e.g.velcro, laces etc) of shoe | **Foot features**  Muscle size |
| Rao [88] | Asia | 2300 | **Footwear features**  Shoes  Upper of shoe covers full or part of foot | **Foot features**  Foot size |
| Robinson [89] | North America | 12 | **Footwear description**  Brand name  **Footwear features**  Athletic shoe | **Balance and Gross Motor Function**  Test of Gross Motor Development |
| Schuttelaar [90] | Europe | 1 | **Footwear description**  Brand name  **Footwear features**  Sneakers, runners, trainers or sport/athletics shoes    **Composition or materials of footwear**  Glue and/or adhesives  Upper materials |  |
| Senden [91] | Europe | 55 | **Footwear features**  Sneakers, runners, trainers or sport/athletics shoes | **Spatiotemporal measures**  Step length  Step time  **Kinematics and kinetics**  Hip angles  Knee angles  Ankle angles  Ground Reaction Force  Joint power |
| Shields [92, 93] | Oceania | 50 |  | **Physical activity measures**  Steps per day  **Foot features**  Arch index  Hallux valgus angle  Foot size  **Comfort**  Shoe fit measured with a fit device  Wear time |
| Shultz [94] | North America | 25 | **Footwear features**  Running shoes  Pitch, drop and/or stack of outsole  Sole flexibility  Insole materials | **Physical activity measures**  VO2  Energy expenditure (kcal.min-1)  **Balance and Gross Motor Function**  Centre of Pressure (CoP) displacement (cm) |
| Smiley [95] | North America | 14 | **Footwear features**  Sport specific (with sport listed) shoe | **Spatiotemporal measures**  Velocity  Cadence  Stride length  **Kinematics and kinetics**  Hip angles  Knee angles  Ankle angles |
| Staheli [96] | North America | 190 | **Footwear description**  Footwear retail cost  **Footwear features**  Athletic shoe  **Footwear features**  General shoes | **Social factors**  Recommendations from health professionals |
| Tan [97] | Asia | 23 | **Footwear features**  Covered | **Balance and Gross Motor Function**  Single leg stance  Tip toe talking |
| Taylor [98] | North America | 20 | **Footwear description**  Brand  **Footwear features**  Sport specific (with sport listed) shoe  Sole materials  Pitch, drop and/or stack of outsole  Sole flexibility | **Plantar pressure**  Mean pressure |
| Teixeira [99] | Europe | 1 | **Footwear features**  Upper material  Sole material  Glue and/or adhesives | **Foot features**  Skin and nail trauma |
| Tinker [100] | Oceania | 38 | **Footwear features**  Sneakers, runners, trainers or sport/athletics shoe | **Spatiotemporal measures**  Step length  Cadence  **Kinematics and kinetics**  Hip angles  Knee angles  Ankle angles  Vertical loading  Joint power  Ground reaction force |
| Tong [101] | Asia | 111 | **Footwear features**  Sandal, flip flops or slides  Fixtures (e.g.velcro, laces etc) of shoe  Upper of shoe covers full or part of foot | **Plantar pressure**  Mean pressure  % pressure of fore/hind foot  **Foot features**  Arch index  Instep height  Foot size |
| Tora [102] | Africa | 330 | **Footwear features**  Sneakers, runners, trainers or sport/athletics shoes  Upper of shoe covers full or part of foot  Upper material | **Social factors**  Wear time  Patterns of ownership |
| Traut [103] | North America | 14 | **Footwear description**  Brand name  **Footwear features**  Biomimetic, functional or minimalist  Pitch, drop and/or stack of outsole | **Kinematics and kinetics**  Vertical loading  Hip angles  Knee angles  Ankle angles |
| Trevisan [104] | Europe | 9 | **Footwear features**  Upper material |  |
| Walter [105] | North America | 36 | **Footwear features**  Sport specific (with sport listed) shoe  Insole materials in shoe  Heel counter presence and/or it’s stiffness  Upper of shoe covers full or part of foot  Fixtures (eg velcro, laces etc) of shoe | **Spatiotemporal measures**  Contact time  **Plantar pressure**  Mean pressure |
| Watanabe [106] | Africa | 168 | **Footwear features**  Upper of shoe covers full or part of foot | **Social factors**  Wearing behaviours  Pattern of ownership  **Skin and nail trauma linked to footwear usage** |
| Wegener [107] | Oceania | 20 | **Footwear description**  Brand name  **Footwear features**  Upper material  Outer materials  Fixtures (eg velcro, laces etc) of shoe  Insole materials in shoe  Heel counter presence and/or it’s stiffness  Pitch, drop and/or stack of outsole | **Spatiotemporal measures**  Velocity  Contact time  Stride length  **Kinematics and kinetics**  Ankle angle  Foot angle  **Foot features**  Foot Posture Index |
| Williams [108] | Oceania | 14 | **Footwear description**  Brand name  **Footwear type**  Pre-walker  **Footwear features**  Upper material  Fixtures (eg velcro, laces etc) of shoe  Heel counter presence and/or it’s stiffness | **Spatiotemporal measures**  Velocity (cm/sec)  Cadence (Steps/second)  Stride time (sec)  Stride length (cm)  Step time (sec)  Step length (cm)  Swing phase (%)  Stance phase (%)  Double/single support  Toe in/ toe out angles (degrees)  **Kinematics and kinetics**  Hip angles  Knee angles  Ankle angles |
| Wolf [109] | Europe | 18 | **Footwear descriptions**  Commercial availability  **Footwear features**  Fixtures (eg velcro, laces etc) of shoe  Sole flexibility | **Kinematics and kinetics**  Hip angles  Knee angles  Ankle angles  Foot angles  Joint power |
| Yurt [110] | Asia | 1000 | **Footwear features**  Boot  School shoe (oxford style)  Upper materials | **Comfort**  Footwear assessment score  Shoe fit measured with a fit device |

Reference list for table:

1. Abolarin T, Aiyegbusi A, Tella A, Akinbo S: **Predictive factors for flatfoot: The role of age and footwear in children in urban and rural communities in South West Nigeria.** *Foot* 2011, **21:**188-192.

2. Aboutorabi A, Saeedi H, Kamali M, Farahmand B, Eshraghi A, Dolagh RS: **Immediate effect of orthopedic shoe and functional foot orthosis on center of pressure displacement and gait parameters in juvenile flexible flat foot.** *Prosthetics & Orthotics International* 2014, **38:**218-223.

3. Ashton RE, Griffiths WA: **Juvenile Plantar Dermatosis--atopy or footwear?** *Clinical & Experimental Dermatology* 1986, **11:**529-534.

4. Au IPH, Lau FOY, An WW, Zhang JH, Chen TL, Cheung RTH: **Immediate and short-term biomechanical adaptation of habitual barefoot runners who start shod running.** *Journal of Sports Sciences* 2018, **36:**451-455.

5. Baker MD, Bell RE: **The role of footwear in childhood injuries.** *Pediatric Emergency Care* 1991, **7:**353-355.

6. Beyaert C, Pierret J, Vasa R, Paysant J, Caudron S: **Toe walking in children with cerebral palsy: a possible functional role for the plantar flexors.** *Journal of Neurophysiology* 2020, **124:**1257-1269.

7. Bird C, Ame S, Albonico M, Bickle Q: **Do shoes reduce hookworm infection in school-aged children on Pemba Island, Zanzibar? A pragmatic trial.** *Transactions of the Royal Society of Tropical Medicine & Hygiene* 2014, **108:**297-304.

8. Bleck EE: **The shoeing of children: sham or science?** *Developmental Medicine & Child Neurology* 1971, **13:**188-195.

9. Breet MC, Venter R: **Are habitually barefoot children compelled to wear ill-fitting school shoes? A cross-sectional study.** *BMC Pediatrics* 2022, **22:**187.

10. Buckland MA, Slevin CM, Hafer JF, Choate C, Kraszewski AP: **The Effect of Torsional Shoe Flexibility on Gait and Stability in Children Learning to Walk.** *Pediatric Physical Therapy* 2014, **26:**411-417.

11. Burger EB, Lale SA, Hovius SER, Nieuwenhoven CAV, Bus SA: **Foot Function in Patients With Surgically Treated Preaxial Polydactyly of the Foot Compared With Age- and Sex-Matched Healthy Controls.** *Foot & Ankle International* 2019, **40:**414-421.

12. Busscher I, Wapstra F, Bulstra S, Veldhuizen A, Aubin C, Stokes IAF, Labelle H, Moreau A: **The value of shoe size for predicting the timing of the pubertal growth spurt.** *Studies in Health Technology & Informatics* 2010, **158:**259-259.

13. Byrne M, Curran MJ: **The development and use of a footwear assessment score in comparing the fit of children's shoes.** *Foot* 1998, **8:**215-218.

14. Chard A, Greene A, Hunt A, Vanwanseele B, Smith R: **Effect of thong style flip-flops on children’s barefoot walking and jogging kinematics.** *Journal of foot and ankle research* 2013, **6:**8-8.

15. Chen JP, Chung MJ, Wu CY, Cheng KW, Wang MJ: **Comparison of Barefoot Walking and Shod Walking Between Children with and Without Flat Feet.** *Journal of the American Podiatric Medical Association* 2015, **105:**218-225.

16. Chen H, Shao E, Sun D, Xuan R, Baker JS, Gu Y: **Effects of footwear with different longitudinal bending stiffness on biomechanical characteristics and muscular mechanics of lower limbs in adolescent runners.** *Frontiers in physiology* 2022, **13:**907016.

17. Cranage S, Perraton L, Bowles KA, Williams C: **A comparison of young children's spatiotemporal measures of walking and running in three common types of footwear compared to bare feet.** *Gait & Posture* 2020, **81:**218-224.

18. De Giorgio A, Sellami M, Kuvacic G, Lawrence G, Padulo J, Mingardi M, Mainolfi L: **Enhancing motor learning of young soccer players through preventing an internal focus of attention: The effect of shoes colour.** *PLoS ONE [Electronic Resource]* 2018, **13:**e0200689.

19. Eek MN, Zugner R, Stefansdottir I, Tranberg R: **Kinematic gait pattern in children with cerebral palsy and leg length discrepancy: Effects of an extra sole.** *Gait & Posture* 2017, **55:**150-156.

20. Ehab H: **Effect of rounded bottom profile shoes on foot clearance in children with stiff knee gait.** *Gait & Posture* 2017, **57:**239-240.

21. Erdman NK, Kelshaw PM, Hacherl SL, Cortes N, Caswell SV: **Testing Surface And Footwear Type Significantly Affect Baseline Balance Performance Of Middle School Athletes...2021 ACSM Annual Meeting & World Congresses [Virtual], June 1-5, 2021.** *Medicine & Science in Sports & Exercise* 2021, **53:**172-172.

22. Erdman NK, Kelshaw PM, Hacherl SL, Caswell SV: **Footwear type and testing environment do not affect baseline modified balance error scoring system performance among middle school athletes.** *Journal of Sport Rehabilitation* 2023, **32:**9-13.

23. Erol ÇElİK S, Bek N, ÖZcebe LH, Kocaman H: **THE EFFECTS OF SHOE SUITABILITY ON PLANTAR PRESSURE DISTRIBUTION AND BALANCE PARAMETERS IN CHILDREN WITH HEARING IMPAIRMENT.** *Turkish Journal of Physiotherapy Rehabilitation* 2022, **33:**54-62.

24. Fong DT, Hong Y, Li JX: **Cushioning and lateral stability functions of cloth sport shoes.** *Sports Biomechanics* 2007, **6:**407-417.

25. Fong Yan A, Smith R, Vanwanseele B, Hiller C: **Mechanics of jazz shoes and their effect on pointing in child dancers.** *Journal of Applied Biomechanics* 2012, **28:**242-248.

26. Forrest D, Dufek JS, Mercer JA: **Impact characteristics of female children running in adult versus youth shoes of the same size.** *Journal of Applied Biomechanics* 2012, **28:**593-598.

27. Ganesh MSP, Magnani B: **The Influence of Footwear on the Prevalence of Flat Foot.** *Indian Journal of Physiotherapy & Occupational Therapy* 2016, **10:**157-159.

28. Ganjehie S, Saeedi H, Farahmand B, Curran S: **The efficiency of gait plate insole for children with in-toeing gait due to femoral antetorsion.** *Prosthetics and orthotics international* 2016, **41:**51-57.

29. Ganjehie S, Saeedi H, Farahmand B, Curran S: **The efficiency of gait plate insole for children with in-toeing gait due to femoral antetorsion.** *Prosthetics & Orthotics International* 2017, **41:**51-57.

30. Gdovin JR, Wade C, Williams CC, Luginsland LA, Wilson SJ, Garner JC: **Impact of shoe and cleat type on youth baseball pitching biomechanics.** *Sports Biomechanics* 2022, **21:**761-772.

31. Giacomini BA, Yamato TP, Lopes AD, Hespanhol L: **What is the foot strike pattern distribution in children and adolescents during running? A cross-sectional study.** *Brazilian journal of physical therapy* 2020, **25:**336-343.

32. Gimunova M, Kolarova K, Vodicka T, Bozdech M, Zvonar M: **How barefoot and conventional shoes affect the foot and gait characteristics in toddlers.** *PLoS ONE* 2022, **17:**e0273388.

33. Gonzalez Elena ML, Cordoba-Fernandez A: **Footwear fit in schoolchildren of southern Spain: a population study.** *BMC Musculoskeletal Disorders* 2019, **20:**208.

34. Gould N: **Shoes versus sneakers in toddler ambulation.** *Foot & Ankle* 1985, **6:**105-107.

35. Hassan NM, Shields N, Landorf KB, Buldt AK, Taylor NF, Evans A, Williams C, Menz HB, Munteanu SE: **Efficacy of custom-fitted footwear to increase physical activity in children and adolescents with Down syndrome (ShoeFIT): randomised pilot study.** *Disability and rehabilitation* 2019, **43:**2131-2140.

36. Heidner GS, Nascimento RB, Aires AG, Baptista RR: **Barefoot walking changed relative timing during the support phase but not ground reaction forces in children when compared to different footwear conditions.** *Gait & Posture* 2020, **83:**287-293.

37. Heidner GS, Nascimento RB, Aires AG, Baptista RR: **Barefoot walking changed relative timing during the support phase but not ground reaction forces in children when compared to different footwear conditions.** *Gait & Posture* 2021, **83:**287-293.

38. Herbaut A, Chavet P, Roux M, Gueguen N, Gillet C, Barbier F, Simoneau-Buessinger E: **The influence of shoe drop on the kinematics and kinetics of children tennis players.** *European Journal of Sport Science EJSS : Official Journal of the European College of Sport Science* 2016, **16:**1121-1129.

39. Herbaut A, Simoneau-Buessinger E, Barbier F, Gillet C, Roux M, Gueguen N, Chavet P: **Shoe drop reduction influences the lower limb biomechanics of children tennis players during an open stance forehand: A longitudinal study.** *European Journal of Sport Science EJSS : Official Journal of the European College of Sport Science* 2017, **17:**1261-1269.

40. Herbaut A, Chavet P, Roux M, Gueguen N, Barbier F, Simoneau-Buessinger E: **The influence of shoe aging on children running biomechanics.** *Gait & Posture* 2017, **56:**123-128.

41. Herbaut A, Roux M, Gueguen N, Chavet P, Barbier F, Simoneau-Buessinger E: **Determination of optimal shoe fitting for children tennis players: Effects of inner-shoe volume and upper stiffness.** *Applied Ergonomics* 2019, **80:**265-271.

42. Hillis MA: **The shoes of school children.** *Medical Officer* 1949, **82:**37.

43. Hillstrom HJ, Buckland MA, Slevin CM, Hafer JF, Root LM, Backus SI, Kraszewski AP, Whitney KA, Scher DM, Song J, et al: **Effect of shoe flexibility on plantar loading in children learning to walk.** *Journal of the American Podiatric Medical Association* 2013, **103:**297-305.

44. Hodgson L, Hodges M, Williams AE, Nester CJ, Morrison SC: **The "price-tag" of foot health in infancy and early childhood: a cross sectional survey of UK parents.** *European Journal of Pediatrics* 2021, **180:**1561-1570.

45. Hollander K, Riebe D, Campe S, Braumann KM, Zech A: **Effects of footwear on treadmill running biomechanics in preadolescent children.** *Gait & Posture* 2014, **40:**381-385.

46. Hollander K, de Villiers JE, Venter R, Sehner S, Wegscheider K, Braumann KM, Zech A: **Foot Strike Patterns Differ Between Children and Adolescents Growing up Barefoot vs. Shod.** *International Journal of Sports Medicine* 2018, **39:**97-103.

47. Hollman C: **Shoes for children: a survey of retail shoeshops in the Borough of Ealing.** *British Medical Journal* 1960, **1:**719-722.

48. Ibikunle PO, Ikekwen EC: **Prevalence of Pes Planus and Its Associated Factors Among Primary School Pupils Aged 8-12 Years in Southeast Nigeria.** *Nigerian Journal of Medical Rehabilitation* 2017, **19:**NA-NA.

49. Ijzerman MJ, Nene AV: **Feasibility of the physiological cost index as an outcome measure for the assessment of energy expenditure during walking.** *Archives of Physical Medicine & Rehabilitation* 2002, **83:**1777-1782.

50. Imalele EE, Braide EI, Emanghe UE, Effanga EO, Usang AU: **Soil-transmitted helminth infection among school-age children in Ogoja, Nigeria: implication for control.** *Parasitology research* 2023, **122:**1015-1026.

51. James AM, Williams CM, Haines TP: **Effectiveness of footwear and foot orthoses for calcaneal apophysitis: a 12-month factorial randomised trial.** *British Journal of Sports Medicine* 2016, **50:**1268-1275.

52. Kaniwa MA, Isama K, Nakamura A, Kantoh H, Itoh M, Miyoshi K, Saito S, Shono M: **Identification of causative chemicals of allergic contact dermatitis using a combination of patch testing in patients and chemical analysis. Application to cases from rubber footwear.** *Contact Dermatitis* 1994, **30:**26-34.

53. Kennedy RA, McGinley JL, Paterson KL, Ryan MM, Carroll K: **Gait and footwear in children and adolescents with Charcot-Marie-Tooth disease: A cross-sectional, case-controlled study.** *Gait & Posture* 2018, **62:**262-267.

54. Kennedy RA, de Valle K, Adams J, Ryan MM, Fitzgerald AK, Carroll K: **Characterising gait in paediatric neuromuscular disorders: an observational study of spatio-temporal gait in a clinical cohort.** *Disability and rehabilitation* 2021, **44:**1-7.

55. Kim J, McSweeney SC, Hollander K, Horstman T, Wearing SC: **Adolescents running in conventional running shoes have lower vertical instantaneous loading rates but greater asymmetry than running barefoot or in partial-minimal shoes.** *Journal of Sports Sciences* 2023, **41:**774-787.

56. Kinsella S, Byrne PPJ, Bradley-O'Connor N, Coffey C, Callanan D, Frances P: **Effects Of Footwear On Fundamental Movement Skills In A Child With Autism Spectrum Disorder...2021 ACSM Annual Meeting & World Congresses [Virtual], June 1-5, 2021.** *Medicine & Science in Sports & Exercise* 2021, **53:**175-175.

57. Kinz W, Groll-Knapp E, Kundi M: **Hallux valgus in pre-school-aged children: the effects of too-short shoes on the hallux angle and the effects of going barefoot on podiatric health.** *Footwear Science* 2020, **13:**29-42.

58. Klein C, Groll-Knapp E, Kundi M, Kinz W: **Increased hallux angle in children and its association with insufficient length of footwear: a community based cross-sectional study.** *BMC Musculoskeletal Disorders* 2009, **10:**159.

59. Kung SM, Fink PW, Hume PA, Shultz SP: **Kinematic and kinetic differences between barefoot and shod walking in children.** *Footwear Science* 2015, **7:**95-105.

60. Latorre Roman PA, Balboa FR, Pinillos FG: **Foot strike pattern in children during shod-unshod running.** *Gait & Posture* 2017, **58:**220-222.

61. Lopez NR, Gomez RM, Valderrama MM, Gonzalez AG, de la Torre-Montero JC, Moreno AP-S, Fidalgo-Herrera AJ, Ribeiro ASF, Lopez-Moreno C, Martinez-Beltran MJ: **Biomechanical analysis of barefoot walking and three different sports footwear in children aged between 4 and 6 years old.** *PLoS ONE* 2023, **18:**e0291056.

62. Lythgo N, Wilson C, Galea M: **Basic gait and symmetry measures for primary school-aged children and young adults whilst walking barefoot and with shoes.** *Gait & Posture* 2009, **30:**502-506.

63. Maharaj JN, Barber L, Walsh HPJ, Carty CP: **Flip-flops do not alter the neuromuscular function of the gastrocnemius muscle and tendon during walking in children.** *Gait & Posture* 2020, **77:**83-88.

64. Martin-Casado L, Barquín C, Aldana-Caballero A, Marcos-Tejedor F, Aguado X: **Environmental Factors as a Cause of Differences in the Feet of Ecuadorian Children and Its Relation to Their Footwear.** *Children* 2021, **8:**1-8.

65. Matthias E, Banwell HA, Arnold JB: **Children's school footwear: The impact of fit on foot function, comfort and jump performance in children aged 8 to 12 years.** *Gait & Posture* 2021, **87:**87-94.

66. Mazzella N, Fox A, Trowell D, Saunders N, Vicenzino B, Bonacci J: **Flat flexible school shoes for adolescents with patellofemoral pain: a randomised, assessor-blinded, parallel-group feasibility trial.** *BMJ Open Sport and Exercise Medicine* 2023, **9:**e001717.

67. Mazzella N, Trowell D, Fox A, Saunders N, Vicenzino B, Bonacci J: **Gait biomechanics do not differ between adolescents with and without patellofemoral pain.** *Scandinavian Journal of Medicine and Science in Sports* 2024, **34:**e14587.

68. Mazzella N, Trowell D, Fox A, Saunders N, Vicenzino B, Bonacci J: **The Immediate Biomechanical Effects of a Flat, Flexible School Shoe in Adolescents with Patellofemoral Pain.** *Medicine and Science in Sports and Exercise* 2024, **56:**745-752.

69. Medina-Alcantara M, Morales-Asencio JM, Jiménez-Cebrián AM, Paez-Moguer J, Cervera-Marin JA, Gijon-Nogueron G, Ortega-Avila AB: **Influence of Shoe Characteristics on the Development of Valgus Foot in Children.** *Journal of clinical medicine* 2019, **8:**85-NA.

70. Mullen S, Toby EB: **Adolescent runners: the effect of training shoes on running kinematics.** *Journal of Pediatric Orthopedics* 2013, **33:**453-457.

71. Munuera PV, Castillo JM, Dominguez G, Lafuente G: **Orthotic devices with out-toeing wedge as treatment for in-toed gait in children.** *Journal of the American Podiatric Medical Association* 2010, **100:**472-478.

72. Mwai J, Nyole D, Abdi MH, Omogi J: **Factors associated with tungiasis among school-age children in Kwale County, rural Kenya.** *International health* 2023, **15:**85-92.

73. Mwangi JN, Ozwara H, Gicheru MM: **Epidemiology of tunga penetrans infestation in selected areas in Kiharu constituency, Murang’a County, Kenya.** *Tropical diseases, travel medicine and vaccines* 2015, **1:**13-13.

74. Oeffinger D, Brauch B, Cranfill S, Hisle C, Wynn C, Hicks R, Augsburger S: **Comparison of gait with and without shoes in children.** *Gait & Posture* 1999, **9:**95-100.

75. Okai-Nobrega LA, Santos TRT, Lage AP, Araujo PAd, Souza TRd, Fonseca ST: **The Influence of the Shoe over the Medial Foot Arch and the Lower Limbs Kinematics in Toddlers.** *Revista Brasileira de Ortopedia* 2022, **57:**167-174.

76. Okai-Nobrega LA, Santos TRT, Lage AP, Araujo PAD, Souza TR, Fonseca ST: **Effects of Biomimetic Shoes on Healthy Young Children's Gait.** *Revista Brasileira de Ortopedia* 2022.

77. O'Kane JW, Gray KE, Levy MR, Neradilek M, Tencer AF, Polissar NL, Schiff MA: **Shoe and Field Surface Risk Factors for Acute Lower Extremity Injuries Among Female Youth Soccer Players.** *Clinical Journal of Sport Medicine* 2016, **26:**245-250.

78. Onder M, Atahan AC, Bassoy B: **Foot dermatitis from the shoes.** *International Journal of Dermatology* 2004, **43:**565-567.

79. Pizac DA, Swanik CB, Glutting JJ, Kaminski TW: **Evaluating Postural Control and Ankle Laxity Between Taping and High-Top Cleats in High School Football Players.** *Journal of Sport Rehabilitation* 2018, **27:**111-117.

80. Plesek J, Silvernail JF, Hamill J, Jandacka D: **FOOTFALL PATTERN DURING RUNNING IN PRESCHOOL CHILDREN ACCORDING TO AGE AND FOOTWEAR.** *NA* 2020, **38:**400-NA.

81. Plesek J, Freedman Silvernail J, Hamill J, Jandacka D: **Running Footstrike Patterns and Footwear in Habitually Shod Preschool Children.** *Medicine & Science in Sports & Exercise* 2021, **53:**1630-1637.

82. Plesek J, Hamill J, Blaschova D, Freedman-Silvernail J, Jandacka D: **Acute effects of footwear on running impact loading in the preschool years.** *Sports Biomechanics* 2023, **22:**442-458.

83. Puszczałowska-Lizis E, Lukasiewicz A, Lizis S, Omorczyk J: **The impact of functional excess of footwear on the foot shape of 7-year-old girls and boys.** *PeerJ* 2021, **9:**e11277-NA.

84. Puszczalowska-Lizis E, Zarzyczna P, Mikulakova W, Migala M, Jandzis S: **Influence of footwear fitting on feet morphology in 9 year old girls.** *BMC Pediatrics* 2020, **20:**349.

85. Puszczalowska-Lizis E, Zarzyczna P, Mikulakova W: **Impact of footwear fitting on foot shape in primary schoolgirls.** *Acta of Bioengineering & Biomechanics* 2020, **22:**119-126.

86. Puszczalowska-Lizis E, Lizis S, Prusak M, Omorczyk J: **Impact of length and width of footwear on foot structure of preschool-aged children.** *PeerJ* 2022, **10:**e13403.

87. Quinlan S, Sinclair P, Hunt A, Yan AF: **The long-term effects of wearing moderate minimalist shoes on a child's foot strength, muscle structure and balance: A randomised controlled trial.** *Gait & Posture* 2022, **92:**371-377.

88. Rao UB, Joseph B: **The influence of footwear on the prevalence of flat foot. A survey of 2300 children.** *Journal of Bone & Joint Surgery - British Volume* 1992, **74:**525-527.

89. Robinson LE, Rudisill ME, Weimar WH, Breslin CM, Shroyer JF, Morera M: **Footwear and locomotor skill performance in preschoolers.** *Perceptual & Motor Skills* 2011, **113:**534-538.

90. Schuttelaar ML, Meijer JM, Engfeldt M, Lapeere H, Goossens A, Bruze M, Persson C, Bergendorff O: **Allergic contact dermatitis caused by dimethylthiocarbamylbenzothiazole sulfide (DMTBS) in canvas shoes: in search of the culprit allergen.** *Contact Dermatitis* 2018, **78:**7-11.

91. Senden R, Marcellis R, Meijer K, Willems P, Lenssen T, Staal H, Janssen Y, Groen V, Vermeulen RJ, Witlox M: **Dataset of 3D gait analysis in typically developing children walking at three different speeds on an instrumented treadmill in virtual reality.** *Data in brief* 2023, **48:**109142.

92. Shields N, Lim P, Wollersheim D, Nikolopoulos N, Barrett JT, Evans A, Taylor NF, Munteanu SE: **Do foot posture, deformity, and footwear fit influence physical activity levels in children with Down syndrome? A prospective cohort study.** *Journal of Intellectual & Developmental Disability* 2016, **42:**332-338.

93. Shields N, Lim P, Wollersheim D, Nikolopoulos N, Barrett J, Evans A, Taylor NF, Munteanu S: **Do foot posture, deformity, and footwear fit influence physical activity levels in children with Down syndrome? A prospective cohort study.** *Journal of Intellectual & Developmental Disability* 2017, **42:**332-338.

94. Shultz SP, Houltham SD, Kung SM, Hume P, Fink PW: **Metabolic Differences Between Shod and Barefoot Walking in Children.** *International Journal of Sports Medicine* 2016, **37:**401-404.

95. Smiley SJ, Jacobsen FS, Mielke C, Johnston R, Park C, Ovaska GJ: **A comparison of the effects of solid, articulated, and posterior leaf-spring ankle-foot orthoses and shoes alone on gait and energy expenditure in children with spastic diplegic cerebral palsy.** *Orthopedics* 2002, **25:**411-415.

96. Staheli LT, Giffin L: **Corrective shoes for children: a survey of current practice.** *Pediatrics* 1980, **65:**13-17.

97. Tan JSY: **The balance control of young children under different shod conditions in a naturalistic environment.** *Gait & Posture* 2019, **68:**68-71.

98. Taylor JB, Nguyen AD, Griffin JR, Ford KR: **Effects of turf and cleat footwear on plantar load distributions in adolescent American football players during resisted pushing.** *Sports Biomechanics* 2018, **17:**227-237.

99. Teixeira M, Machado S, Teixeira A, Silva E: **Severe contact allergy to footwear in a young child.** *Contact Dermatitis* 2005, **52:**159-160.

100. Tinker M, Betten A, Morris S, Gibson N, Allison G, Ng L, Williams G, Chappell A: **A comparison of the kinematics and kinetics of barefoot and shod running in children with cerebral palsy.** *Gait and Posture* 2022, **98:**271-278.

101. Tong JW, Kong PW: **Medial Longitudinal Arch Development of Children Aged 7 to 9 Years: Longitudinal Investigation.** *Physical Therapy* 2016, **96:**1216-1224.

102. Tora A, Tadele G, Davey G, McBride CM: **The extent of protective footwear use among school-age rural children at high risk for podoconiosis and socio-economic correlates: A household cross-sectional survey in Southern Ethiopia.** *PLoS Neglected Tropical Diseases [electronic resource]* 2021, **15:**e0009791.

103. Traut AG, Hannigan JJ, Ter Har JA, Pollard CD: **Influence of Footwear Selection on Youth Running Biomechanics: A Pilot Study.** *Sports Health* 2023.

104. Trevisan G, Kokelj F: **Allergic contact dermatitis due to shoes in children: a 5-year follow-up.** *Contact Dermatitis* 1992, **26:**45.

105. Walter JH, Ng GK: **The evaluation of cleated shoes with the adolescent athlete in soccer.** *Foot* 2002, **12:**158-165.

106. Watanabe E, McBride CM, Tora A, Ayode DA, Farrell D, Davey G: **Use of footwear and foot condition among rural Ethiopian school children.** *Journal of Epidemiology and Global Health* 2014, **4:**323-325.

107. Wegener C, Greene A, Burns J, Hunt AE, Vanwanseele B, Smith RM: **In-shoe multi-segment foot kinematics of children during the propulsive phase of walking and running.** *Human Movement Science* 2015, **39:**200-211.

108. Williams C, Kolic J, Wu W, Paterson K: **Soft soled footwear has limited impact on toddler gait.** *PLoS ONE [Electronic Resource]* 2021, **16:**e0251175.

109. Wolf S, Simon J, Patikas D, Schuster W, Armbrust P, Doderlein L: **Foot motion in children shoes: a comparison of barefoot walking with shod walking in conventional and flexible shoes.** *Gait & Posture* 2008, **27:**51-59.

110. Yurt Y, Sener G, Yakut Y: **Footwear suitability in Turkish preschool-aged children.** *Prosthetics & Orthotics International* 2014, **38:**224-231.
