# Supplementary material for: Development of the CHILD‐SHOE Reporting Checklist: A Scoping Review and Modified Delphi Study to Support Reporting in Children's Footwear Research
Source: J Foot Ankle Res. 2025 Jul 9;18(3):e70065. doi: 10.1002/jfa2.70065 (PMC12241440; doi:10.1002/jfa2.70065)
Supplement: Supplementary file 3 — Supporting Information S3 [file JFA2-18-e70065-s004.pdf]

MONASH University
Medicine, Nursing and Health Sciences

## INTRO/CONSENT

Developing the CHILD's SHOE REPORT: A checklist of Information on children's footwear research reporting

Project ID: 43612

Research team:
Prof Cylie Williams
Jessica Kolic
A/Prof Kade Paterson
Dr Stewart Morrison
Dr Matthew Hill
Dr Melanie Farlie
Dr Helen Banwell

---

You have been provided the Explanatory Statement by

email.

**Please provide the preferred email you regularly access. This will be the email we use to link your data in each round:**

**Please repeat this email to confirm it is correct.**

**Please check ALL of the following boxes to take part in this research. If you don't wish to agree to ALL of these statements, you can close the browser now to exit the survey.**

- ☐ I have read the explanatory statement and had the opportunity to ask questions about this research
- ☐ I consent to take part in this research and will participate in the online Delphi panels to the best of my ability
- ☐ I will keep my responses confidential

## ABOUT YOU

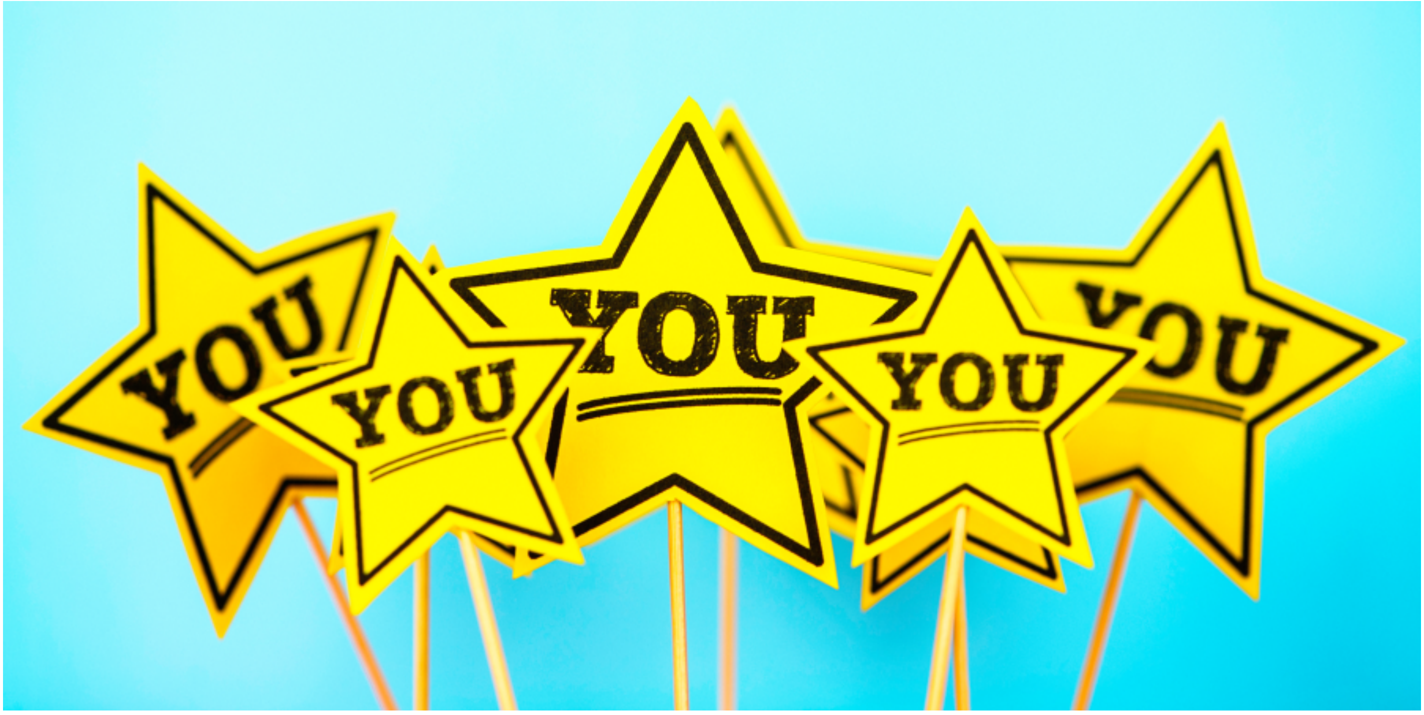


Provide your first and last name:

Please provide your gender:

Please select the country you reside in:

**Provide the number of peer-reviewed articles you have published (number only):**

**Please provide (number only) how many of your peer-reviewed publications are specific to children's footwear:**

**Select your highest attained qualification:**

- ○ Bachelor
- ○ Masters
- ○ PhD
- ○ Other (please detail)

**Please indicate your <u>preferred level of involvement</u> in this study. If this should change, we will contact you via the email you have provided.**

**<u>Please note</u> to qualify for authorship you will need to adhere to the ICMJE criteria, and:**

**(i) contribute to all three rounds of this Delphi**

**(ii) review the draft manuscript**

**(iii) approve the final manuscript for publication**

**(iv) agree to be responsible for the publication and adhere to the timelines set by the investigator team where applicable**

- ◯ Named author - agree to contribute to all rounds, review the manuscript, agree to final draft and agree to be responsible for final paper.
- ◯ Name acknowledgement in final paper
- ◯ No acknowledgement

## FOOTWEAR

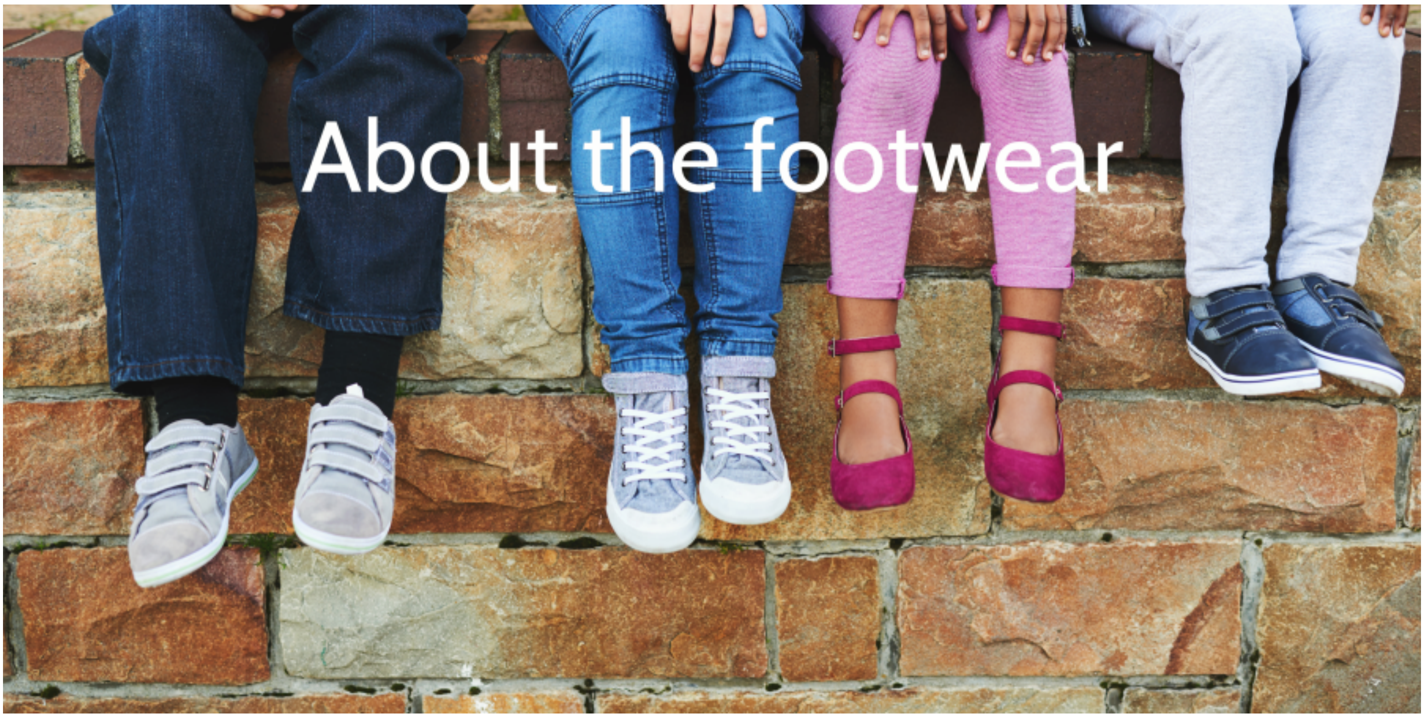


## Aim 1: Minimum footwear descriptions and features that should be reported in research.

This section includes themes related to footwear descriptors and features that have been organised into domains where possible. Individual items have been identified where applicable within each domain.

All domains and corresponding items listed here for consideration were extracted from a systematic scoping review of 115 publications conducted by members of the research team.

**Step 1:**
Please consider each domain and identify if each item should be reported in research relating to children's footwear on the provided scale of agreement (e.g. from NEVER, SOMETIMES or ALWAYS).

**Step 2:**
If you indicate the domain should be reported, please consider the individual listed items within that domain where available and identify your level of agreement.

Please consider each very carefully as the overall aim is to provide a consensus statement for reporting standards in children's footwear research.

You will have the opportunity to provide comments at the end of each section.

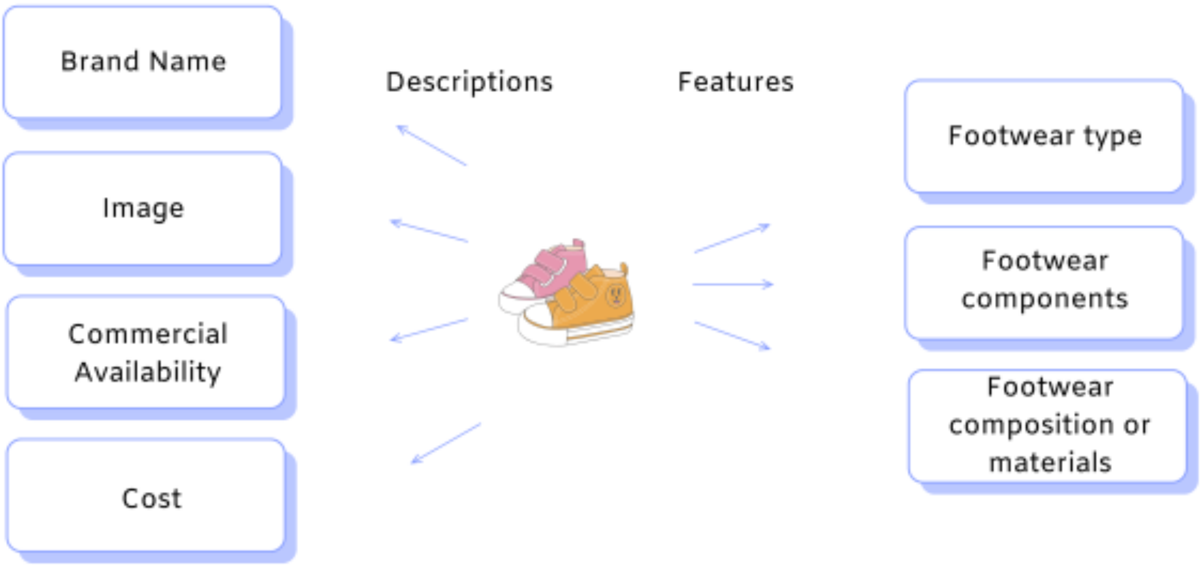


Studies reported four **footwear descriptions** elements and three **footwear feature** elements. Rate your agreement on each as an element of interest in relation to how important it is to you that the description/feature is included when reporting research related to chdilren's footwear.

*The 'n' indicates how often this was reported in the included studies.*

| | This should NEVER be reported | This should SOMETIMES be reported | This should ALWAYS be reported |
|---|---|---|---|
| Brand name (n=26) | ○ | ○ | ○ |
| Includes an image of the footwear (n=49) | ○ | ○ | ○ |
| Commercial availability (n=2) | ○ | ○ | ○ |
| Footwear retail cost (n=1) | ○ | ○ | ○ |
| Footwear type (n=80) | ○ | ○ | ○ |
| Components of the footwear (n=66) | ○ | ○ | ○ |
| Composition or materials of the footwear (n=46) | ○ | ○ | ○ |

You have indicated that **footwear types** should sometimes or always be described as a footwear **feature** in children's footwear research.

Please consider the following individual items or terms we have extracted from the literature and we aligned against **footwear types.**

Rate the suitability of each item or term in relation to how important it is to you that they are included within the footwear **features** domain when reporting **footwear types** within research related to children's footwear.

This will form a list of **footwear types** that a researcher should pick from.

| | Disagree this item should be reported | Neutral this item should reported | Agree this item should be reported |
|---|---|---|---|
| Sandal, flip flops, slides (n=17)Fe | ○ | ○ | ○ |
| Slipper/indoor shoe (n=10) | ○ | ○ | ○ |
| Biomemetic/function/minimalist footwear (n=13) | ○ | ○ | ○ |
| General/casual shoe (n=5) | ○ | ○ | ○ |
| Mary Jane/Ballet flat/t-bar shoe(n=2) | ○ | ○ | ○ |
| Boot (n=2) | ○ | ○ | ○ |
| Pre-walker or soft soled shoe (n=2) | ○ | ○ | ○ |
| School shoe (oxford style) (n=13) | ○ | ○ | ○ |
| Medical/orthopaedic shoe (n=3) | ○ | ○ | ○ |
| Sport specific (sport listed) shoe (n=10) | ○ | ○ | ○ |

| | | | |
|---|---|---|---|
| Sneakers, Runners, Trainers or Sport/Athletic shoes (n=37) | ○ | ○ | ○ |
| Outdoor shoe (n=1) | ○ | ○ | ○ |

You have indicated that **footwear components** should sometimes or always be described as a footwear feature in children's footwear research.

Please consider the following individual items or terms we have extracted from the literature and we aligned against **footwear components.**

Rate the suitability of each item or term in relation of how important it is to you that they are included within the **footwear features domain** when reporting **footwear components** within research related to children's footwear.

This will form a list of **footwear components** that a researcher should pick from.

| | Disagree this item should be reported | Neutral this item should reported | Agree this item should be reported |
|---|---|---|---|

| | | | |
|---|---|---|---|
| Heel counter presence and/or it's stiffness (n=191) | ○ | ○ | ○ |
| Upper of shoe covers full or part of foot (n=26) | ○ | ○ | ○ |
| Outsole with/without separate heel (n=8) | ○ | ○ | ○ |
| Sole flexibility (n=21) | ○ | ○ | ○ |
| Insole materials in shoe (n=14) | ○ | ○ | ○ |
| Topline of shoe in relation to the ankle (n=16) | ○ | ○ | ○ |
| Mass (e.g. grams) of shoe (n=18) | ○ | ○ | ○ |
| Sole shape (including last) of shoe (n=11) | ○ | ○ | ○ |
| Fixtures (e.g velcro, laces etc) of shoe (n=35) | ○ | ○ | ○ |
| Toe box (shape and/or height) of upper (n=9) | ○ | ○ | ○ |
| Pitch, drop and/or stack of outsole (n=32) | ○ | ○ | ○ |
| Slip resistance of outsole (n=3) | | | |

| | | | |
|---|---|---|---|
| | ○ | ○ | ○ |
| Minimalist index of footwear (n=3) | ○ | ○ | ○ |
| Presence, amount and location of sole rocker (n=1) | ○ | ○ | ○ |
| Footwear colour (n=1) | ○ | ○ | ○ |

You have indicated that **footwear composition** should sometimes or always be described as a footwear feature in children's footwear research.

Please consider the following individual items or terms we have extracted from the literature and we aligned against **footwear composition.**

Rate the auitability of each item or term in relation of how important it is to you that they are included within the **footwear features domain** when reporting **footwear composition** within research related to children's footwear.

This will form a list of **footwear composition** that a researcher should pick from.

| | Disagree this item should be reported | Neutral this item should reported | Agree this item should be reported |
|---|---|---|---|
| Glue and or adhesives used (n=2) | ○ | ○ | ○ |
| Upper material (n=38) | ○ | ○ | ○ |
| Sole material and density (n=25) | ○ | ○ | ○ |

If you have disagreed with any of the items, how they have been aligned or believe some are missing please comment below.

## OUTCOMES

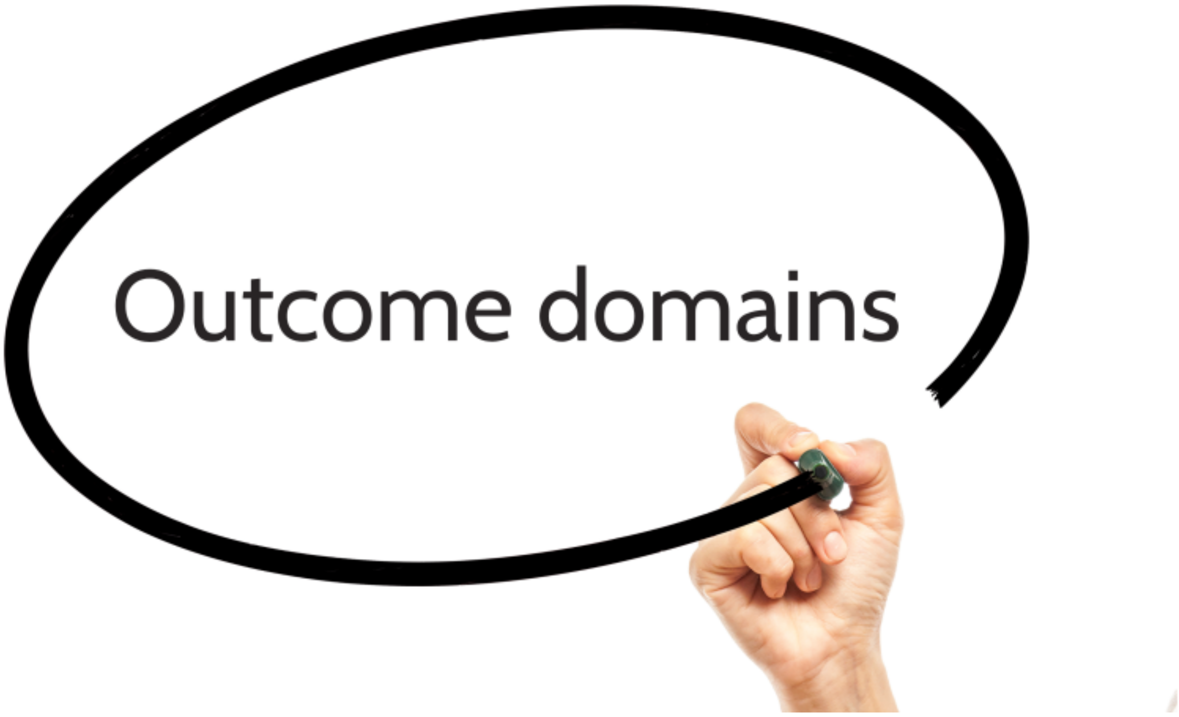


**Aim 2: Minimum outcomes relating to the impact of children's footwear that should be reported in research.**

This section includes all extracted data related to outcome measures as reported in children's shoe research from our scoping review. We have organised these outcomes against the "The 'F-words' in Childhood Disability" (Ref: Rosenbaum P, Child: care, health and development. 2012 Jul;38(4):457-63.)

This paper describes six F-words that should be of focus in childhood research and relate to the International Classification of Functioning, Disability and Health (ICF).

We have made a graphic to orient you to this framework. We will use this framework as we take you through the outcome measures we have extracted from the literature.

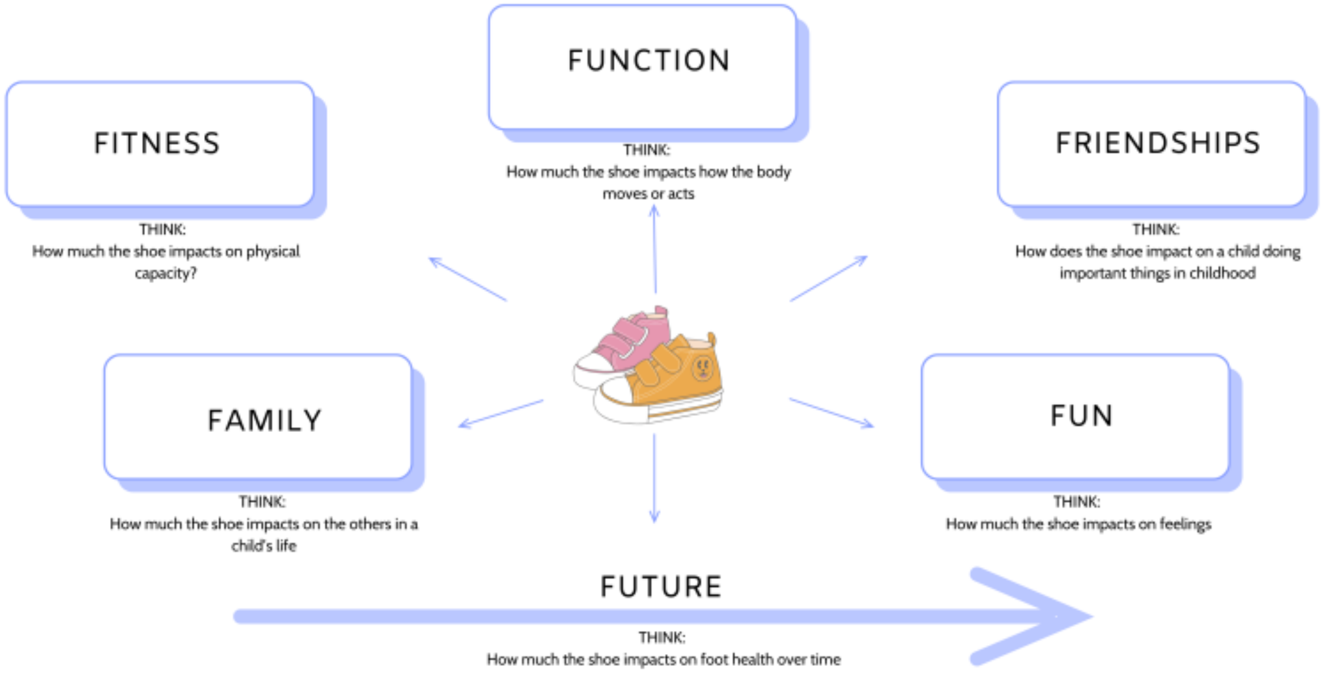


Similar to the information you have already seen about footwear, you will be presented with information relating to each of the above domains.

**Step 1:**
Please consider each domain and identify if they should ALWAYS, SOMETIMES or NEVER be reported in research relating to children's footwear.
**Step 2:**

If you indicate a domain should be reported, please consider the individual listed items where available and identify your level of agreement using the provided scale.

Please consider each very carefully as the overall aim is to provide a consensus statement for reporting standards in children's footwear research.

You will have the opportunity to also provide comments.

There were six themes identified as aligning to the **FUNCTION** outcome domain. Please rate your agreement with each of the outcome measure groups in relation to them fitting within this domain.

*The 'n' indicates how often this was reported in the included studies.*

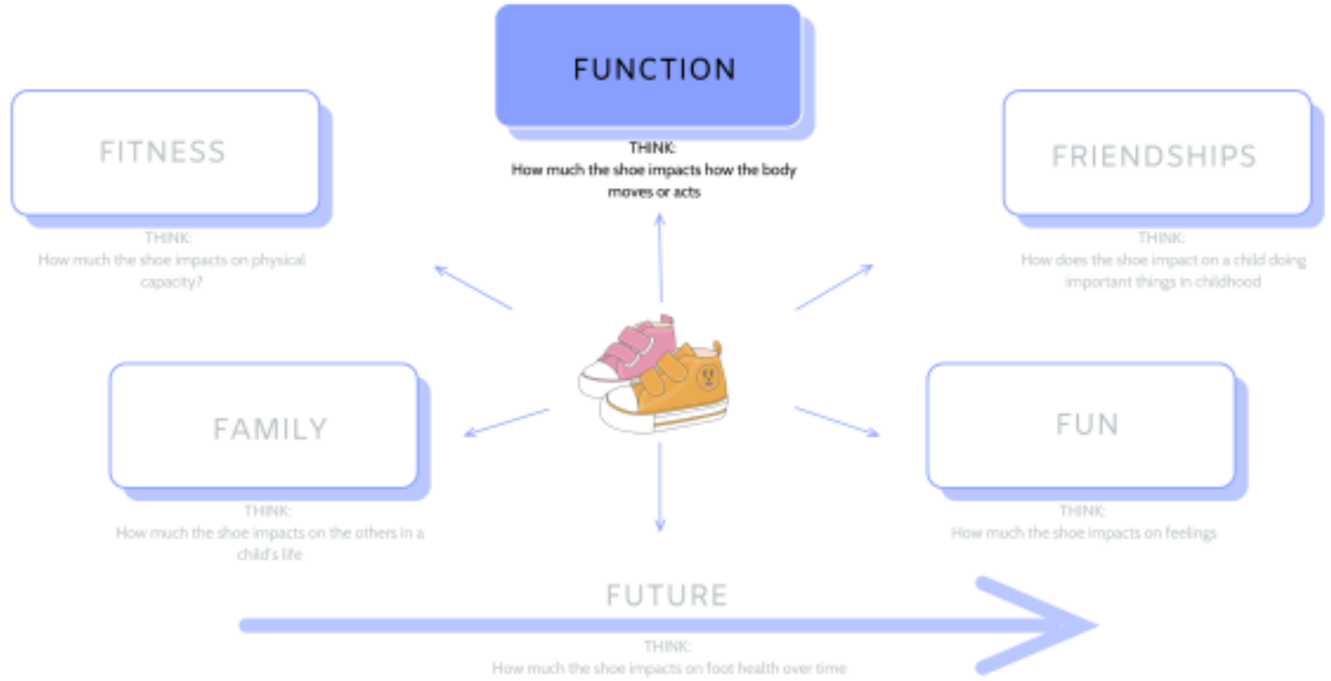


| | This should NEVER be reported | This should SOMETIMES be reported | This should ALWAYS be reported |
|---|---|---|---|
| Presence of infection (n=3) | ○ | ○ | ○ |
| Spatiotemporal measures (n=41) | ○ | ○ | ○ |
| Plantar pressure (n=10) | ○ | ○ | ○ |
| Kinematics and Kinetics (n=38) | ○ | ○ | ○ |
| Foot features (n=29) | ○ | ○ | ○ |
| Balance and gross motor function (n=10) | ○ | ○ | ○ |

You have indicated that **spatiotemporal** outcome measures should be aligned with the **FUNCTION** domain.

We extracted the following items in our scoping review and propose they align with spatiotemporal measures. Please rate your level of agreement with this alignment.

*The n indicates how often this was reported in the included studies.*

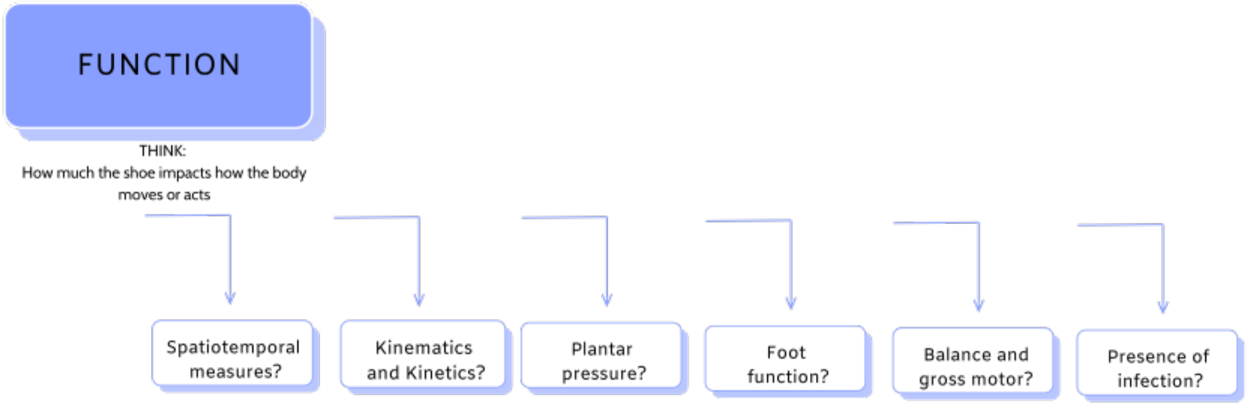


| | Disagree this item should be reported | Neutral this item should reported | Agree this item should be reported |
|---|---|---|---|
| Velocity (m/s) (n=30) | ○ | ○ | ○ |
| | ○ | ○ | ○ |

| | | | |
|---|---|---|---|
| Cadence (steps/sec) (n=15) | | | |
| Stride length (n=16) | ○ | ○ | ○ |
| Step length (n=12) | ○ | ○ | ○ |
| Base of support (n=2) | ○ | ○ | ○ |
| Stance time (n=4) | ○ | ○ | ○ |
| Step time (n=3) | ○ | ○ | ○ |
| Toe in/out angle (n=6) | ○ | ○ | ○ |
| Step width (n=6) | ○ | ○ | ○ |
| Double/single support time (n=3) | ○ | ○ | ○ |
| Contact time (n=2) | ○ | ○ | ○ |
| Stance phase % (n=7) | ○ | ○ | ○ |
| Swing phase % (n=5) | ○ | ○ | ○ |
| Stride time (n=2) | ○ | ○ | ○ |
| Number of steps (n=1) | ○ | ○ | ○ |

You have indicated that **kinematics and kinetic** outcome measures should be described against the **FUNCTION** domain.

We extracted the following items in our scoping review and propose they align with kinematic and kinetic measures. Please rate your level of agreement with this alignment.

*The n indicates how often this was reported in the included studies.*

In the interest of your time, we have grouped body regions.

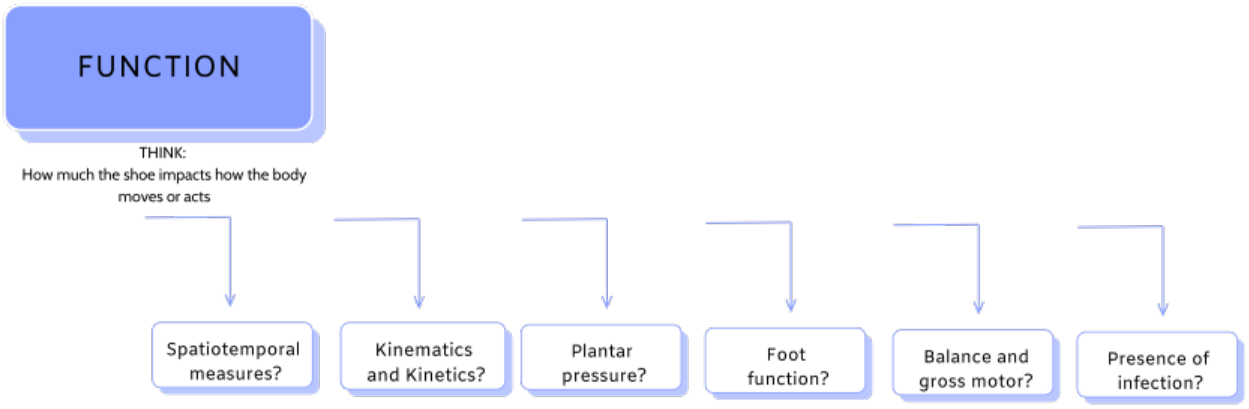


| | Disagree this item should be reported | Neutral this item should reported | Agree this item should be reported |
|---|---|---|---|
| Hip region angles (n=22) | ○ | ○ | ○ |

| | | | |
|---|---|---|---|
| Knee region angles (n=27) | ○ | ○ | ○ |
| Ankle region angles (n=33) | ○ | ○ | ○ |
| Foot region angles (n=29) | ○ | ○ | ○ |
| Ground reaction force (n=19) | ○ | ○ | ○ |
| Vertical loading (n=2) | ○ | ○ | ○ |
| Joint power (n=3) | ○ | ○ | ○ |
| Impulse (n=1) | ○ | ○ | ○ |
| Peak pressure (n=1) | ○ | ○ | ○ |
| Centre of mass displacement (n=2) | ○ | ○ | ○ |
| Ground reaction time (n=1) | ○ | ○ | ○ |

You have indicated that **plantar pressure** measures should be described against the **FUNCTION** domain.

We extracted the following items in our scoping review and propose they align plantar pressure measures. Please rate your level of agreement with this alignment.

*The n indicates how often this was reported in the*

*included studies.*

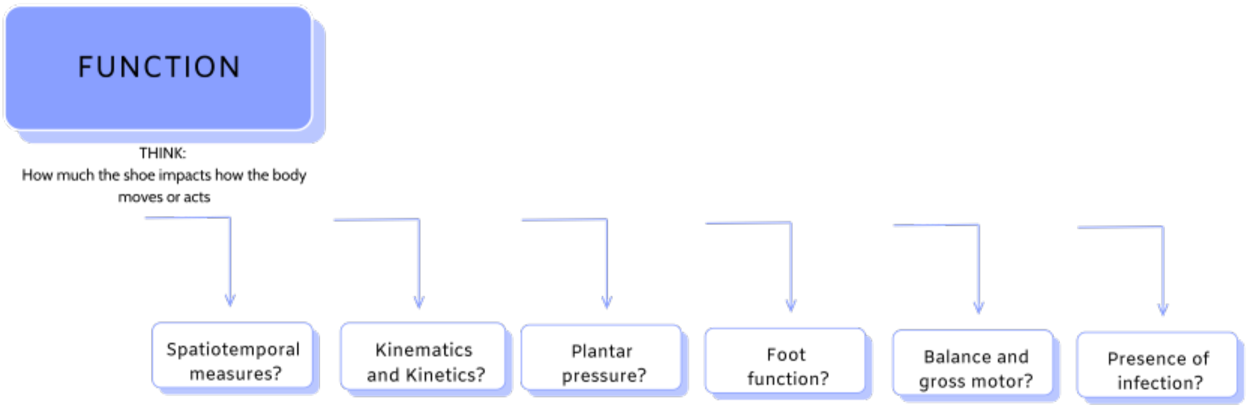


| | Disagree this item should be reported | Neutral this item should reported | Agree this item should be reported |
|---|---|---|---|
| Angle of gait (n=1) | ○ | ○ | ○ |
| % pressure fore and hind foot (n=5) | ○ | ○ | ○ |
| Mean pressure (n=2) | ○ | ○ | ○ |

You have indicated that measures of **foot features** should be described against the **FUNCTION** domain.

We extracted the following items in our scoping review and propose they align with measures of foot features.

Please rate your level of agreement with this alignment.

*The n indicates how often this was reported in the included studies.*

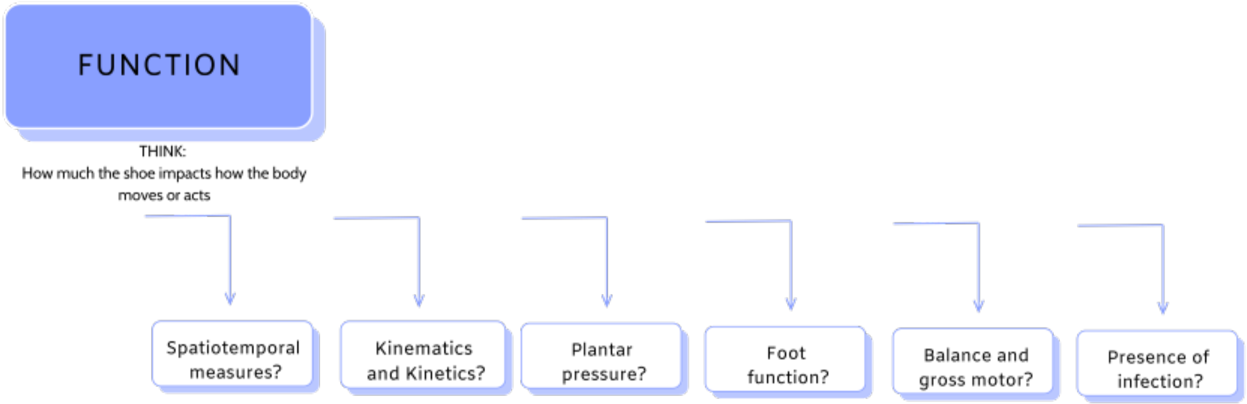


| | Disagree this item should be reported | Neutral this item should reported | Agree this item should be reported |
|---|---|---|---|
| Foot size (including width, length or volume) (n=7) | ○ | ○ | ○ |
| Skin/nail trauma (including allergy) (n=7) | ○ | ○ | ○ |
| Muscle size (n=1) | ○ | ○ | ○ |
| Wejsflogg Index (n=2) | ○ | ○ | ○ |
| Ankle range of movement (n=1) | ○ | ○ | ○ |

| | | | |
|---|---|---|---|
| Resting calcaneal stance position (n=3) | ○ | ○ | ○ |
| Fifth toe degree (n=1) | ○ | ○ | ○ |
| Instep height (n=1) | ○ | ○ | ○ |
| Arch index (n=4) | ○ | ○ | ○ |
| Foot posture Index (n=2) | ○ | ○ | ○ |
| Hallux valgus index (n=5) | ○ | ○ | ○ |
| Clarks angle (n=3) | ○ | ○ | ○ |

You have indicated that measures of **Balance and Gross Motor Function** should be described against the **FUNCTION** domain.

We extracted the following items in our scoping review and propose they align with measures of Gross Motor Function. Please rate your level of agreement with this alignment.

*The n indicates how often this was reported in the included studies.*

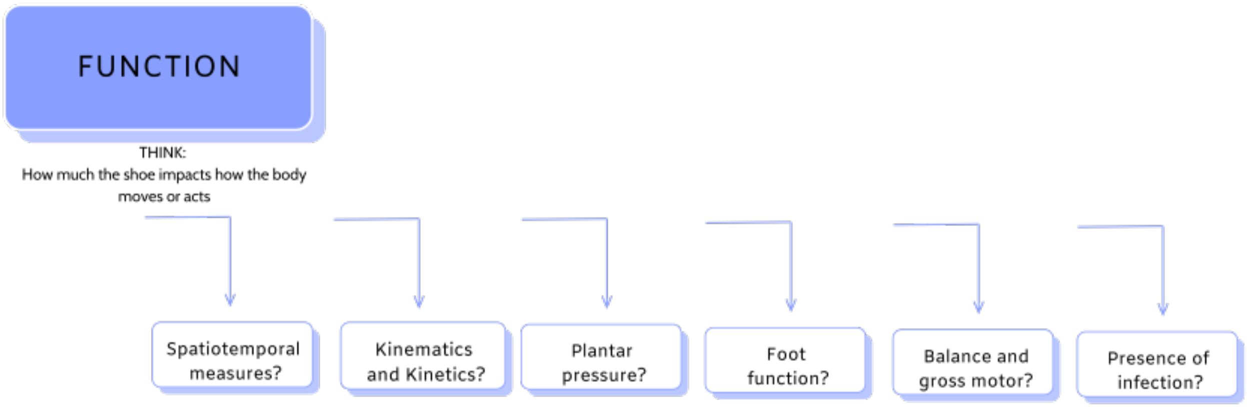


| | Disagree this item should be reported | Neutral this item should reported | Agree this item should be reported |
|---|---|---|---|
| Centre of pressure displacement (n=3) | ○ | ○ | ○ |
| Functional reach test (n=1) | ○ | ○ | ○ |
| Flamingo stance (n=1) | ○ | ○ | ○ |
| Number of falls (n=1) | ○ | ○ | ○ |
| Single leg stance (n=2) | ○ | ○ | ○ |
| Modified Balance Error Scoring System (M-BESS) (n=2) | ○ | ○ | ○ |

| | | | |
|---|---|---|---|
| Bruininks-Oseretsky Test of Motor Proficiency (BOT-2) (n=1) | ○ | ○ | ○ |
| Balance error score (n=1) | ○ | ○ | ○ |
| Four square step test (n=1) | ○ | ○ | ○ |
| Standing long jump (n=2) | ○ | ○ | ○ |
| Test of gross motor development (n=1) | ○ | ○ | ○ |
| Hoffer ambulation score (n=1) | ○ | ○ | ○ |
| Tip toe walking (n=1) | ○ | ○ | ○ |
| Dynamic hop (n=1) | ○ | ○ | ○ |
| Edinburgh Gait Scale (n=1) | ○ | ○ | ○ |
| Y test (n=1) | ○ | ○ | ○ |
| Timed up and go (n=1) | ○ | ○ | ○ |
| Lower limb muscle strength (with dynonometer) n=1) | ○ | ○ | ○ |

If you have disagreed with any of the FUNCTION outcome domains and responses, how they have been

aligned or believe some are missing please comment below.

There were three overarching elements we identified as aligning to the **FITNESS** outcome domain.

Please rate your agreement with each outcome measure grouping term sitting in this outcome domain.

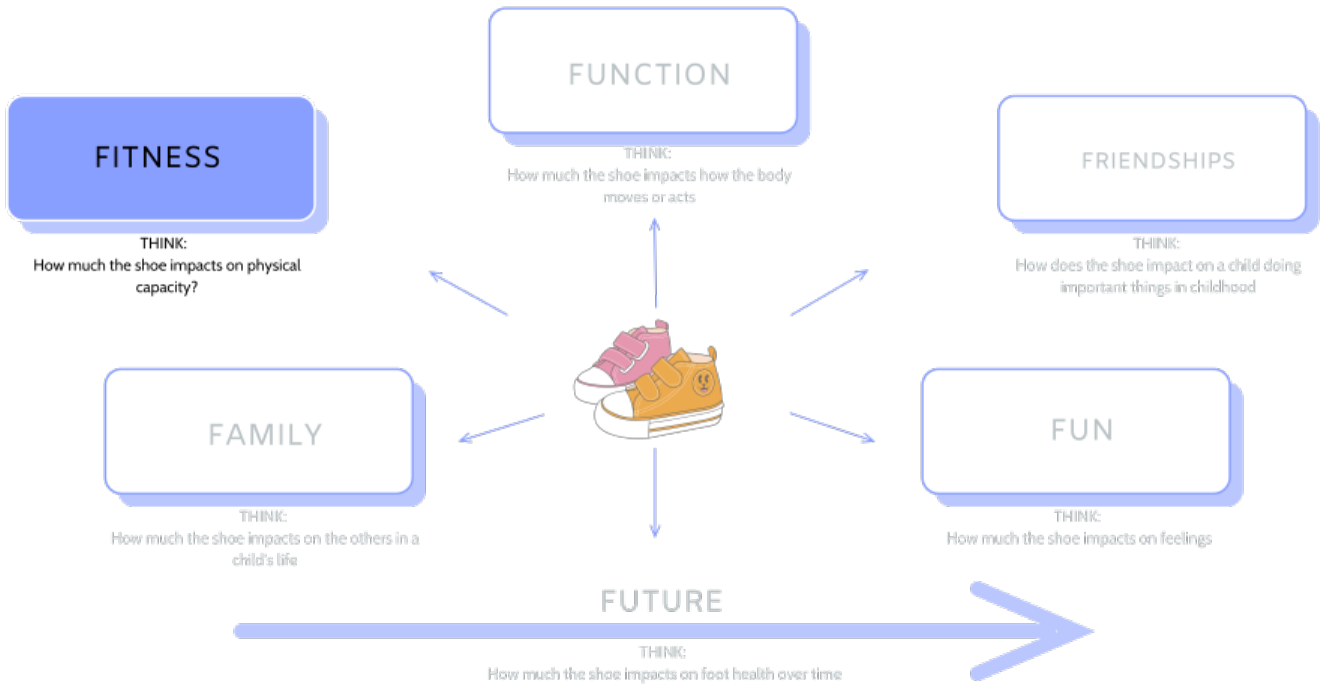

| | This should NEVER be reported | This should SOMETIMES be reported | This should ALWAYS be reported |
|---|---|---|---|
| Physical activity measures (n=8) | ○ | ○ | ○ |
| EMG (n=1) | ○ | ○ | ○ |
| Endurance measures (n=2) | ○ | ○ | ○ |

You have indicated that measures of **Physical Activity** should be described against the **FITNESS** domain.

We extracted the following items in our scoping review and propose they align with measures of Physical Activity. Please rate your level of agreement with this alignment.

*The n indicates how often this was reported in the included studies.*

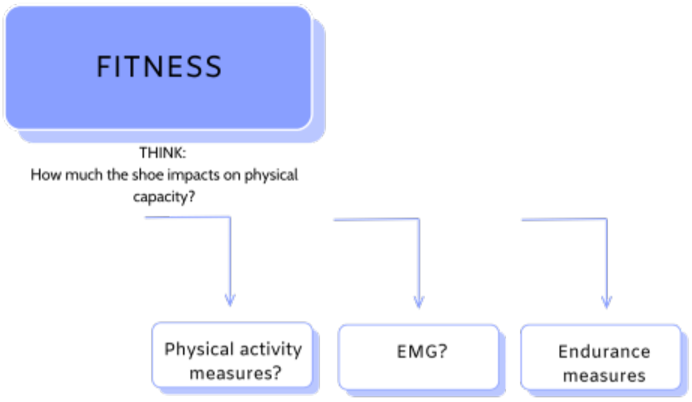


| | Disagree this item should be reported | Neutral this item should reported | Agree this item should be reported |
|---|---|---|---|
| Steps per day (n=2) | ○ | ○ | ○ |
| Physical activity diary (n=2) | ○ | ○ | ○ |
| Functional Mobility Scale (n=1) | ○ | ○ | ○ |
| Ball contact (n=1) | ○ | ○ | ○ |
| Moderate to vigorous physical activity (MVPA) (n=1) | ○ | ○ | ○ |
| VO-2 (n=1) | ○ | ○ | ○ |
| Energy Expenditure (n=2) | ○ | ○ | ○ |

| | | | |
|---|---|---|---|
| Borg Scale of Perceived Exertion (n=2) | ○ | ○ | ○ |

You have indicated that measures of **Endurance** should be described against the **FITNESS** domain.

We extracted the following items in our scoping review and propose they align with measures of Endurance. Please rate your level of agreement with this alignment.

*The n indicates how often this was reported in the included studies.*

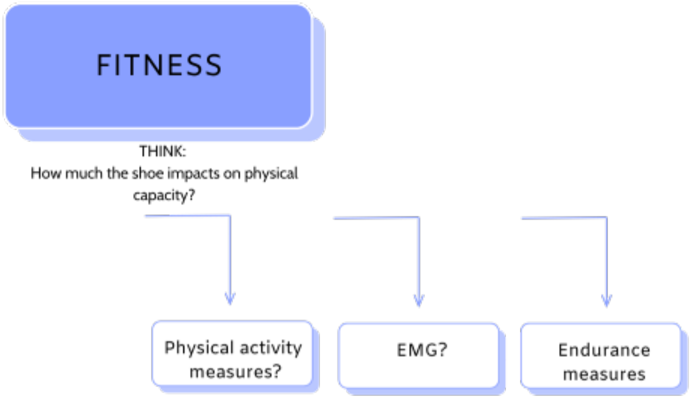


| | Disagree this item should be reported | Neutral this item should reported | Agree this item should be reported |
|---|---|---|---|

| | | | |
|---|---|---|---|
| 6 minute walk test (n=1) | ○ | ○ | ○ |
| 10 minute walk test (n=1) | ○ | ○ | ○ |

If you have disagreed with any of the FITNESS outcome domains and responses, how they have been aligned or believe some are missing please comment. below.

There was one overarching element we identified as aligning to the **FRIENDSHIP** outcome domain.

Please rate your agreement with this outcome measure grouping term sitting in this outcome domain.

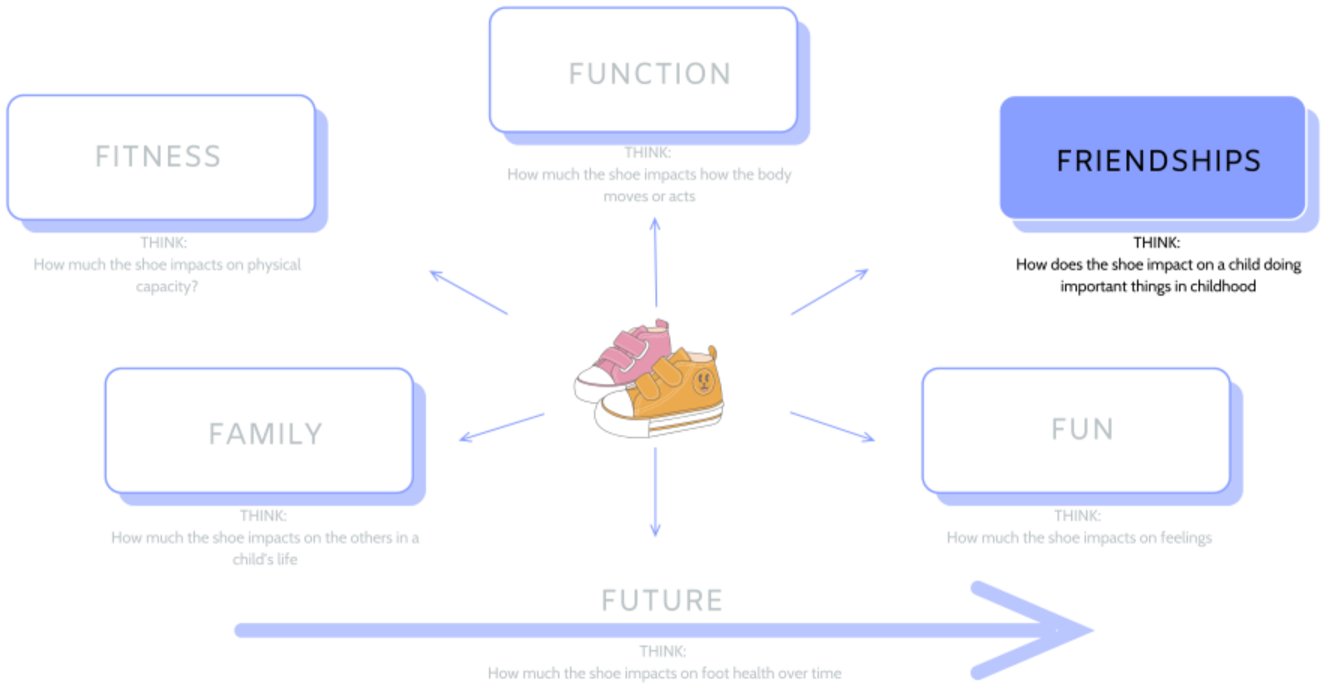


| | This should NEVER be reported | This should SOMETIMES be reported | This should ALWAYS be reported |
|---|---|---|---|
| Quality of life (n=4) | ○ | ○ | ○ |

You have indicated that measures of **Quality of Life** should be described against the **FRIENDSHIP** domain.

We extracted the following items in our scoping review and propose they align with Quality of Life measures. Please rate your level of agreement with this alignment.

*The n indicates how often this was reported in the included studies.*

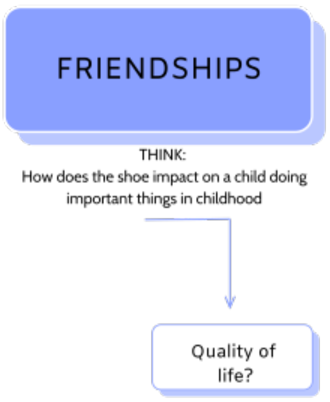


| | Disagree this item should be reported | Neutral this item should reported | Agree this item should be reported |
|---|---|---|---|
| Oxford Ankle Foot Questionnaire - Child (n=2) | | | |
| Knee Injury and Osteoarthritis Outcome Score - Child (n=1) | | | |
| Anterior Knee Pain Scale (n=1) | | | |
| Youth Quality of Life (n=1) | | | |
| Peds-QL (n=1) | | | |

If you have disagreed with any of the FRIENDSHIP outcome domains and responses, how they have been

aligned or believe some are missing please comment below.

There was two overarching elements we identified as aligning to the **FUN** outcome domain.

Please rate your agreement with these outcome measure grouping term sitting in this outcome domain.

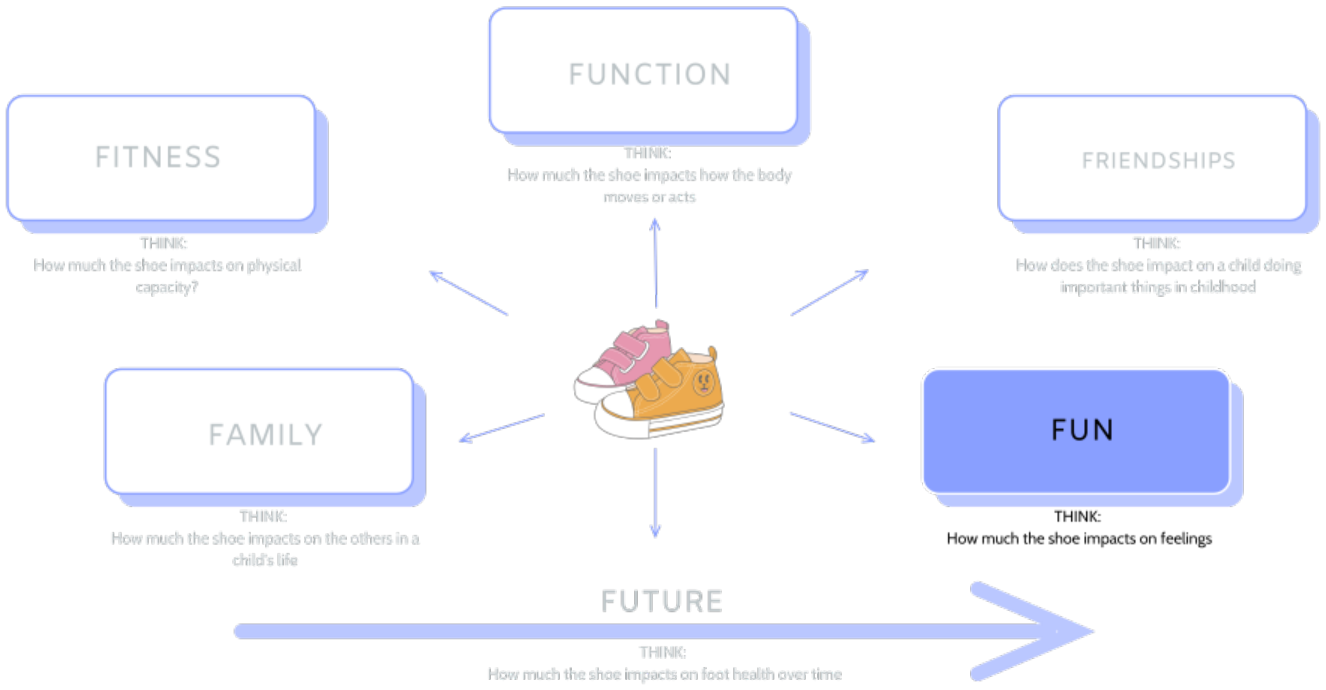


This should

| | This should NEVER be reported | SOMETIMES be reported | This should ALWAYS be reported |
|---|---|---|---|
| Comfort (n=20) | ○ | ○ | ○ |
| Body image perception relating to feet look (n=1) | ○ | ○ | ○ |

You have indicated that measures of **Comfort** should be described against the **FUN** domain.

We extracted the following items in our scoping review and propose they align with Comfort measures. Please rate your level of agreement with this alignment.

*The n indicates how often this was reported in the included studies.*

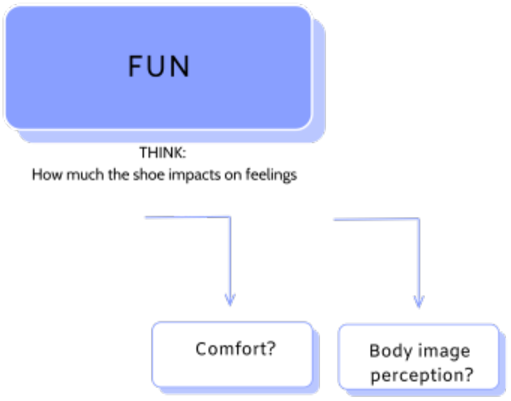


| | Disagree this item should be reported | Neutral this item should reported | Agree this item should be reported |
|---|---|---|---|
| Perception of fit (n=1) | ○ | ○ | ○ |
| Actual shoe fit to foot (e.g. measured with the cleverness device) (n=12) | ○ | ○ | ○ |
| Number of injuries (n=2) | ○ | ○ | ○ |
| Pain (n=2) | ○ | ○ | ○ |
| Foot assessment score (n=1/Jess) | ○ | ○ | ○ |
| Footwear comfort (Binary/VAS) (n=4) | ○ | ○ | ○ |
| Shoe wear distortion (n=1) | ○ | ○ | ○ |

If you have disagreed with any of the FUN outcome domains and responses, how they have been aligned or believe some are missing please comment below.

There was two overarching elements we identified as aligning to the **FAMILY** outcome domain.

Please rate your agreement with these outcome measure grouping term sitting in this outcome domain.

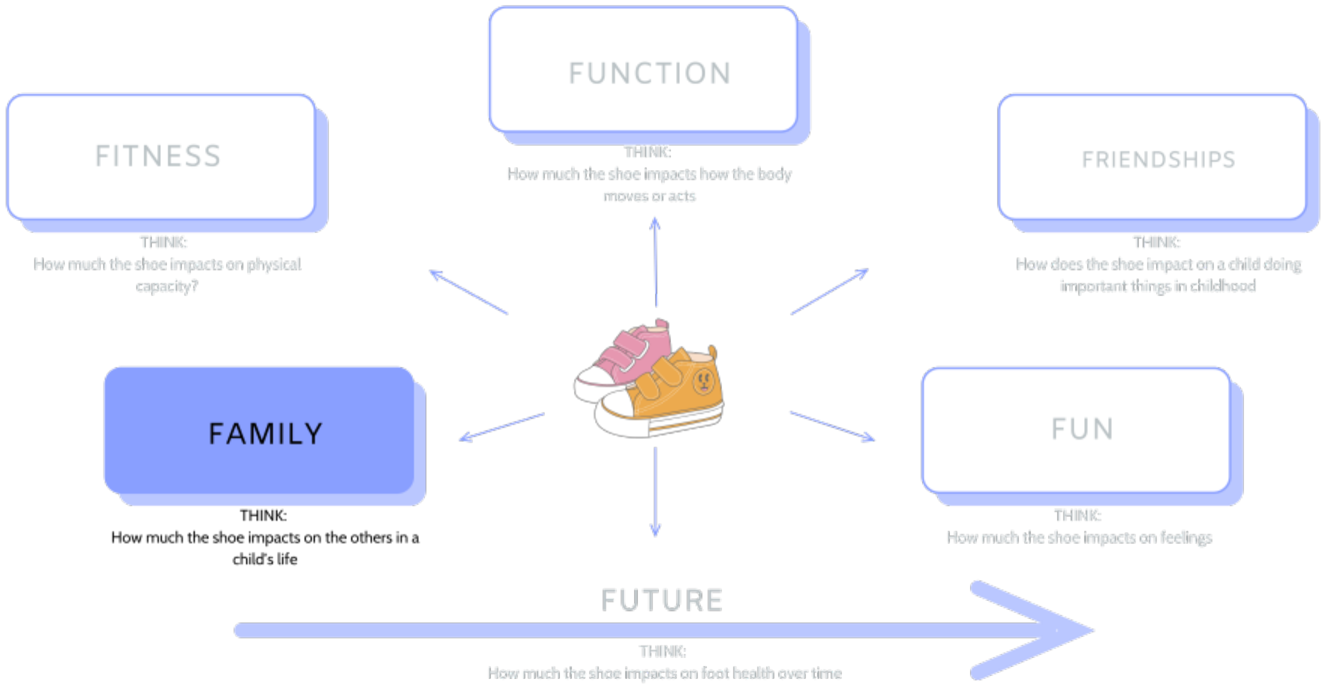

| | This should NEVER be reported | This should SOMETIMES be reported | This should ALWAYS be reported |
|---|---|---|---|
| Wear time (days and/or hours) (n=7) | ○ | ○ | ○ |
| Social factors (n=4) | ○ | ○ | ○ |

You have indicated that measures of **Social Factors** should be described against the **FAMILY** domain.

We extracted the following items in our scoping review and propose they align with Comfort measures. Please rate your level of agreement with this alignment.

*The n indicates how often this was reported in the included studies.*

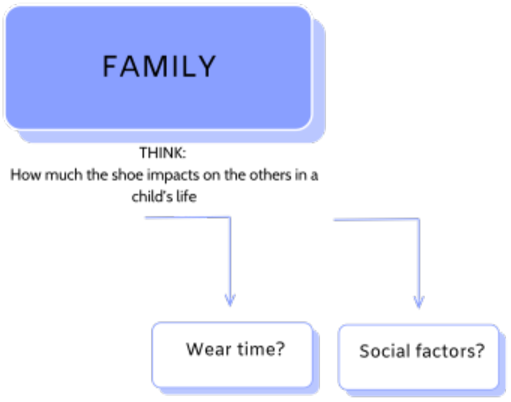


| | Disagree this item should be reported | Neutral this item should reported | Agree this item should be reported |
|---|---|---|---|
| Patterns of ownership (n=2) | ○ | ○ | ○ |
| Recommendations from health professionals (n=2) | ○ | ○ | ○ |

If you have disagreed with any of the FAMILY outcome domains and responses, how they have been aligned or believe some are missing please comment below.

There was two overarching elements we identified as aligning to the **FUTURE** outcome domain.

Please rate your agreement with these outcome measure grouping term sitting in this outcome domain.

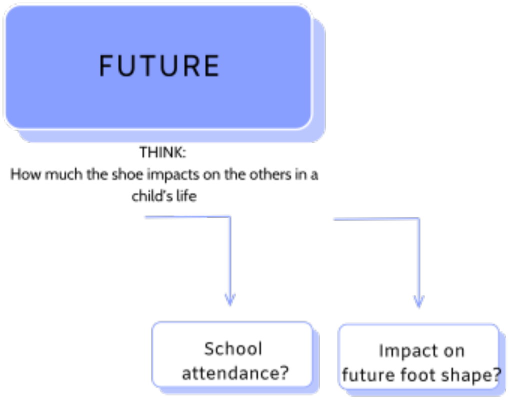


| | This should NEVER be reported | This should SOMETIMES be reported | This should ALWAYS be reported |
|---|---|---|---|
| School attendance (n=1) | ○ | ○ | ○ |
| Longitudinal impact of footwear on foot shape (n=7) | ○ | ○ | ○ |

If you have disagreed with any of the FUTURE outcome domains and responses, how they have been aligned or believe some are missing please comment below.

## End

Thank you for your views. We will contact you via email in 2 to 4 weeks from the close of this survey. The next survey will ask you to rate your agreement based on the responses received from this round.

The next survey is anticipated to be shorter

**It is important to meet the aims of this research that you keep taking part in each round, so we thank you for your continued involvement.**

Powered by Qualtrics
