# Supplementary material for: Development of the CHILD‐SHOE Reporting Checklist: A Scoping Review and Modified Delphi Study to Support Reporting in Children's Footwear Research
Source: J Foot Ankle Res. 2025 Jul 9;18(3):e70065. doi: 10.1002/jfa2.70065 (PMC12241440; doi:10.1002/jfa2.70065)
Supplement: Supplementary file 4 — Supporting Information S4 [file JFA2-18-e70065-s002.pdf]

INTRO/CONSENT

CHILD's SHOE REPORT: A checklist of Information on children's footwear research reporting- Round 2

What does my participation involve in this round?

We have collated the responses from the first round.

We had 32 people take part in helping to define the ways to describe footwear or the outcomes measuring the impact of the footwear in children.

Where greater than 70% of all people who took part agreed, a consensus was achieved and we included the domain and/or elements.

Where greater than 50%-69% of people agreed, we have included it as a statement for you to rate how much you agree at this stage. Where less than 50% of people responded the similarly, this has not been included in this round.

Where a new outcome element was proposed, we have also included it in this round for you to rate your agreement. Where you proposed a new element, and it was already in a different section, we have kept it in the original section as there was no consensus for moving elements to different domains.

Please remember, this is about setting the "minimum footwear descriptions

and outcomes" as a guide for researchers undertaking research about children's footwear research. During publication development, we will provide suggestions where studies may have specific questions or aims, and they would use this for consistency in reporting.

You can download a copy of the item progression and % of agreement at this link to guide your responses and see what did and didn't make this round: Round 2 child shoe

To meet the aim of this research, it is very important you complete this survey to give us your opinion of the statements. This is also essential for you to complete this if you have indicated you wish to contribute to authorship on a final version of a consensus statement.

It should take under 10 minutes.

If you would like a copy of your original responses in Round 1, please contact Jessica: Jessica.Kolic@monash.edu

Please provide your email below so we can track responses and link them between each round. Please use your same email for each round.

Footwear

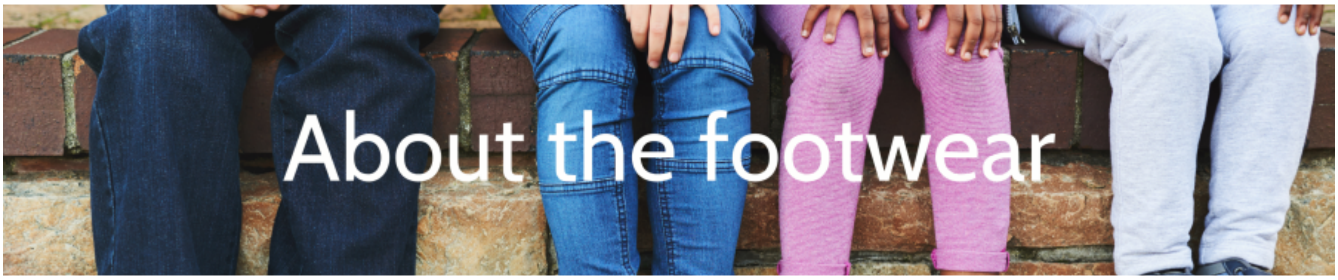

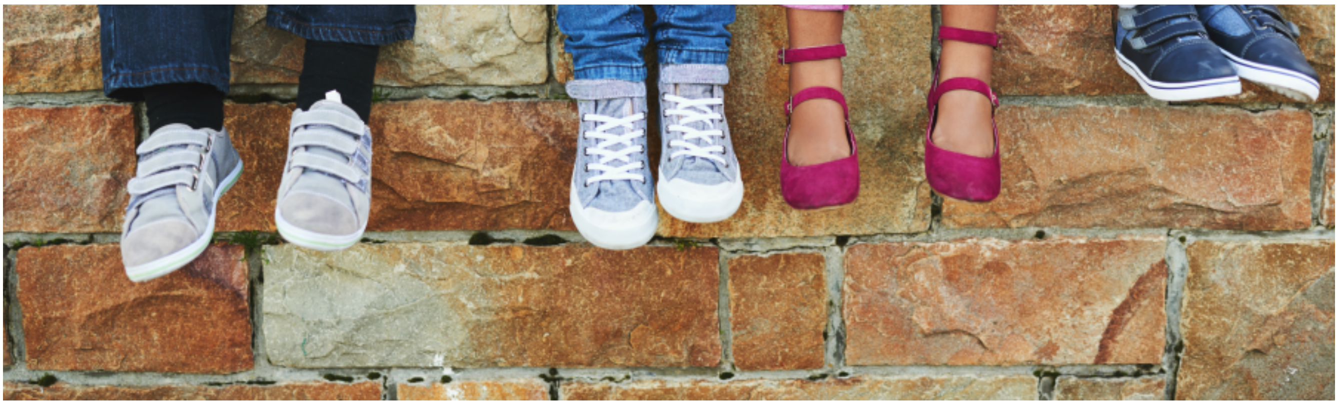

Aim 1: Minimum footwear descriptions and features that should be reported in research.

This section includes items related to footwear descriptors and features that have been organised into domains where possible.

Individual items have been identified where applicable within each domain.

All domains and corresponding items listed here for consideration were extracted from a systematic scoping review of 115 publications.

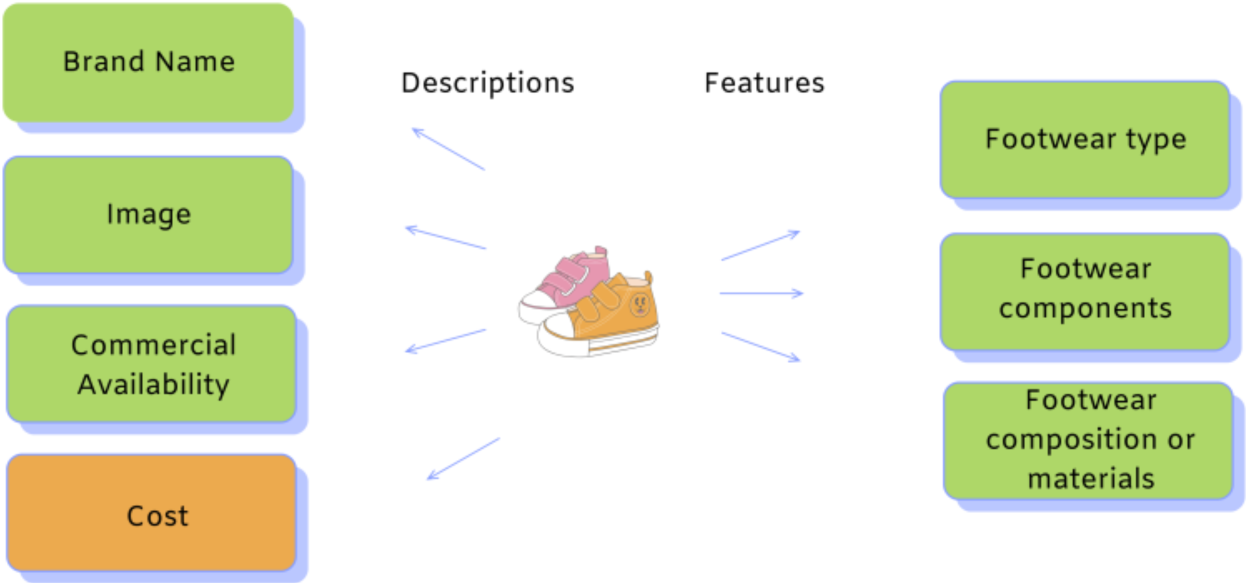

When you were asked about footwear descriptions and features, the following overarching domains reached consensus (70% or more agreed):

- **Brand name**
- **Image of footwear**
- **Commercial availability**
- **Footwear type**
- **Components of the footwear**
- **Composition or materials of the footwear**

There was one element reaching 50-69% agreement, please rate your agreement that this overarching domain should remain in the final recommendations.

	Strongly disagree	Disagree	Agree	Strongly Agree
Footwear retail cost	○	○	○	○

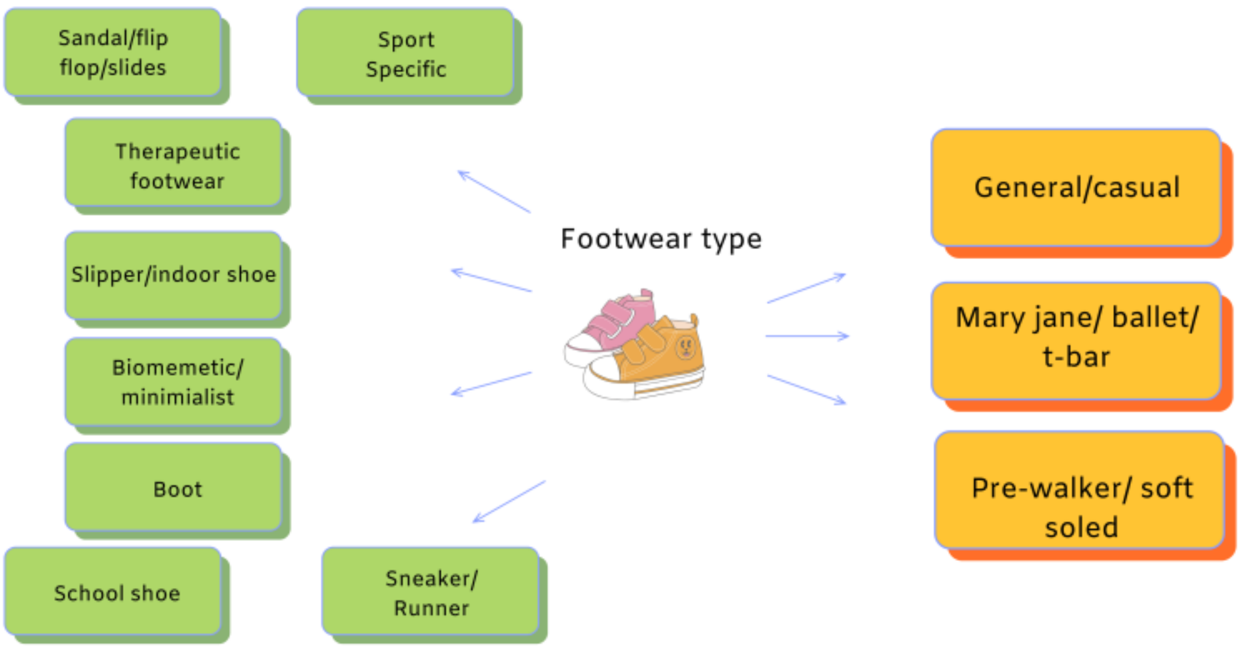

When you were asked about footwear features, the following type names reached consensus (70% or more agreed):

- Sandal/flip flop/slides
- Sport specific
- Therapeutic footwear (inclusive of medical and orthopaedic shoe)
- Slipper/indoor shoe
- Biomimetic/minimalist
- Boot
- School shoe
- Sneaker/runner

There were three types reaching 50-69% agreement, please rate your agreement that this footwear type name should remain in the final recommendations.

	Strongly disagree	Disagree	Agree	Strongly Agree
General/casual	○	○	○	○
Mary jane/ ballet/ t-bar	○	○	○	○
Pre-walker/soft soled	○	○	○	○

If you disagree with any of the above footwear type name, you can provide your reason or alternative wording suggestions if you wish.

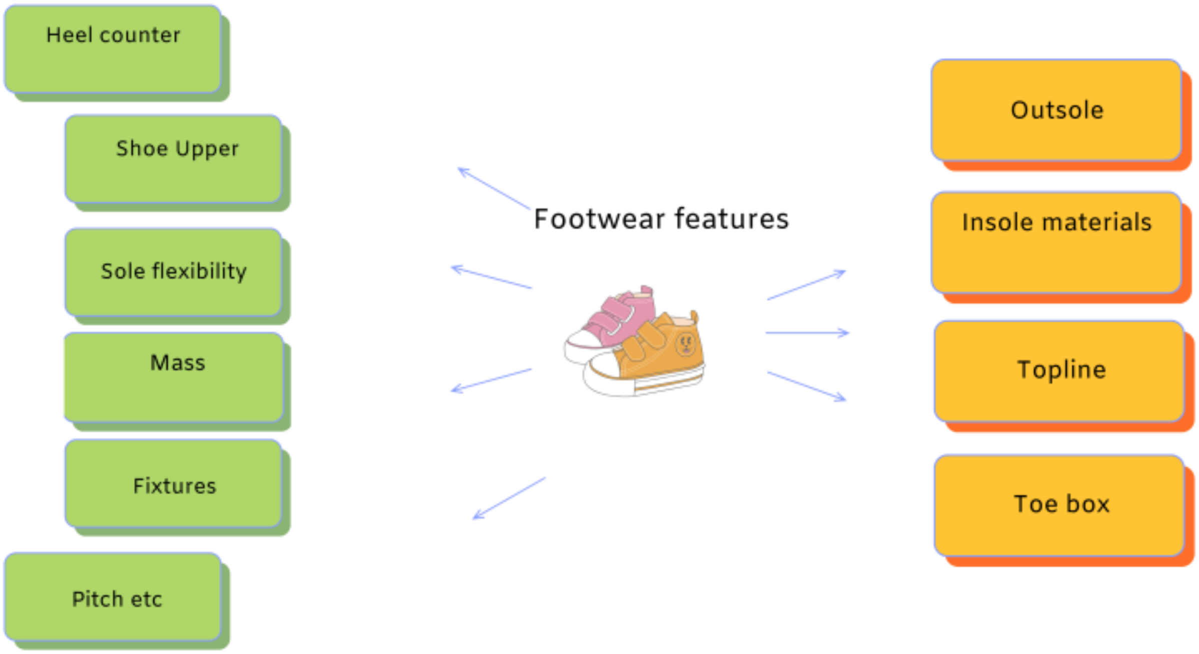

When you were asked about footwear features, the following footwear feature names reached consensus (70% or more agreed):

- Heel counter presence and/or stiffness
- Upper shoe covers full or part of foot
- Sole flexibility
- Mass of shoe
- Fixtures (eg velcro, laces etc) of shoe
- Pitch drop and/or stack of outsole

There were three features researching 50-69% agreement, please rate your agreement that this feature name should remain in the final recommendations.

	Strongly Disagree	Disagree	Agree	Strongly Agree
Outsole with/without separate heel	○	○	○	○

Insole materials in shoe	○	○	○	○
Topline of shoe in relation to the ankle	○	○	○	○
Toe box (shape and/or height) of upper	○	○	○	○

If you disagree with any of the above footwear features, you can provide your reason or alternative wording suggestions if you wish.

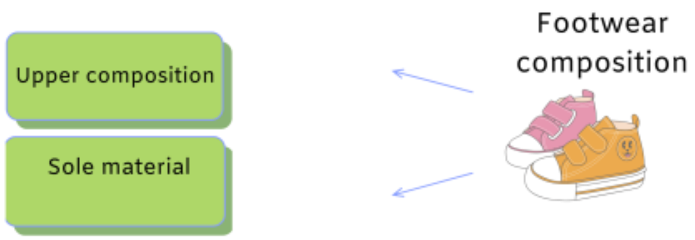

When you were asked about footwear composition, the following footwear composition elements names reached consensus (70% or more agreed):

- Upper material
- Sole material and density

There are no other elements to consider in this theme.

Outcomes

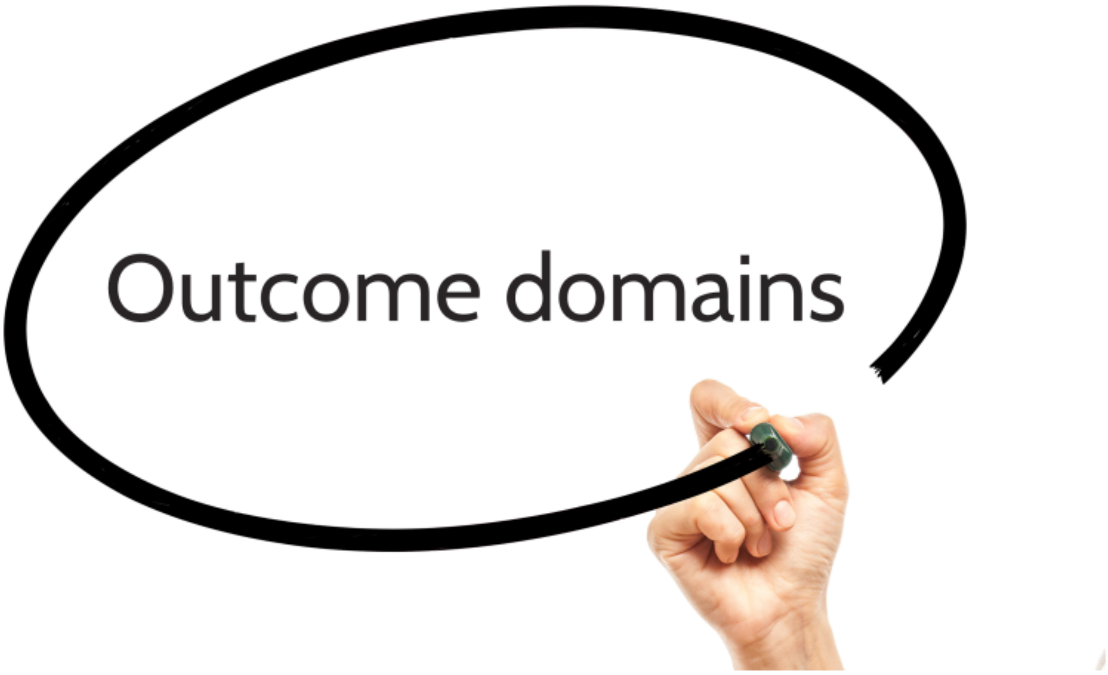

Aim 2: Minimum outcomes relating to the impact of children's footwear that should be reported in research.

We have organised outcomes against the "The 'F-words' in Childhood Disability" (Ref: Rosenbaum P, Child: care, health and development. 2012 Jul;38(4):457-63.) and this graphic is to remind you about this framework.

We had some minor feedback about alignment of particular outcomes with the "F-word" heading and have reviewed against literature within this framework to ensure it fit. No changes were made as all aligned within current literature.

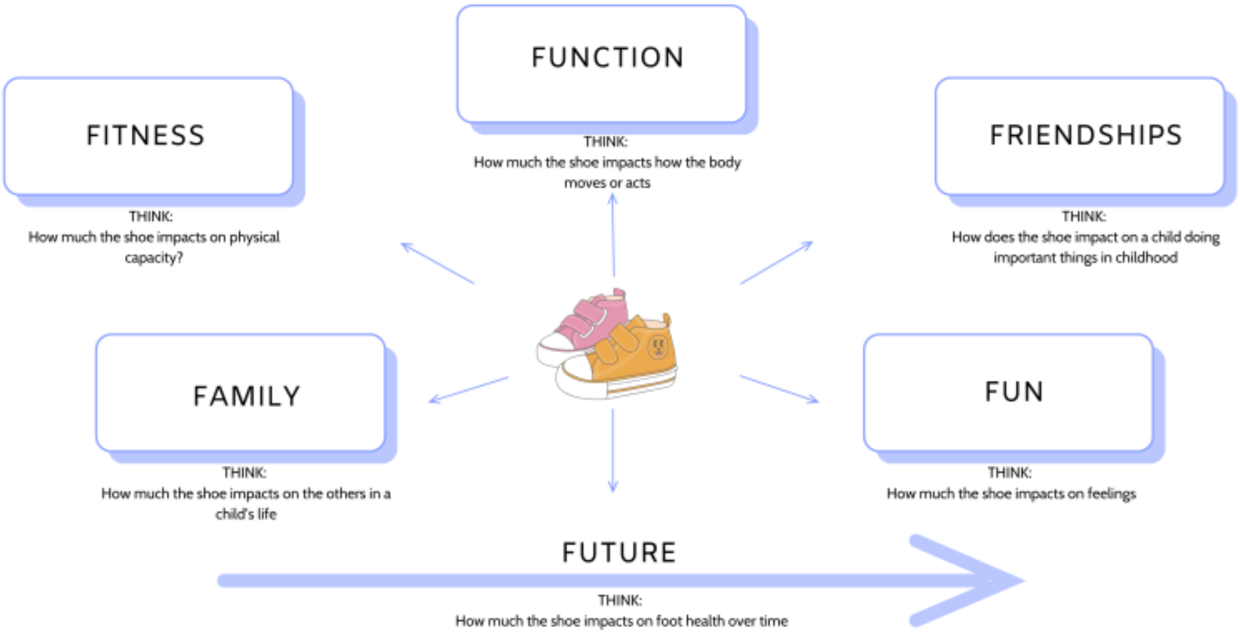

Similar to the information you have already seen about footwear, you will be presented with information relating to each of the above domains, the consensus and agreement. You will now only be asked to provide agreement, remembering to consider if the element should be included in a minimum dataset.

You will also be provided with new elements where participants introduced a proposed missing factor. You will have the opportunity to also provide comments.

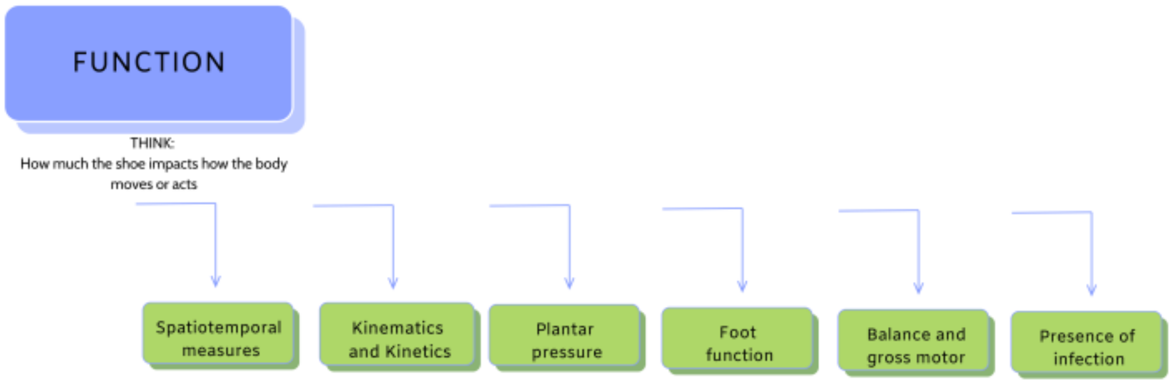

All six themes aligning to the **Functional** domain made consensus.

You will now be presented with the different elements under each theme.

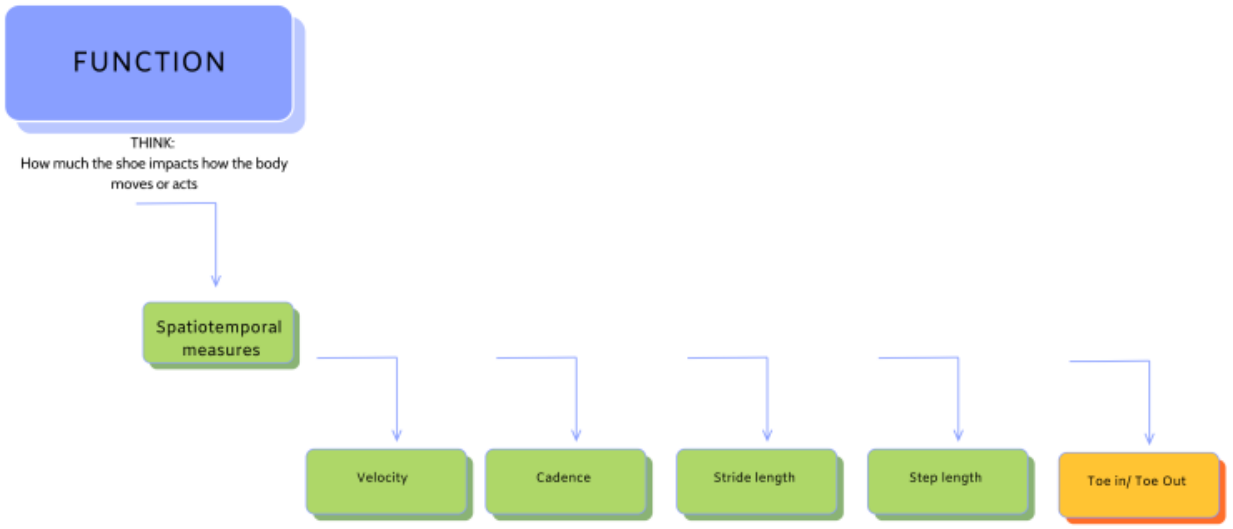

When you were asked about **Spatiotemporal** measures, the following

reached consensus (70% or more agreed):

- Velocity
- Cadence
- Stride length
- Step length

There was one element reaching 50-69% agreement, please rate your agreement that this element should remain in the final recommendations.

	Strongly Disagree	Disagree	Agree	Strongly Agree
Toe in/Toe out angle	○	○	○	○

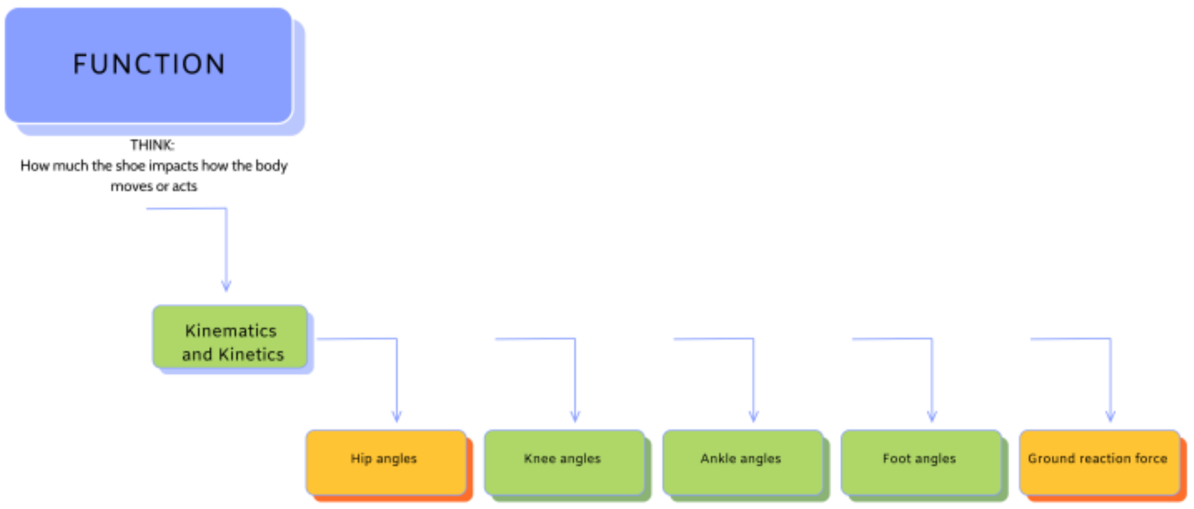

When you were asked about **Kinematic and Kinestic** measures, the following reached consensus (70% or more agreed):

- Knee joint angles
- Ankle joint angles
- Foot angles

There were two elements reaching 50-69% agreement, please rate your agreement that these elements should remain in the final recommendations.

	Strongly Disagree	Disagree	Agree	Strongly Agree
Hip joint angles	○	○	○	○
Ground Reaction Force	○	○	○	○

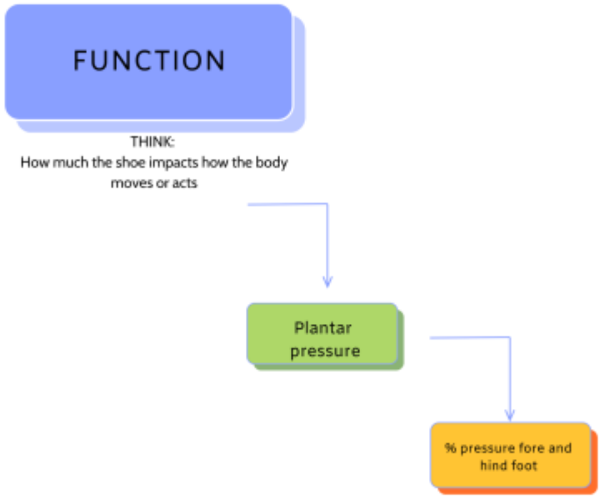

There was one element reaching 50-69% agreement in **Plantar Pressure** measures, please rate your agreement that this element should remain in the final recommendations.

	Strongly disagree	Disagree	Agree	Strongly agree
% pressure fore and hind foot	○	○	○	○

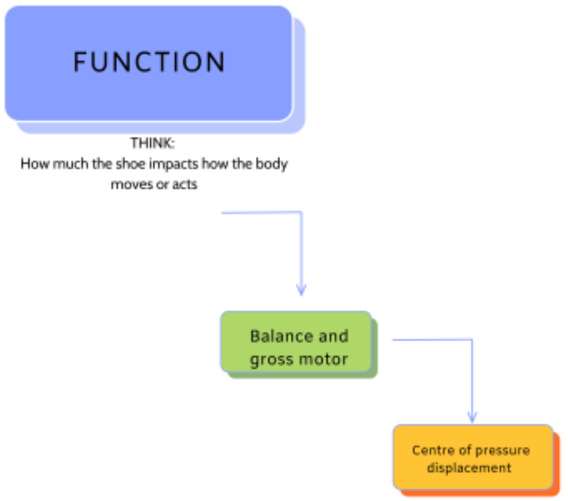

When you were asked about **Balance and Gross Motor** measures, there was one element reaching 50-69% agreement.

Please rate your agreement that this element should remain in the final recommendations.

	Strongly Disagree	Disagree	Agree	Strongly Agree
Centre of pressure displacement	○	○	○	○

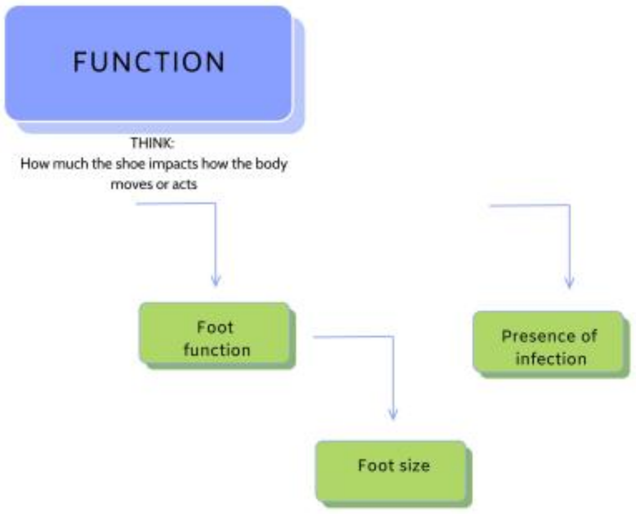

These domains and element met consensus.

If you have disagreed with any of the FUNCTION outcome responses, you can provide comment if you wish:

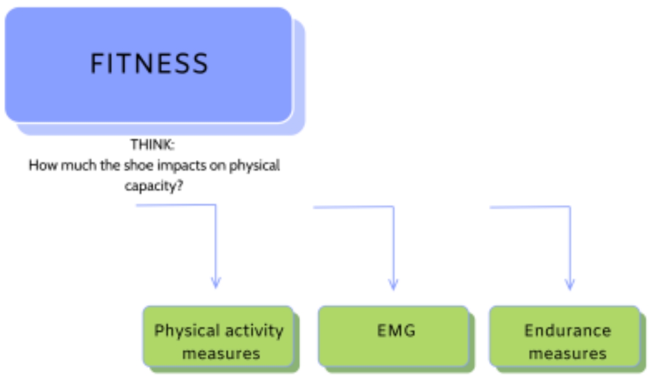

All three themes aligning to the functional domain made consensus.

You will now be presented with the different elements under each domain except for EMG which met consensus with no additional element.

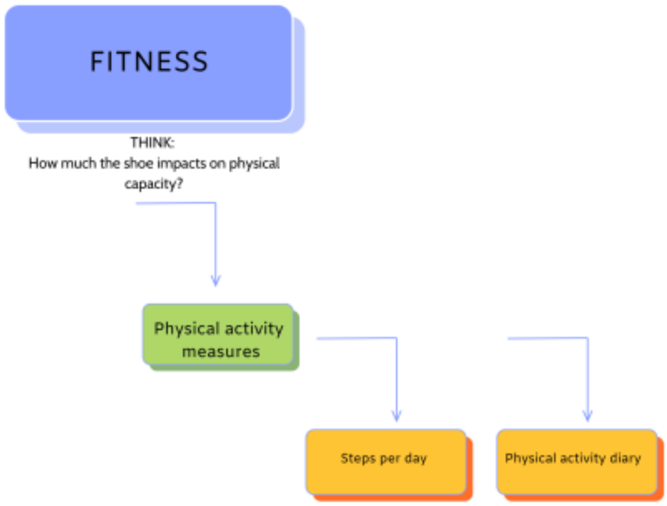

When you were asked about **Physical Activity** measures, there were two elements reaching 50-69% agreement.

Please rate your agreement that these elements should remain in the final recommendations.

	Strongly Disagree	Disagree	Agree	Strongly Agree
Steps per day	◯	◯	◯	◯
Physical Activity Diary	◯	◯	◯	◯

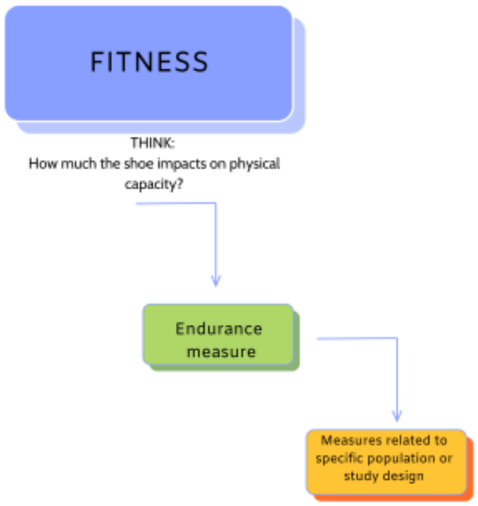

When you were asked about Endurance measures, there was no elements reaching agreement.

Please rate your agreement for the following statement to appear next to Endurance measure:

There were no endurance measures reaching consensus or agreement. Researchers should consider endurance measures related to specific populations or study designs.

	Strongly disagree	Disagree	Agree	Strongly agree
Above statement	○	○	○	○

If you have disagreed with the statement or any of the other elements, you can provide your reason or alternative wording suggestions if you wish.

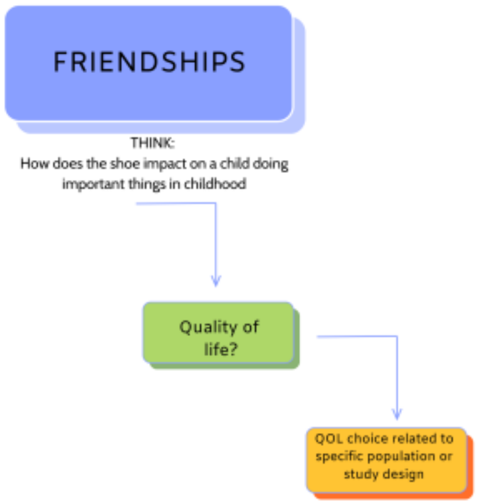

When you were asked about quality of life tools, there was no tools or elements reaching agreement.

Please rate your agreement for the following statement to appear next to quality of life tools:

There were no quality of life tools reaching consensus or agreement. Researchers should consider Quality of life measures related to specific populations or study designs.

	Strongly disagree	Disagree	Agree	Strongly agree
Above statement	○	○	○	○

If you have disagreed with the statement, please comment on why or an alternative below:

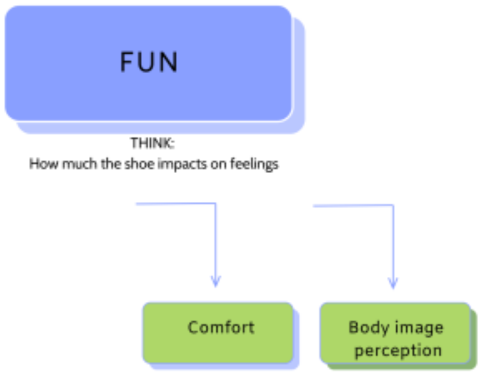

Both two themes aligning to the functional domain made consensus.

You will now be presented with the different elements under each comfort. There was no element for body image perception as it was a qualitative response.

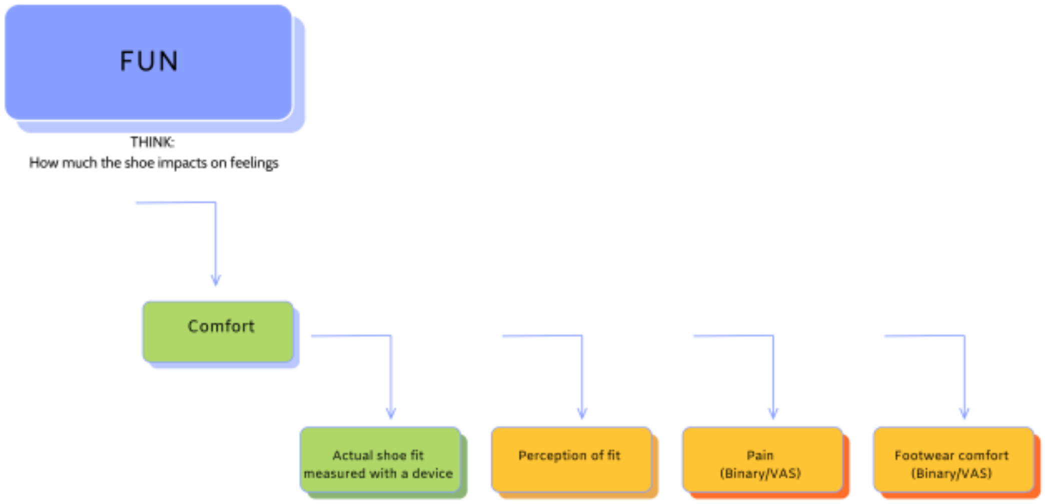

When you were asked about the comfort element, the following reached consensus (70% or more agreed):

- Actual Shoe Fit (as measured by a device)

There were three elements reaching 50-69% agreement, please rate your agreement that this element should remain in the final recommendations.

	Strongly Disagree	Disagree	Agree	Strongly Agree
Perception of fit	○	○	○	○
Pain (binary/VAS)	○	○	○	○
Footwear comfort (Binary/VAS)	○	○	○	○

If you have disagreed with any of the FUN outcome domains and responses, you can provide comment if you wish:

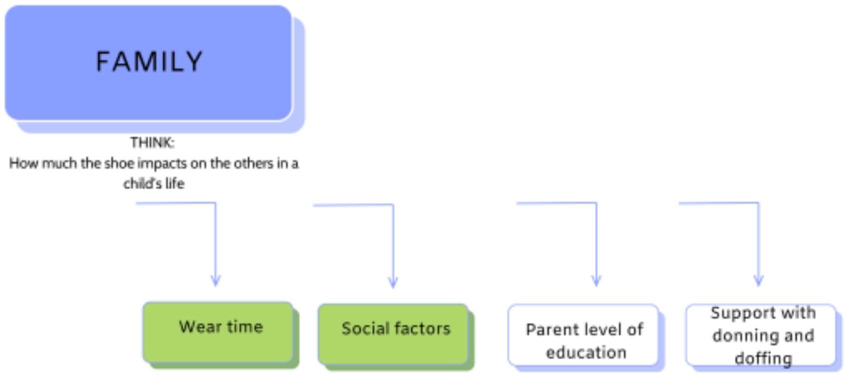

The two themes aligning to the **Family** domain made consensus.

- Wear time

- Social factors

Two additional themes have been suggested as aligning with the **Family** domain.

Please rate your agreement that these two themes should be aligned with the **Family** domain.

	Strongly Disagree	Disagree	Agree	Strongly Agree
Parent level of education	○	○	○	○
Support with donning and doffing footwear	○	○	○	○

If you have disagreed with any of the FAMILY outcome domains and responses, you can provide comment if you wish:

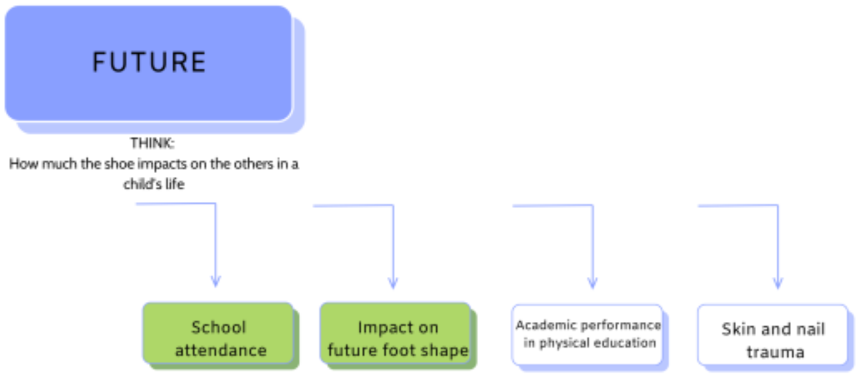

The two themes aligning to the **Future** domain made consensus.

- School attendance
- Impact on future foot shape

Two additional themes have been suggested as aligning with the **Future** domain.

Please rate your agreement that these two themes should be aligned with the **Future** domain.

	Strongly Disagree	Disagree	Agree	Strongly Agree
Academic performance in Physical Education	○	○	○	○
Skin and nail trauma linked to long term footwear use	○	○	○	○

If you have disagreed with any of the FUTURE outcome domains and responses, you can provide comment if you wish:

Powered by Qualtrics
