# Supplementary material for: Development of the CHILD‐SHOE Reporting Checklist: A Scoping Review and Modified Delphi Study to Support Reporting in Children's Footwear Research
Source: J Foot Ankle Res. 2025 Jul 9;18(3):e70065. doi: 10.1002/jfa2.70065 (PMC12241440; doi:10.1002/jfa2.70065)
Supplement: Supplementary file 5 — Supporting Information S5 [file JFA2-18-e70065-s006.pdf]

INTRO/CONSENT

CHILD's SHOE REPORT: A checklist of Information on children's footwear research reporting- Round 3

What does my participation involve in this round?

We have collated the responses from the second round.

We had 29 (91% of n=32) people take part in Round 2. This is now the third and final round.

Where there was 70% or greater agreement of responses, we included the domain and/or elements.

Where greater than 50%-69% of people agreed, we have included it as a statement for you to rate how much you agree in this final round. Where less than 50% of people responded the similarly, this has not been included in this round.
Items **not** meeting agreement in this round will not be in the final minimum recommended data set.

Where you told us you thought the element was appropriate but not the wording, we have also included the alternative wording for you to rate your preference. If this item reaches 70% or greater, we will include with the higher rated wording.

Please remember, this is about setting the "minimum footwear descriptions and outcomes" as a guide for researchers undertaking research about children's footwear research. It doesn't mean that people couldn't use these descriptions or terminology, just that these are preferred part of a minimum checklist researchers should include.

You can download a copy of the item progression and % of agreement at this link to guide your responses and see what did and didn't make this round: Round 3 child shoe

To meet the aim of this research, it is very important you complete this survey to give us your opinion of the statements. This is also essential for you to complete this if you have indicated you wish to contribute to authorship on a final version of a consensus statement.

It should take under 10 minutes.

If you would like a copy of your original responses in Round 2, please contact Jessica: Jessica.Kolic@monash.edu

Please provide your email below so we can track responses and link them between each round. Please use your same email for each round.

Footwear

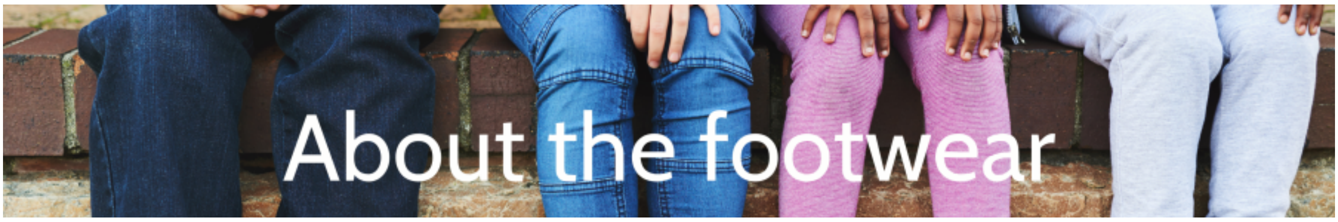

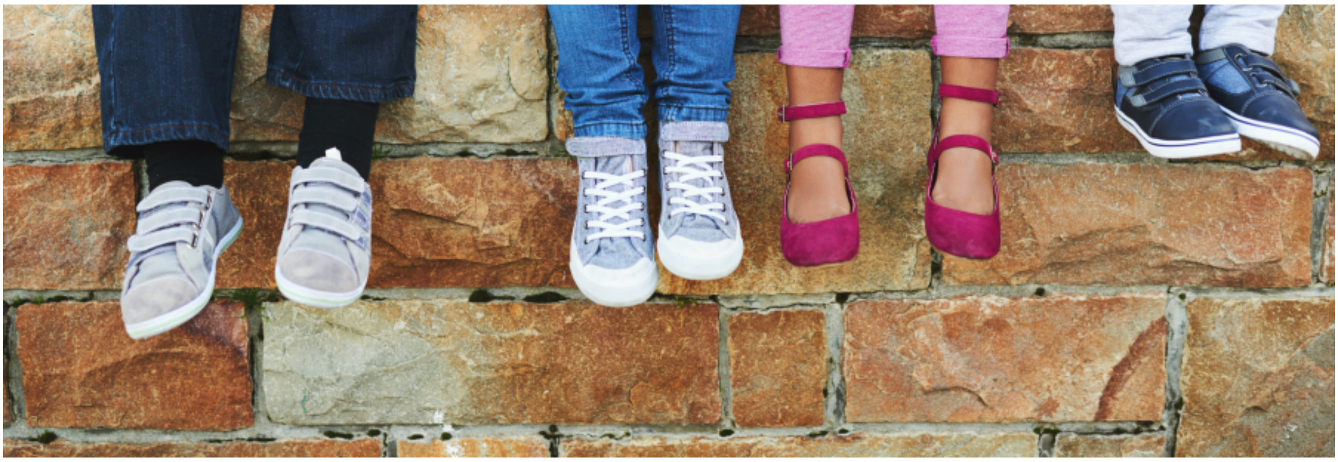

Aim 1: Minimum footwear descriptions and features that should be reported in research.

This section includes items related to footwear descriptors and features that have been organised into domains where possible.

Individual items have been identified where applicable within each domain.

All domains and corresponding items listed here for consideration were extracted from a systematic scoping review of 115 publications.

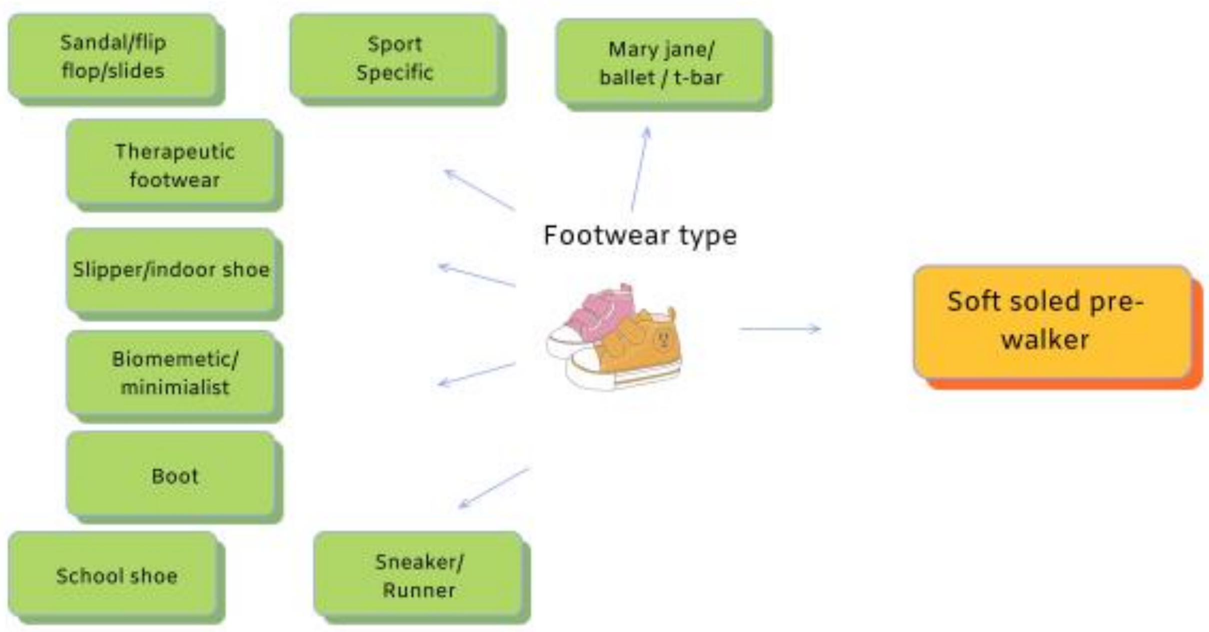

When you were asked about footwear features, the following type names reached consensus (70% or more agreed):

- Sandal/flip flop/slides
- Sport specific
- Therapeutic footwear (inclusive of medical and orthopaedic shoe)
- Slipper/indoor shoe
- Biomimetic/minimalist
- Boot
- School shoe
- Sneaker/runner
- Mary jane/Ballet

There were was one type reaching 50-69% agreement, however an alternative was also proposed, please rate your agreement that this footwear type name should remain in the final recommendations.

	Strongly disagree	Disagree	Agree	Strongly Agree
Soft-soled pre-walker	○	○	○	○
Pre-walker/Soft-soled	○	○	○	○

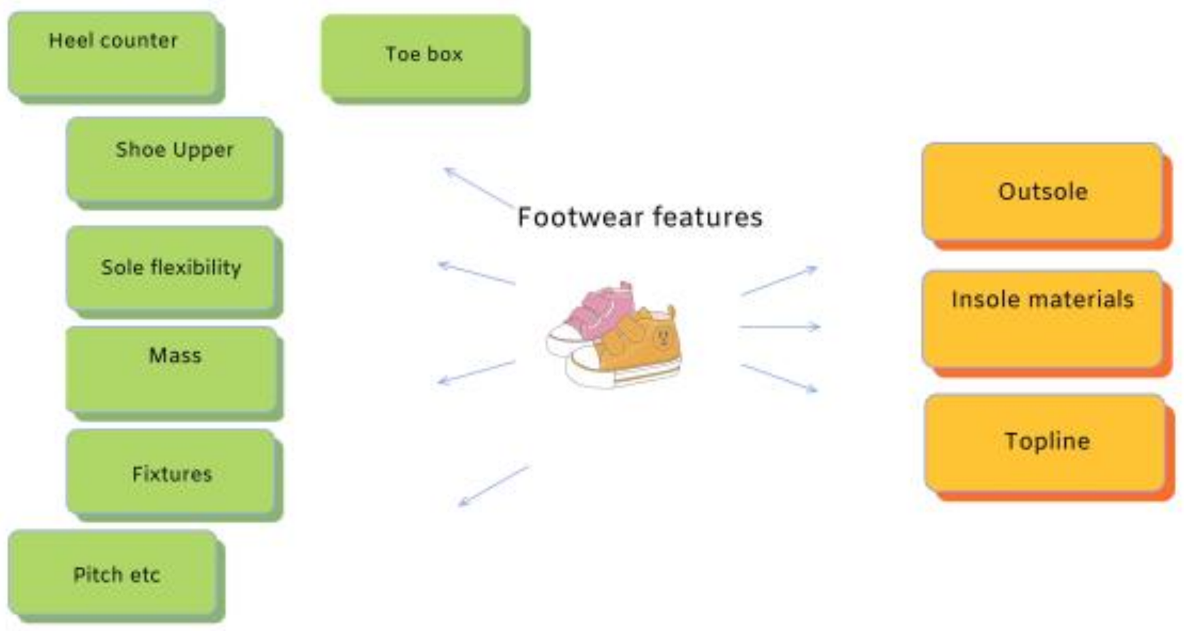

When you were asked about footwear features, the following footwear feature names reached consensus (70% or more agreed):

- Heel counter presence and/or stiffness
- Upper shoe covers full or part of foot
- Sole flexibility
- Mass of shoe
- Fixtures (eg velcro, laces etc) of shoe
- Pitch drop and/or stack of outsole
- Toe box (shape and/or height) of upper

There were three features researching 50-69% agreement, please rate your agreement that this feature name should remain in the final recommendations.

	Strongly Disagree	Disagree	Agree	Strongly Agree
Outsole with/without separate heel	○	○	○	○
Insole materials in shoe	○	○	○	○
Topline of shoe in relation to the ankle (eg high, mid or low cut)	○	○	○	○
Topline of shoe in relation to the ankle	○	○	○	○

Outcomes

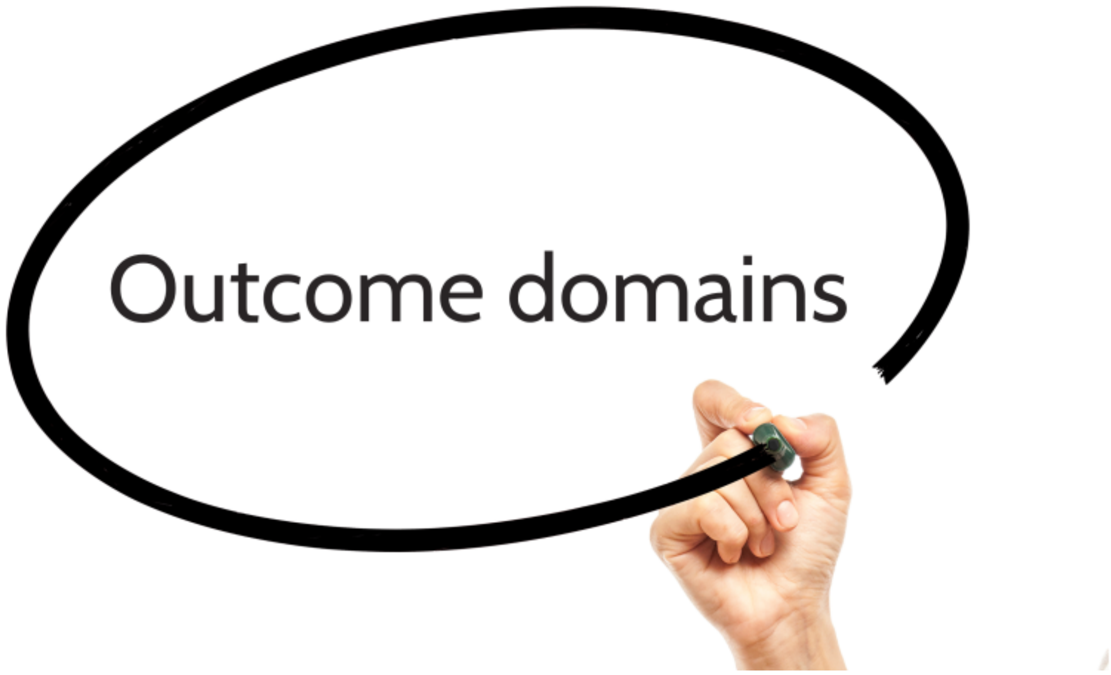

Aim 2: Minimum outcomes relating to the impact of children's footwear that should be reported in research.

We have organised outcomes against the "The 'F-words' in Childhood Disability" (Ref: Rosenbaum P, Child: care, health and development. 2012 Jul;38(4):457-63.) and this graphic is to remind you about this framework.

We had some minor feedback about alignment of particular outcomes with the "F-word" heading and have reviewed against literature within this framework to ensure it fit. No changes were made as all aligned within current literature.

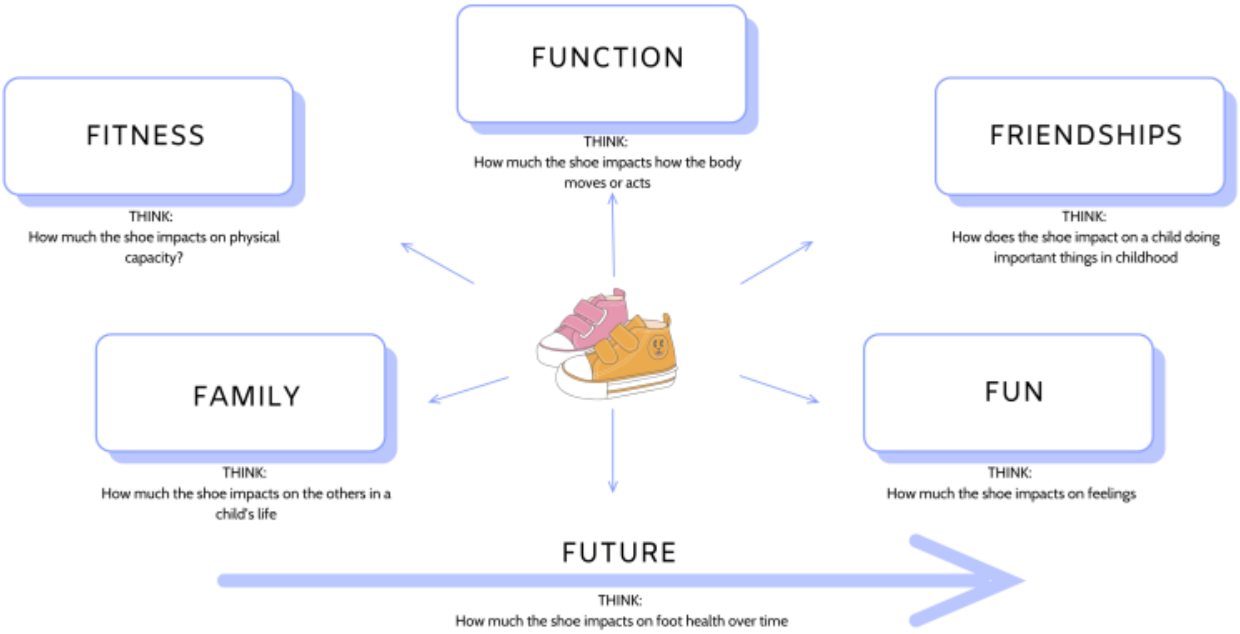

Similar to the information you have already seen about footwear, you will be presented with information relating to each of the above domains, the consensus and agreement. You will now only be asked to provide agreement, remembering to consider if the element should be included in a **minimum dataset**.

By minimum - we mean something that should ALWAYS be collected if collecting data about the domain. It doesn't mean that a researcher couldn't report on more variables if they had equipment providing reliable measures, but at a <u>minimum</u>, they should collect this information and report on it.

To keep the survey short, we have not repeated any outcomes, domains or elements where you have seen it met consensus

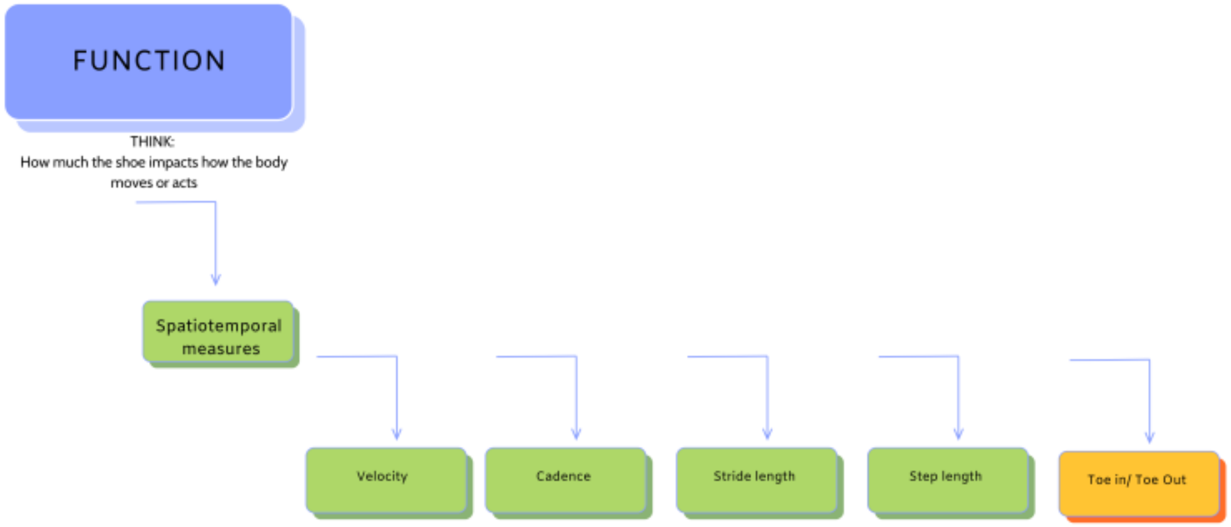

When you were asked about **Spatiotemporal** measures, the following reached consensus (70% or more agreed):

- Velocity
- Cadence
- Stride length
- Step length

There was one element reaching 50-69% agreement, please rate your agreement that this element should remain in the final recommendations.

	Strongly Disagree	Disagree	Agree	Strongly Agree

Toe in/Toe out angle as measured with an valid method or device	○	○	○	○
Toe in/Toe out angle	○	○	○	○

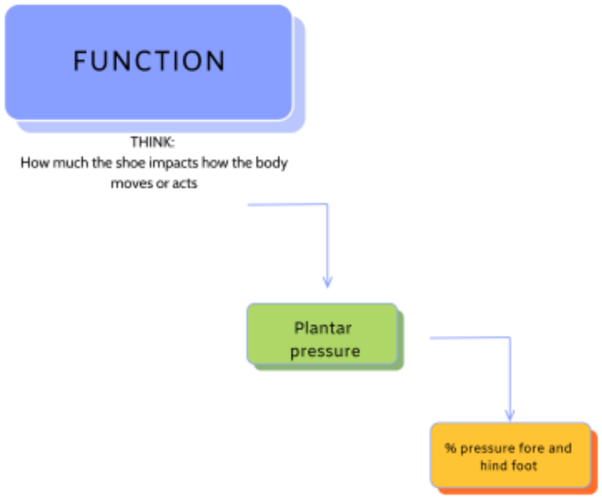

There was one element reaching 50-69% agreement in **Plantar Pressure** measures, please rate your agreement that this element should remain in the final recommendations.

	Strongly disagree	Disagree	Agree	Strongly agree
% pressure fore and hind foot	○	○	○	○

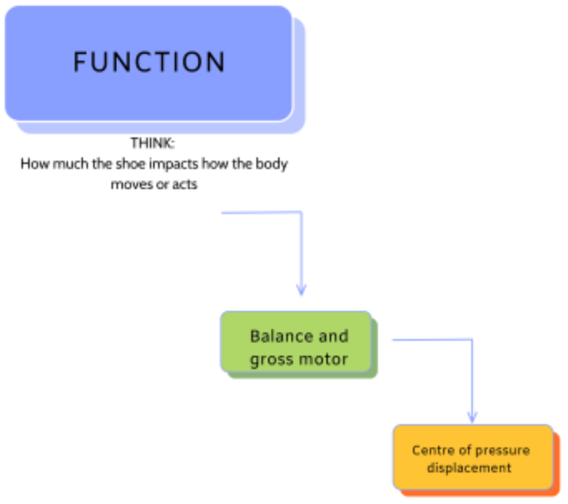

When you were asked about **Balance and Gross Motor** measures, there was one element reaching 50-69% agreement.

Please rate your agreement that this element should remain in the final recommendations.

	Strongly Disagree	Disagree	Agree	Strongly Agree
Centre of pressure displacement	○	○	○	○

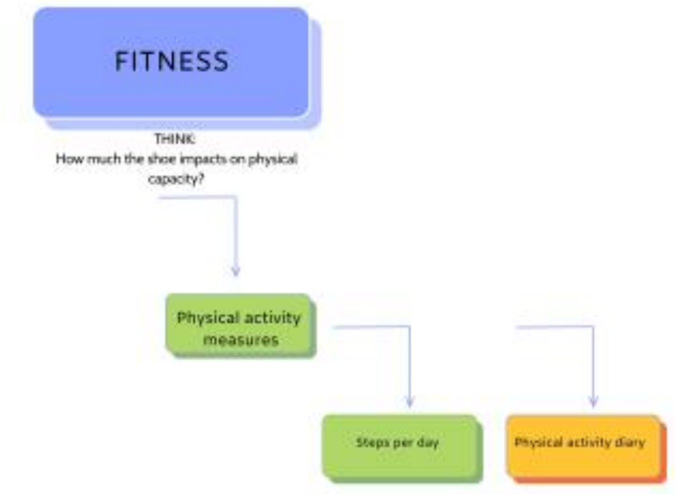

When you were asked about **Physical Activity** measures, 70% or more agreed with:
- Steps per day

There was one element reaching 50-69% agreement.

Please rate your agreement that this element should remain in the final recommendations.

	Strongly Disagree	Disagree	Agree	Strongly Agree
Physical Activity Diary	○	○	○	○

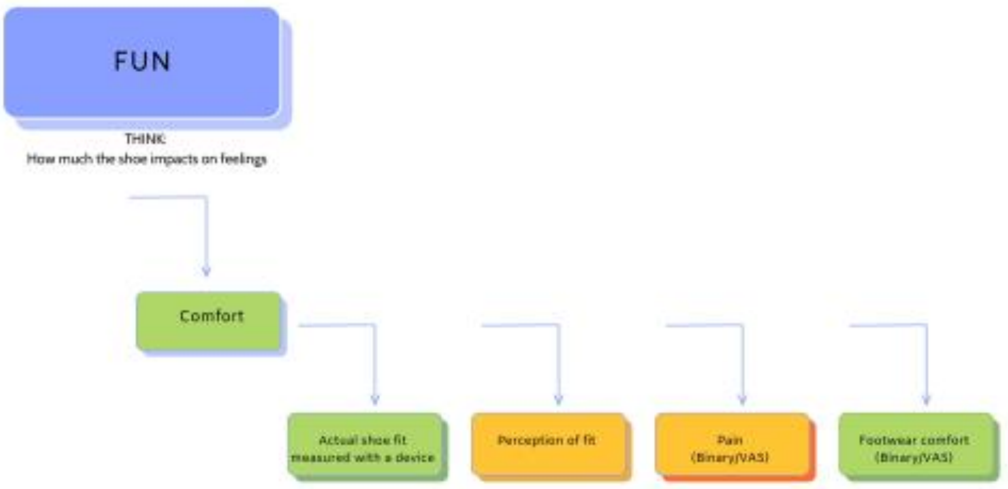

When you were asked about the comfort element, the following reached consensus (70% or more agreed):

- Actual Shoe Fit (as measured by a device)
- Footwear comfort (binary/VAS)

There were two elements reaching 50-69% agreement, please rate your agreement that these elements should remain in the final recommendations.

	Strongly Disagree	Disagree	Agree	Strongly Agree
Self reported perception of shoe fit (Binary/VAS)	○	○	○	○
Perception of shoe fit	○	○	○	○
Pain (Binary/VAS)	○	○	○	○

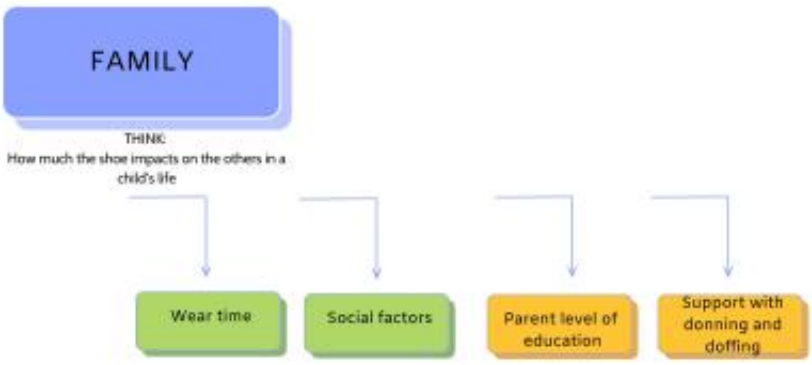

The two themes aligning to the **Family** domain made consensus.

- Wear time
- Social factors

There were two themes reaching 50-69% agreement, please rate your agreement that these elements should remain in the final recommendations.

Please rate your agreement that these two themes should be aligned with the **Family** domain.

	Strongly Disagree	Disagree	Agree	Strongly Agree
Parent level of education	○	○	○	○
Requires support with putting on and taking off footwear (Binary)	○	○	○	○
Support required for donning and doffing	○	○	○	○

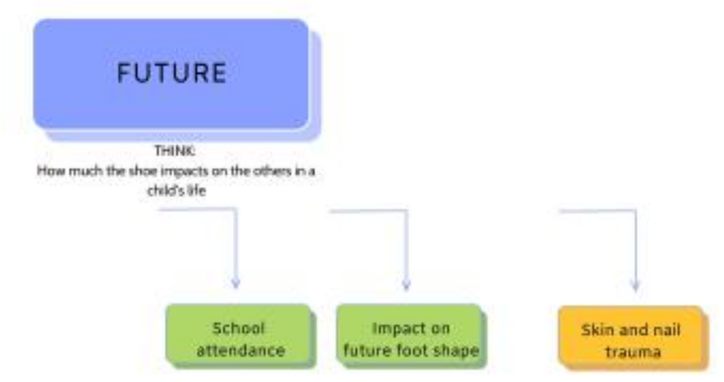

The two themes aligning to the **Future** domain made consensus.

- School attendance
- Impact on future foot shape

There were one theme reaching 50-69% agreement, please rate your agreement that this should remain in the final recommendations.

Please rate your agreement that this theme should be aligned with the **Future** domain.

	Strongly Disagree	Disagree	Agree	Strongly Agree
Skin and nail trauma linked to long term footwear use	○	○	○	○

Powered by Qualtrics
