# Supplementary material for: Development of the CHILD‐SHOE Reporting Checklist: A Scoping Review and Modified Delphi Study to Support Reporting in Children's Footwear Research
Source: J Foot Ankle Res. 2025 Jul 9;18(3):e70065. doi: 10.1002/jfa2.70065 (PMC12241440; doi:10.1002/jfa2.70065)
Supplement: Supplementary file 6 — Supporting Information S6 [file JFA2-18-e70065-s001.doc]

CHILD-SHOE Reporting Checklist (2025

| Domain | Item No | Element or outcome | Reported on page number |
| --- | --- | --- | --- |
| Footwear description | | | |
| Brand | 1 | The brand and model name/number of the footwear | |
| Image | 2 | A clear image displaying key features |  |
| Availability | 3 | Description of commercial availability including country/ies of availability |  |
| **Footwear features** |  | *Footwear descriptions: type, features and components are clearly explained using internationally recognisable common terminology.* |  |
|  |  |  |  |
| Footwear type | 4 | *Name the type/s of footwear using a naming convention from the following list:* |  |
|  | 4a | Sandal, flip flops or slides |  |
|  | 4b | Slipper or indoor shoe |  |
|  | 4c | Biomimetic, functional or minimalist shoe |  |
|  | 4d | Mary Jane, ballet flat or t-bar shoe |  |
|  | 4e | Boot |  |
|  | 4f | School shoe (oxford style) |  |
|  | 4g | Therapeutic footwear (medical/orthopaedic) |  |
|  | 4h | Sport specific shoe (must specify sport) |  |
|  | 4i | Sneaker, runner, trainer or sport/athletic shoe |  |
|  | 4j | Other type (provide clear description) |  |
| Footwear components | 5 | *Describe* ***all*** *footwear components from the following list:* |  |
|  | 5a | Heel counter presence and/or it’s stiffness |  |
|  | 5b | Upper of shoe covers full or part of foot |  |
|  | 5c | Sole flexibility |  |
|  | 5c | Fixtures (e.g.velcro, laces etc) of shoe |  |
|  | 5d | Topline of shoe in relation to the ankle (eg high, mid or low cut)* |  |
|  | 5e | Mass (e.g., grams) of shoes |  |
|  | 5f | Toe box (shape and/or height) of upper |  |
|  | 5g | Pitch, drop and/or stack of outsole |  |
|  | 5h | Additional footwear components described as required |  |
| Composition | 6 | *Describe* ***all*** *footwear composition elements from the following list:* |  |
|  | 6a | Upper material |  |
|  | 6b | Sole material |  |
|  | 6c | Other footwear composition or materials features are described |  |
| **Outcome measures** |  |  |  |
| Domain description^α^ | 7 | *Identify the domain/s that align with chosen outcome measure/s* |  |
|  | 7a | Function domain |  |
|  | 7b | Fitness domain |  |
|  | 7c | Friendship domain |  |
|  | 7d | Fun domain |  |
|  | 7e | Family domain |  |
|  | 7f | Future domain |  |
| ***Function Domain*** |  |  |  |
| Spatiotemporal measures | 8 | *Where spatiotemporal measures are collected as part of FUNCTION,* ***all*** *outcomes from the following list are described* |  |
|  | 8a | Velocity (meters/second) |  |
|  | 8b | Cadence (steps/second) |  |
|  | 8c | Stride length (cm) |  |
|  | 8d | Step length (cm) |  |
|  | 8f | Other spatiotemporal measures are described |  |
| Kinematic and kinetic measures | 9 | *Where kinematic and kinetic measures are collected as part of FUNCTION,* ***all*** *outcomes from the following list are described:* |  |
|  | 9a | Hip joint angles |  |
|  | 9b | Knee joint angles |  |
|  | 9c | Ankle joint angles |  |
|  | 9d | Foot joint angles |  |
|  | 9e | Ground reaction force |  |
|  | 9f | Other kinematic and kinetic measures are described |  |
| Plantar pressure | 10 | *Where plantar measures are collected as part of FUNCTION, the measure and collection method is clearly described and relevant to the population or study design* |  |
| Foot features | 11 | *Where foot features measures are collected as part of FUNCTION,* ***all*** *outcomes from the following list are described:* |  |
|  | 11a | Foot size |  |
|  | 11b | Other foot features are described |  |
| Balance and gross motor | 12 | *Where balance and/or gross motor measures are collected as part of FUNCTION, the measure and collection method is clearly described and specific to the population or study design* |  |
| Infection | 13 | *Where infection measures are collected as part of FUNCTION, the measure and collection method is clearly described and specific to the population or study design* |  |
| ***Fitness domain*** |  |  |  |
| Physical activity | 14 | *Where physical activity measures are collected as part of FITNESS,* ***all*** *outcomes from the following list are described* |  |
|  | 14a | Steps per day (count) |  |
|  | 14b | Other physical activity measures are described |  |
| Electromyography (EMG) | 15 | EMG measures and collection method are clearly described and specific to the population and study design |  |
| Endurance | 15 | *Where measures are collected as part of ENDURANCE, the measure and collection method is clearly described and specific to the population or study design* |  |
| ***Friendship domain*** | 16 | *Where quality of life measures are collected as part of FRIENDSHIP DOMAIN, the measure and collection method is clearly described and specific to the population or study design* |  |
| ***Fun domain*** |  |  |  |
| Comfort | 17 | *Where comfort measures are collected as part of the FUN DOMAIN,* ***all*** *outcomes from the following list are described* |  |
|  | 17a | Shoe fit measured with a fit device |  |
|  | 17b | Footwear comfort (visual analogue scale mm) |  |
|  | 17c | Other comfort measures are described |  |
| Body Image | 18 | *Where any body image perception measures are collected as part of FUN DOMAIN, the measure and collection method is clearly described and specific to the population or study design* |  |
| ***Family domain*** | 19 | *Where FAMILY DOMAIN measures are collected,* ***all*** *outcomes from the following list are described* |  |
|  | 19 | Wear time (minutes or hours per day) |  |
|  | 19a | Other family related measures are described |  |
| ***Future domain*** | 20 | *Where FUTURE DOMAIN measures are collected,* ***any*** *outcomes from the following list are described* |  |
|  | 20a | School attendance over time |  |
|  | 20b | Longitudinal impact of footwear on foot shape over time |  |
|  | 20c | Other future measures are described |  |

Citation: INSERT ON PUBLICATION

*We strongly recommend using this checklist in collaboration with an appropriate guideline for the study design. This reporting checklist only relates to research where the population is children’s and footwear is the primary interest

^α^ Researchers are strongly encouraged to review and align their outcome measures with the child friendly classification domains outlined by CanChild: https://canchild.ca/en/research-in-practice/f-words-in-childhood-disability
